# Rhodium(iii)-catalyzed annulation of enamides with sulfoxonium ylides toward isoquinolines†

**DOI:** 10.1039/d1ra01063h

**Published:** 2021-03-19

**Authors:** Chao Hong, Shuling Yu, Zhanxiang Liu, Yuhong Zhang

**Affiliations:** Department of Chemistry, Zhejiang University Hangzhou 310027 People's Republic of China liuzhanx@zju.edu.cn yhzhang@zju.edu.cn; State Key Laboratory of Applied Organic Chemistry, Lanzhou University Lanzhou 730000 People's Republic of China

## Abstract

An efficient rhodium(iii)-catalyzed C–H activation followed by intermolecular annulation between enamides and sulfoxonium ylides has been developed. The transformation proceeds smoothly with a broad range of substrates, affording a series of isoquinoline derivatives in moderate to good yields under additive-free conditions.

## Abstract 2

An efficient rhodium(iii)-catalyzed C–H activation followed by intermolecular annulation between enamides and sulfoxonium ylides has been developed.

## Graphical abstract

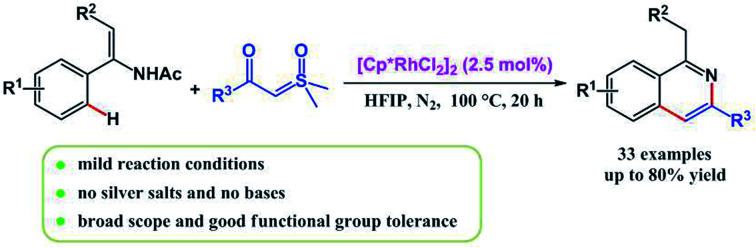

## Introduction

**Fig. 1:**
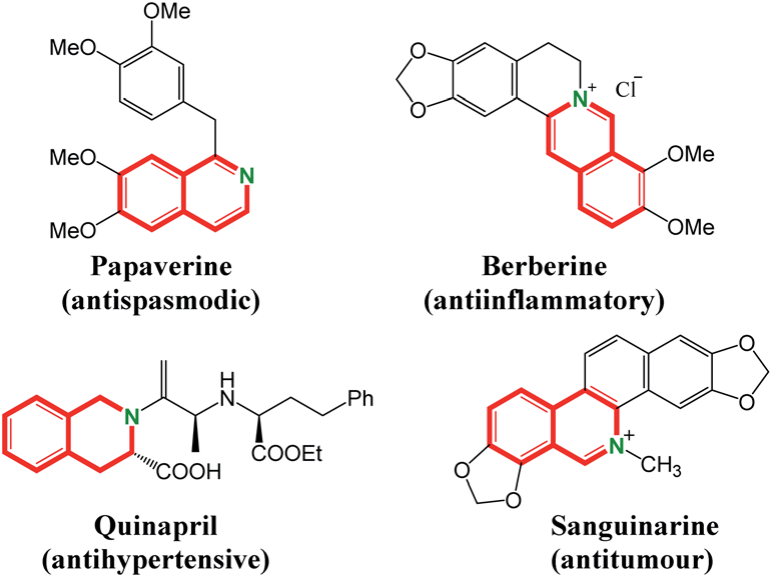
Representative bioactive isoquinolines.

**Scheme 1:**
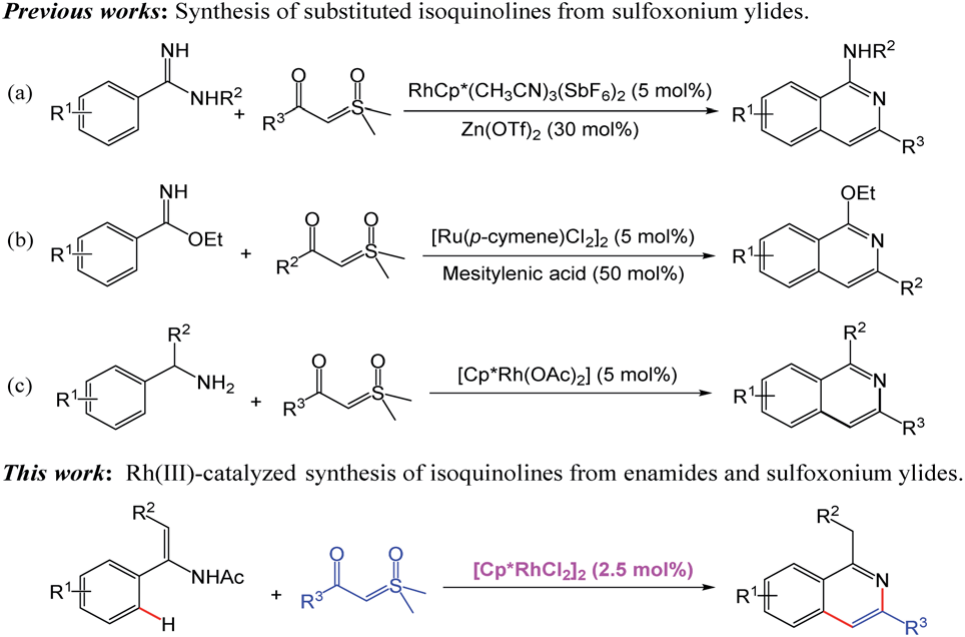
Synthetic strategies toward isoquinolines.

Isoquinolines represent important structural motifs frequently found in natural products, functional materials and pharmaceuticals.^1^ Their unusual bioactive properties, such as antispasmodic,^2^ antiinflammatory,^3^ antihypertensive^4^ and antitumour^5^ activities, have attracted much attention (Fig. 1). Consequently, the alternative efficient synthetic methodology for isoquinolines is of great importance.^6^ Traditional methods, including the famous Bischler–Napieralski,^7^ Pictet–Gams^8^ and Pictet–Spengler^9^ reactions, are well-established. Most of these protocols suffer from harsh reaction conditions and environmental problems. In this regard, transition metal-catalyzed C–H activation/annulation would be valuable and complementary to the known classic methodology, enabling the direct access to a series of isoquinoline derivatives with minimum environmental impact and fewer synthetic steps.^10–13^ On the other hand, sulfoxonium ylides are attractive starting materials that can be easily accessed and widely be utilized as transition metal-carbene precursors in coupling reactions.^14,15^ The use of sulfoxonium ylide in the synthesis of isoquinoline through Rh(iii)-catalyzed C–H activation was reported by Li and co-workers. In their transformation, the *ortho* C–H bond of amidines is activated by rhodium catalyst to give isoquinolines with sulfoxonium ylides in the presence of 30% Zn(OTf)_2_ (Scheme 1a).^16^ A ruthenium-catalyzed mono *ortho*-C–H annulation of benzimidates with sulfoxonium ylides was developed for the synthesis of substituted isoquinolines by Wang group in the presence of mesitylenic acid (Scheme 1b).^17^ Very recently, preparation of isoquinoline derivatives by Rh(iii)-catalyzed coupling reaction of benzylamine and sulfoxonium ylides using water as solvent is achieved by Wu and coworkers (Scheme 1c).^18^

Enamides are valuable building blocks in organic synthesis having tunable reactivity and potential usage in various transformations.^19^ The potential of enamide chemistry has been witnessed by transition-metal-catalyzed coupling reactions.^20^ As part of our continuing interest in metal-catalyzed enamide-directed C–H functionalization reaction and the synthesis of heterocyclic compounds,^21^ herein, we present a novel Rh(iii)-catalyzed cascade transformation from enamides and sulfoxonium ylides for the preparation of isoquinolines. This method allows the approach of a range of diversified 1,3-disubstituted isoquinolines with moderate to good yields under mild conditions without the use of additives.

## Results and discussion

We initiated our investigation on the model reaction of enamide (1a) and sulfoxonium ylide (2a) to optimize various reaction parameters. The results have been summarized in Table 1. At the outset, our study was treated by enamide (1a) with sulfoxonium ylide (2a) in the presence of [RhCp*Cl_2_]_2_/AgSbF_6_ and NaOAc in THF at 100 °C under N_2_ for 20 h (entry 1, Table 1). However, the target product 3aa was not detected in the reaction. Subsequently, when different solvents such as DMF, DCE and HFIP were studied on this reaction (entry 2–4, Table 1), we were pleased to find that the reaction occurred in HFIP, affording the desired annulation product 3aa in a yield of 25%. The experimental results show that the protic solvent-HFIP has a better promotion effect on the reaction. Different inorganic bases (LiOAc, KOAc, CsOAc and Cu(OAc)_2_) were then screened (entry 5–8, Table 1), but the results were no better than that obtained with NaOAc (entry 4, Table 1). The yield was improved to 46% when AgSbF_6_ was removed (compare entry 9 with entry 4, Table 1). Next, the yield increased to 71% in the absence of NaOAc (compare entry 14 with entry 9, Table 1). The other transition metal catalysts such as [Rh(cod)Cl]_2_ and RhCl_3_ were probed as well. The reaction results indicated that they were less effective than [RhCp*Cl_2_]_2_ (entry 15–16, Table 1). Control experiment showed that an absence of the Rh catalyst led to no formation of 3aa (entry 17, Table 1). The temperature reduction (80 °C) or elevation (120 °C) had an adverse effect (entry 18–19, Table 1). Therefore, the best result (71%) was achieved by using [RhCp*Cl_2_]_2_ (2.5 mol%) in HFIP at 100 °C under N_2_ for 20 h.

**Table 1:** Optimization of the reaction conditions^a^

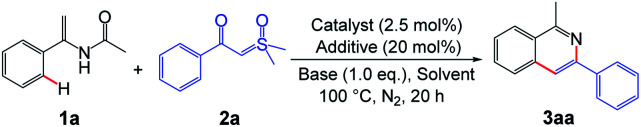				
Entry	Catalyst	Additive/base	Solvent	Yield^b^ (%)
1	[RhCp*Cl_2_]_2_	AgSbF_6_/NaOAc	THF	N.R.
2	[RhCp*Cl_2_]_2_	AgSbF_6_/NaOAc	DMF	N.R.
3	[RhCp*Cl_2_]_2_	AgSbF_6_/NaOAc	DCE	N.R.
4	[RhCp*Cl_2_]_2_	AgSbF_6_/NaOAc	HFIP	25
5	[RhCp*Cl_2_]_2_	AgSbF_6_/LiOAc	HFIP	19
6	[RhCp*Cl_2_]_2_	AgSbF_6_/KOAc	HFIP	18
7	[RhCp*Cl_2_]_2_	AgSbF_6_/CsOAc	HFIP	20
8	[RhCp*Cl_2_]_2_	AgSbF_6_/Cu(OAc)_2_	HFIP	5
9	[RhCp*Cl_2_]_2_	—/NaOAc	HFIP	46
10	[RhCp*Cl_2_]_2_	—/LiOAc	HFIP	30
11	[RhCp*Cl_2_]_2_	—/KOAc	HFIP	28
12	[RhCp*Cl_2_]_2_	—/CsOAc	HFIP	29
13	[RhCp*Cl_2_]_2_	—/Cu(OAc)_2_	HFIP	12
**14**	[RhCp*Cl_**2**_ **]**_**2**_	**—**	**HFIP**	**71**
15	[Rh(cod)Cl]_2_	—	HFIP	N.R.
16	RhCl_3_	—	HFIP	N.R.
17	—	—	HFIP	N.R.
18^c^	[RhCp*Cl_2_]_2_	—	HFIP	53
19^d^	[RhCp*Cl_2_]_2_	—	HFIP	65

a Reaction conditions: 1a (0.3 mmol), 2a (0.2 mmol), [RhCp*Cl_2_]_2_ (2.5 mol%), additive (20 mol%), base (1.0 equiv.), solvent (2.0 mL), 100 °C, under N_2_, for 20 h. N.R. = no reaction.

b Isolated yields.

c 80 °C.

d 120 °C.

**Scheme 2:**
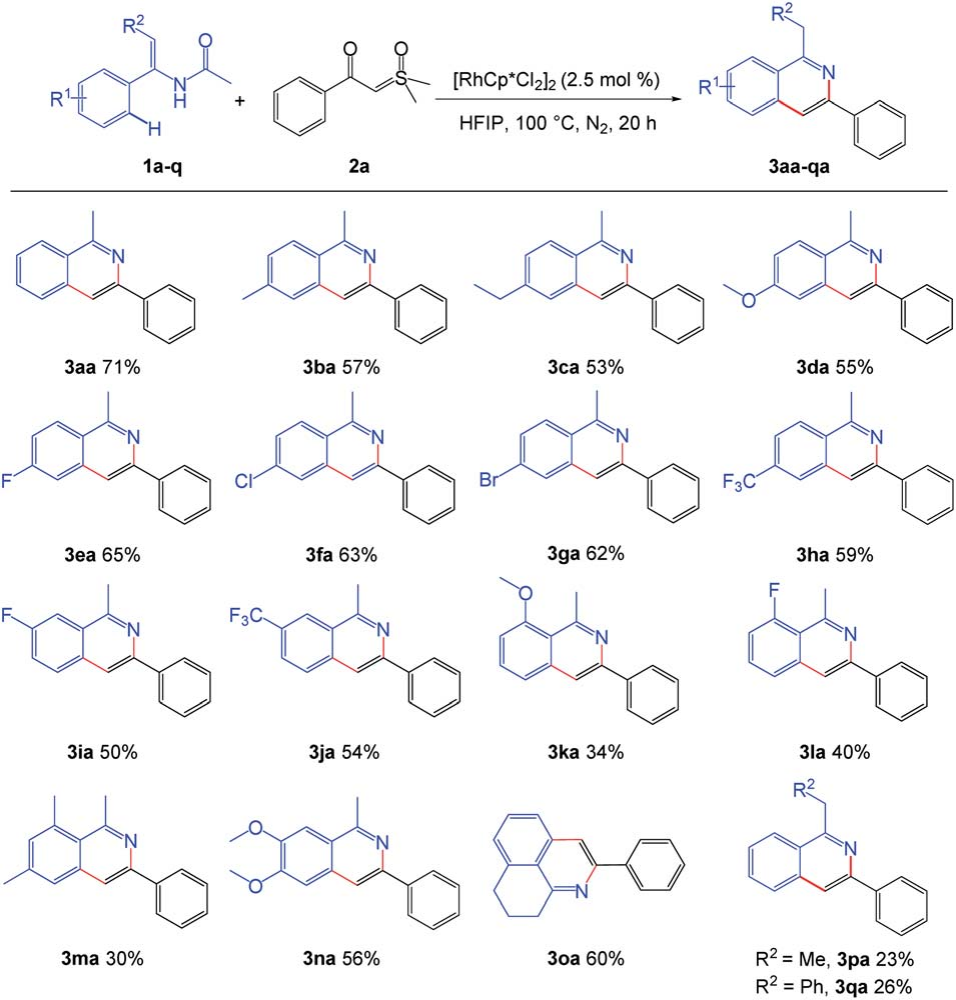
Scope of the enamides. ^a^ Reaction conditions: 1 (0.3 mmol), 2a (0.2 mmol), [RhCp*Cl_2_]_2_ (2.5 mol%), in HFIP (2.0 mL), at 100 °C, under N_2_, for 20 h.

With the optimized reaction conditions in hand, we then investigated the generality and scope of enamide. Diversified enamides bearing various aryl moieties substituted by electron-donating groups and electron-withdrawing groups reacted smoothly, affording the desired products in moderate to good yields. Among them, the enamide substrates with the *para*-position substituted by various electron-donating groups, such as –Me, –Et and –OMe could be smoothly converted into the desired products (Scheme 2, 3ba–3da). When the electron-withdrawing groups including –F, –Cl, –Br and –CF_3_ were introduced to the *para*-position of benzene ring of enamide, it was also tolerated to the standard reaction conditions, and gave the good yield (Scheme 2, 3ea–3ha). We further introduced –F and –CF_3_ groups into the 3-position of benzene ring of enamide 1a and good results were also obtained (Scheme 2, 3ia–3ja). However, the yield decreased markedly when *ortho* position of the benzene ring was substituted by –OMe or –F (Scheme 2, 3ka–3la). This may be due to steric effects. Interestingly, fused isoquinoline 3oa could also be obtained by this method in the yield of 60%. It was noteworthy that the methyl or phenyl substituted enamide on olefinic bond could also be converted into corresponding isoquinoline products 3pa or 3qa, which further expanding the scope of this protocol.

**Scheme 3:**
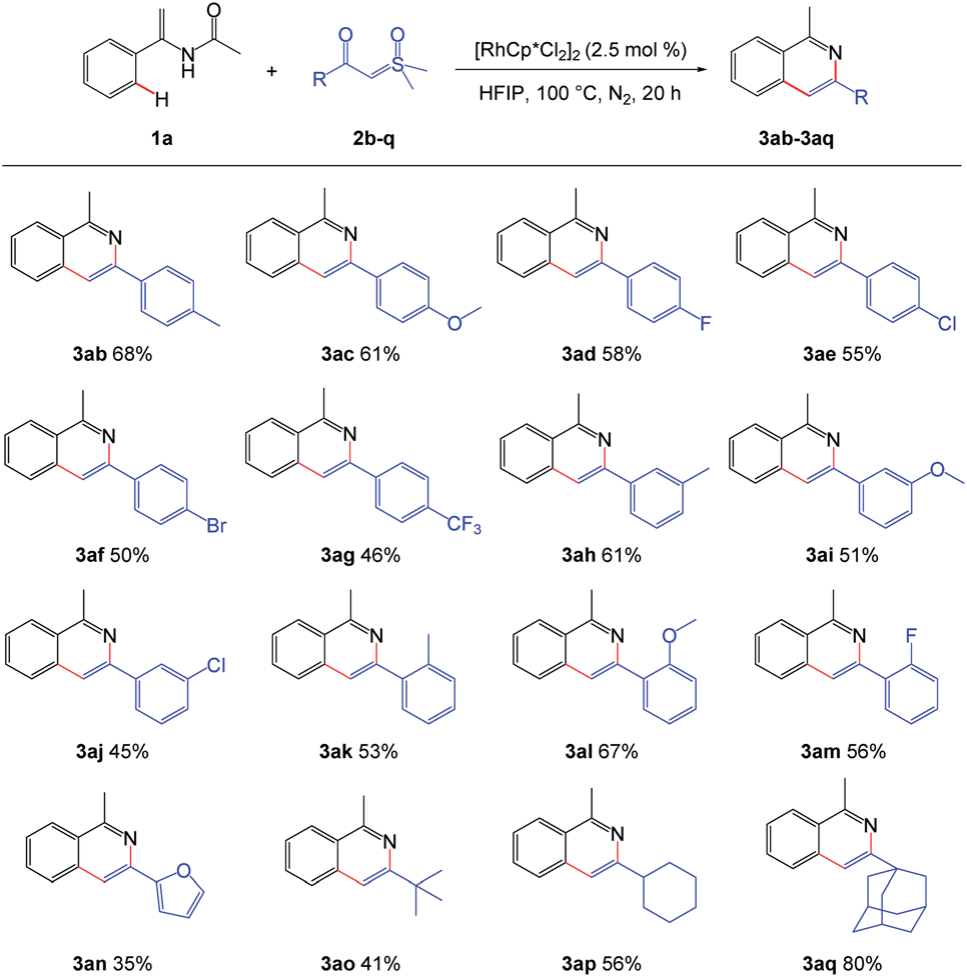
Scope of the sulfoxonium ylides. ^a^ Reaction conditions: 1a (0.3 mmol), 2 (0.2 mmol), [RhCp*Cl_2_]_2_ (2.5 mol%), in HFIP (2.0 mL), at 100 °C, under N_2_, for 20 h.

Next, the scope of sulfoxonium ylides was examined and the results are summarized in Scheme 3. Both the electron-donating and electron-withdrawing groups of the phenyl rings tolerated well, giving the desired isoquinolines in moderate to good yields (Scheme 3, 3ab–3am). When the phenyl group in sulfoxonium ylide 2a was switched to alkyl group, the reaction still performed smoothly to give the corresponding products (Scheme 3, 3ao–3aq). However, when the phenyl ring was replaced by a furan ring, 3an was obtained in a lower yield.

**Scheme 4:**
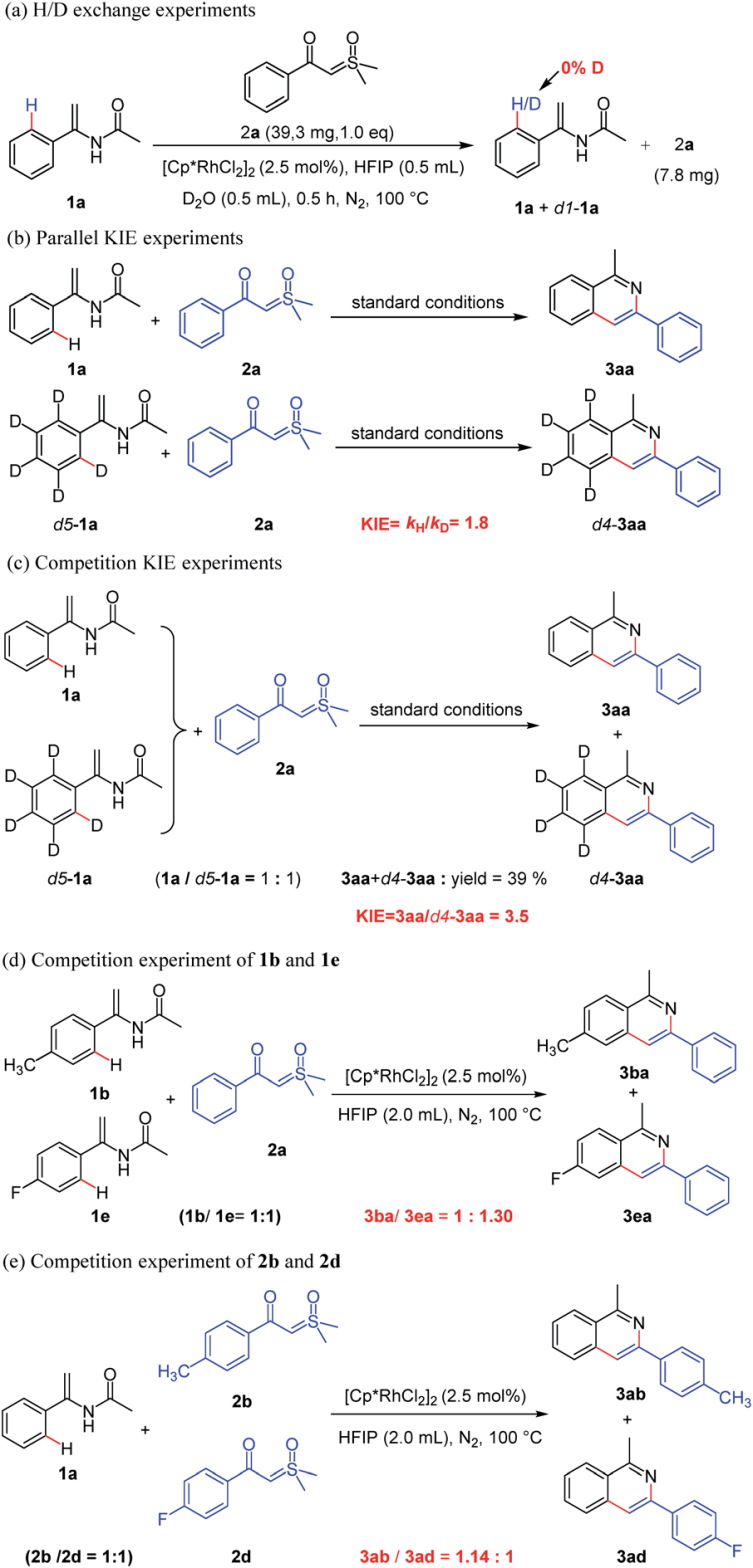
Mechanism study experiments.

With the established substrate scope of the products, we conducted a series of experiments to investigate the possible mechanism. Initially, when the reaction of 1a was performed with D_2_O and sulfoxonium ylide 2a under standard conditions for 0.5 h, 25% of 1a were recovered and no deuterium was found at the *ortho* position of the benzene ring, showing no H/D exchange (Scheme 4a). It should indicate that the C–H bond activation of enamide might follow an irreversible process. Then, two parallel independent reactions (Scheme 4b) and the one-pot deuterium competition reaction (Scheme 4c) of substrates 1a and *d5*-1a were carried out, giving *k*_H_/*k*_D_ values of 1.8 and 3.5 respectively. Both results indicate that the *ortho* C–H bond cleavage of enamide was likely involved in the turnover-limiting step. Moreover, the intermolecular competition experiment between electron-rich and electron-deficient enamides (1b*vis*1e) shows a ratio of products 3ba and 3ea of 1 : 1.30 based on the yields, suggesting that the aryl Csp^2^–H bond activation possibly proceeded through concerted metallation–deprotonation (CMD) process instead of electrophilic rhodiumization pathway (Scheme 4d). Finally, the competitive coupling-cyclization of *α*-(4-methylbenzoyl)-sulfoxonium ylide (2b) and *α*-(4-fluorobenzoyl) sulfoxonium ylide (2d) with enamide (1a) led to the yields of 3ab and 3ad with a ratio of 1.14 : 1 based on the yields (Scheme 4e), indicating that electron-rich sulfoxonium ylide more easily forms a rhodium-carbene than the electron-deficient sulfoxonium ylide, which facilitates the formation of isoquinoline.

**Scheme 5:**
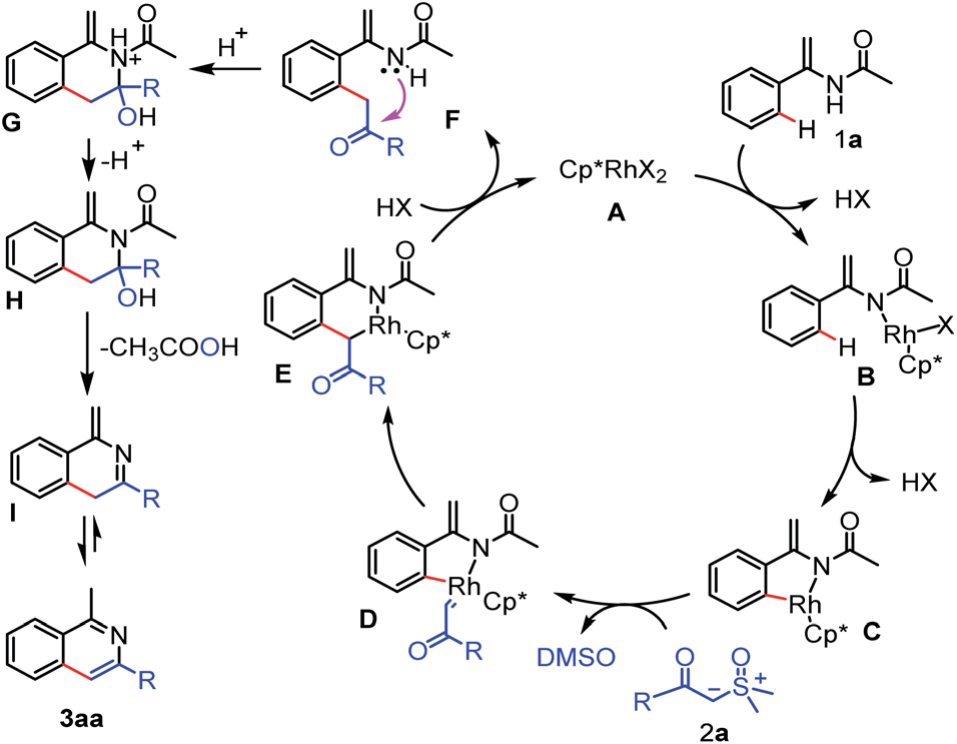
Proposed reaction pathway.

On the basis of the above experimental results and precedent literatures,^14*d*,15*d*,18^ a plausible reaction mechanism was proposed as shown in Scheme 5. The process begins with coordination of nitrogen atom of enamide 1a to the rhodium atom of A and subsequent Csp^2^–H activation *via* the concerted metalation-deprotonation (CMD) afford the key five-membered rhodacycle C, which is trapped by the sulfoxonium ylide 2a to form the rhodium-carbene D through the elimination of DMSO. Next, migratory insertion of carbene species into the Rh–C(sp^2^) bond in the intermediate D provides a six-membered rhodacycle intermediate E. Finally, protonolysis of E produces the acylmethylated intermediate F, which undergoes successive addition, elimination and aromatization steps under the acidic conditions to afford the target product 3aa.

## Conclusions

In summary, we have disclosed a novel strategy for the synthesis of isoquinolines *via* rhodium(iii)-catalyzed C–H activation and annulation. The useful building blocks of enamides and sulfoxonium ylides are applied, and a range of substituted isoquinolines are prepared under mild reaction conditions. This versatile method needs not any additives such as silver salts and mesitylenic acid. Further investigation to expand the applications of enamides is underway in our laboratory.

## Conflicts of interest

There are no conflicts to declare.

## Supplementary Material

RA-011-D1RA01063H-s001
