# Supplementary material for: Rhodium(iii)-catalyzed annulation of enamides with sulfoxonium ylides toward isoquinolines
Source: RSC Adv. 2021 Mar 19;11(19):11490–4. doi: 10.1039/d1ra01063h (PMC8698508; doi:10.1039/d1ra01063h)
Supplement: RA-011-D1RA01063H-s001 [file RA-011-D1RA01063H-s001.pdf]

Electronic Supplementary Material (ESI) for RSC Advances.
This journal is © The Royal Society of Chemistry 2021

Rhodium(III)-Catalyzed Annulation of Enamides with Sulfoxonium Ylides toward Isoquinolines

Chao Hong,[a] Shuling Yu,[a] Zhanxiang Liu,*,[a] and Yuhong Zhang*,[a,b]

[a] Department of Chemistry, Zhejiang University, Hangzhou 310027, People's Republic of China

[b] State Key Laboratory of Applied Organic Chemistry, Lanzhou University, Lanzhou 730000, People's Republic of China.

E-mail: liuzhanx@zju.edu.cn; yhzhang@zju.edu.cn

Supporting Information

Table of Contents

1. General Information S2
2. Optimization of the Reaction Conditions.. S2
3. Synthesis of Substrates S3
   3.1 Synthesis of Enamides..S3
   3.2 Synthesis of Sulfoxonium Ylides..S4
4. General Procedure for the Synthesis of **3** (**3aa** as an example) S4
5. Mechanism Research S5
   5.1 H/D Exchange Experiments S5
   5.2 Parallel KIE Experiments S6
   5.3 Competition KIE Experiments S6
   5.4 Competition Experiment of **1b** and **1e**..S7
   5.5 Competition Experiment of **2b** and **2d**..S8
6. Characterization Data of Products S10
7. Copies of NMR Spectra of Products S23

1. General Information

Unless otherwise indicated, all reagents and solvents were purchased from commercial sources and used without further purification. All reactions under standard condition were monitored by thin-layer chromatography (TLC) on silica gel F254 plates. Column chromatography was performed on silica gel (300-400 meshes) with ethyl acetate (EA)/petroleum ether (PE). ^{1}H NMR, ^{13}C NMR and ^{19}F NMR were recorded at 400 MHz, 100 MHz and 376 MHz respectively in $CDCl_3$ solution on Bruker AVANCE III 400 MHz instruments, using TMS as internal standard. The following abbreviations were used to describe peak patterns where appropriate: singlet (s), doublet (d), triplet (t), quartet (q), multiplet (m), doublet of doublet (dd), broad resonances (br). Highresolution mass spectra (HRMS) data of the products were collected using Waters GCT Premier instruments. Melting points were measured on a micro melting apparatus and uncorrected. Starting materials enamides[1] and sulfoxonium ylides[2] were prepared by following the literature methods.

2. Optimization of the Reaction Conditions

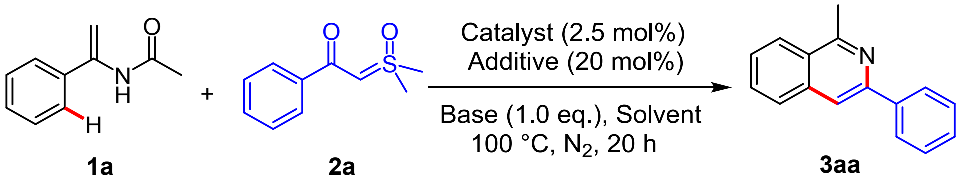

Table 1. Screening of Reaction Conditions.[a]

entry	catalyst（mol%)	Additive (20 mol%)	Base(1.0 eq)	solvent	Yield(%)[b]
1	$[RhCp^*Cl_2]_2$	$AgSbF_6$	NaOAc	THF	N.R.
2	$[RhCp^*Cl_2]_2$	$AgSbF_6$	NaOAc	DMF	N.R.
3	$[RhCp^*Cl_2]_2$	$AgSbF_6$	NaOAc	EtOH	N.R.
4	$[RhCp^*Cl_2]_2$	$AgSbF_6$	NaOAc	DCE	N.R.
5	$[RhCp^*Cl_2]_2$	$AgSbF_6$	NaOAc	HFIP	25
6	$[RhCp^*Cl_2]_2$	$AgSbF_6$	LiOAc	HFIP	19
7	$[RhCp^*Cl_2]_2$	$AgSbF_6$	KOAc	HFIP	18
8	$[RhCp^*Cl_2]_2$	$AgSbF_6$	CsOAc	HFIP	20
9	$[RhCp^*Cl_2]_2$	$AgSbF_6$	$Cu(OAc)_2$	HFIP	5
10	$[RhCp^*Cl_2]_2$	AgOAc	NaOAc	HFIP	23
11	$[RhCp^*Cl_2]_2$	AgF	NaOAc	HFIP	22
12	$[RhCp^*Cl_2]_2$	$AgBF_4$	NaOAc	HFIP	16

13	$[RhCp^*Cl_2]_2$	-	NaOAc	HFIP	46
14	$[RhCp^*Cl_2]_2$	-	LiOAc	HFIP	30
15	$[RhCp^*Cl_2]_2$	-	KOAc	HFIP	28
16	$[RhCp^*Cl_2]_2$	-	CsOAc	HFIP	29
17	$[RhCp^*Cl_2]_2$	-	$Cu(OAc)_2$	HFIP	12
**18**	$\mathbf{[RhCp^*Cl_2]_2}$	-	-	**HFIP**	**71**
19	$[Rh(cod)Cl]_2$	-	-	HFIP	N.R.
20	$Rh(PPh_3)_3Cl$	-	-	HFIP	N.R.
21	$RhCl_3$	-	-	HFIP	N.R.
22	[Ru(*p*-cymene)$Cl_2]_2$	-	-	HFIP	N.R.
23	-	-	-	HFIP	N.R.
24	$[RhCp^*Cl_2]_2$	-	-	HFIP [c]	38
25[d]	$[RhCp^*Cl_2]_2$	-	-	HFIP	53
26[e]	$[RhCp^*Cl_2]_2$	-	-	HFIP	65

a) Reaction conditions: **1a** (0.3 mmol), **2a** (0.2 mmol), $[RhCp^*Cl_2]_2$ (2.5 mol%), additive (20 mol%), base (1.0 equiv), solvent (2.0 mL), 100 ºC, under N_2, for 20 h, N.R. = No Reaction.
b) Isolated yields.
c) HFIP (1mL).
d) 80 ºC.
e) 120 ºC.

3. Synthesis of Substrates

3.1 Synthesis of Enamides (1a as an example)

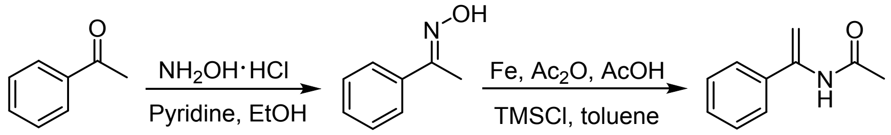

1a

Acetophenone (11.7 mL, 100 mmol) and hydroxylamine hydrochloride salt (14.8 g, 213 mmol) were dissolved in ethanol (150 mL) and pyridine (15 mL). After refluxing for 5 h, the solvent was evaporated and water (150 mL) was added after the solution was cooled by an ice bath. The precipitate was filtered and washed with ice/water (50 mL) before being dissolved in ethyl acetate (150 mL), dried over anhydrous Na_2SO_4, and the solvent evaporated under reduced pressure. No further purification was required for the next step.

To a solution of the resulting oxime (12.4 g, 92 mmol) in toluene (135 mL), Ac_2O (26.1 mL, 276 mmol), AcOH (10.3 mL, 276 mmol), Fe (10.8 g, 193 mmol), and a few drops of TMSCl were added. After stirring at 70 ℃ for 5 hours, the mixture was

cooled to room temperature and filtered over Celite ¾ , washed with toluene (2×30 mL) and NaOH (2 M, 2×135 mL), dried over anhydrous Na_2SO_4 , and the solvent evaporated under reduced pressure. Then the crude product was purified by silica gel chromatography using PE/EA to afford the corresponding enamide **1a**.

3.2 Synthesis of Sulfoxonium Ylides (2a as an example)

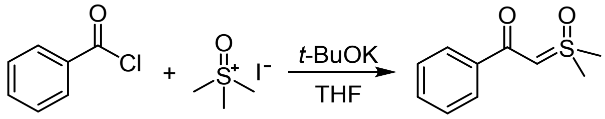

2a

To a flame-dried 50 mL round bottom flask, adding potassium tert-butoxide (3.0 g, 27.2 mmol) and dry THF (30 mL) at room temperature. After stirring for 10 mins, trimethylsulfoxonium iodide (5.0 g, 20.6 mmol) was added and the resulting mixture was stirred at reflux for 2 h. Subsequently, the reaction was cooled to 0 °C and acyl chlorides (7 mmol) was added dropwise to the reaction mixture. The reaction was allowed to room temperature and stirred for 3h. Upon completion, the solvent was evaporated under vacuum and the resulting slurry was exacted with water and ethyl acetate. The combined organic layers were washed with saturated brine and dried over anhydrous Na_2SO_4. After the solvent was evaporated, the crude product was purified by silica gel chromatography using EtOAc/MeOH (95 : 5) to afford the corresponding sulfoxonium ylide **2a**.

4. General Procedure for the Synthesis of 3 (3aa as an example)

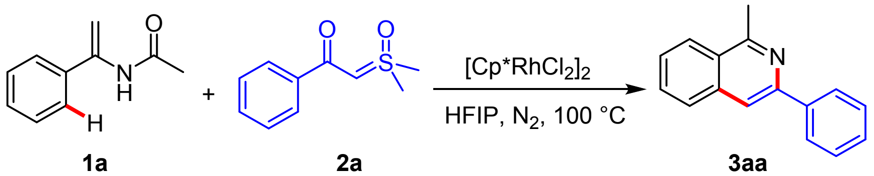

1a **2a** **3aa**

A 50 mL sealed tube with a magnetic stir bar was charged with $[RhCp^*Cl_2]_2$ (3mg, 0.005 mmol), enamide **1a** (48.3 mg, 0.3 mmol), sulfoxonium ylide **2a** (39.3 mg, 0.2 mmol) and HFIP (2 mL). The reaction mixture was stirred under N_2 condition at 100 °C for 20 h. After cooling down, the solvent was removed under reduced pressure and the residue was purified by silica gel chromatography using PE/EA to afford the target product **3aa**.

5. Mechanism Research

5.1 H/D Exchange Experiments

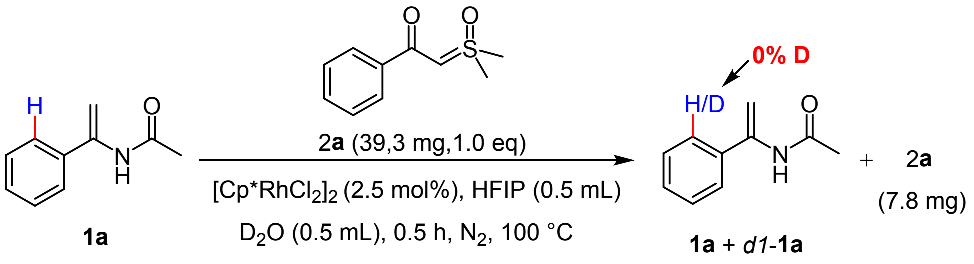

To a dried sealed tube were sequentially added enamide **1a** (48.4 mg, 0.3 mmol, 1.0 equiv), sulfoxonium ylide **2a** (39.3 mg, 0.2 mmol) and [(Cp*RhCl$_2$]$_2$ (3 mg, 0.005 mmol, 2.5 mol%) in open air atmosphere. After being evacuated and backfilled with nitrogen three times, dry HFIP (0.5 mL) and D_2O (0.5 mL) were added. Then, the mixture was stirred at 100 ℃ in a pre-heated oil bath for 0.5 h. Then, the mixture was cooled to room temperature and extracted with ethyl acetate (10 mL × 2). After concentration in vacuo, the crude residual was directly purified by flash column chromatography on silica gel (eluent: PE/EA = 5/1) to recover enamide **1a** as a white solid (12.1 mg, 25% yield, 0% deuterium) and sulfoxonium ylide **2a** as a light yellow solid (7.8 mg, 20% yield).

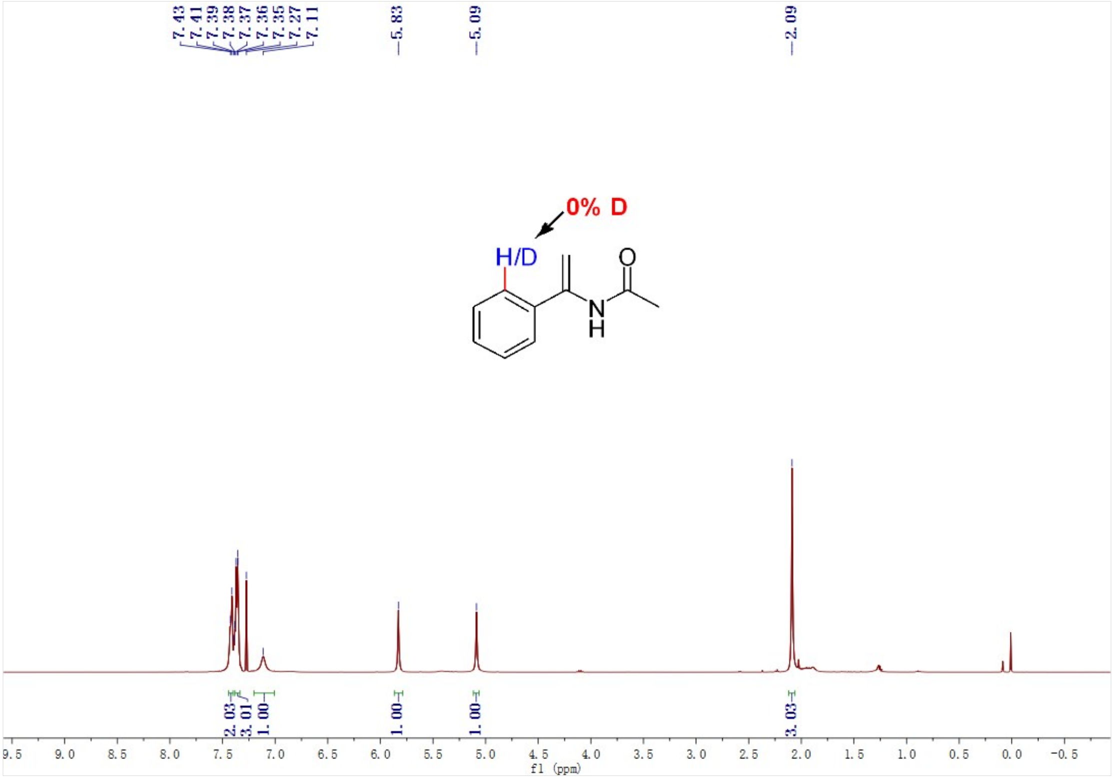

5.2 Parallel KIE Experiments

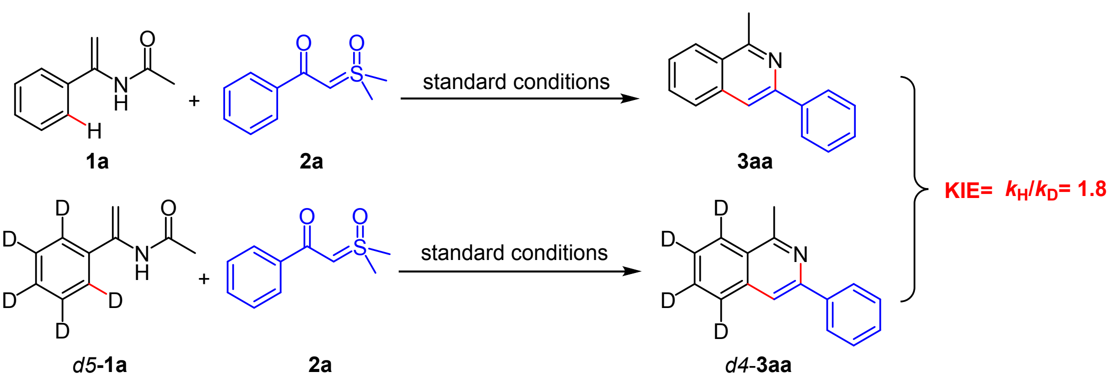

A mixture of sulfoxonium ylide **2a** (39.3 mg, 0.2 mmol, 1.0 equiv) and $[(Cp^*RhCl_2]_2$ (3.0 mg, 0.005 mmol, 2.5 mol%) were weighted in each of two sealed tubes equipped with a stir bar. The parallel tubes were then separately introduced **1a** (48.3 mg, 0.3 mmol, 1.5 equiv) and *d5*-**1a** (49.9 mg, 0.3 mmol, 1.5 equiv). Dry HFIP (2.0 mL) was added and the mixture was stirred at 100 °C in a pre-heated oil bath for 20 h under N_2 atmosphere. Then, the mixture was cooled to room temperature and concentrated in vacuo and the resulting residue was purified by flash column chromatography on silica gel with PE/EA to give the desired product **3aa** in 46% yield (20.0 mg, 0.0866 mmol) or *d4*-**3aa** in 29% yield (13.0 mg, 0.0493 mmol) (eluent: PE/EA = 60/1). The KIE value was calculated to be k_H/k_D = 1.8 according to isolated yields of **3aa** and *d4*-**3aa**.

5.3 Competition KIE Experiments

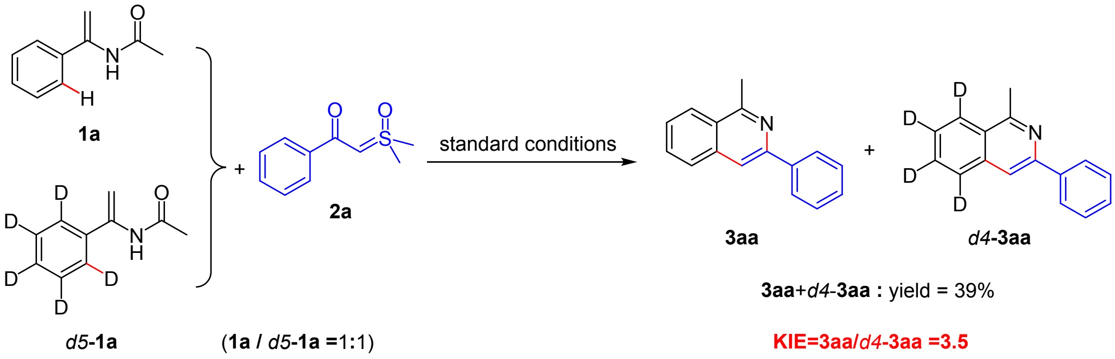

A mixture of enamide **1a** (48.3 mg, 0.3 mmol, 1.5 equiv), *d5*-**1a** (49.9 mg, 0.3

mmol, 1.5 equiv), sulfoxonium ylide **2a** (39.3 mg, 0.2 mmol, 1.0 equiv) and [(Cp*RhCl$_2$]$_2$ (3.0 mg, 0.005 mmol, 2.5 mol%) were weighted in a sealed tube equipped with a stir bar. Dry HFIP (2.0 mL) was added and the mixture was stirred at 100 °C in a pre-heated oil bath for 20 h under N_2 atmosphere. Then, the mixture was cooled to room temperature and concentrated in vacuo and the resulting residue was purified by flash column chromatography on silica gel with PE/EA (eluent: PE/EA = 60/1). The KIE value was determined to be k_H /k_D = 3.5 on the basis of ^{1}H NMR analysis.

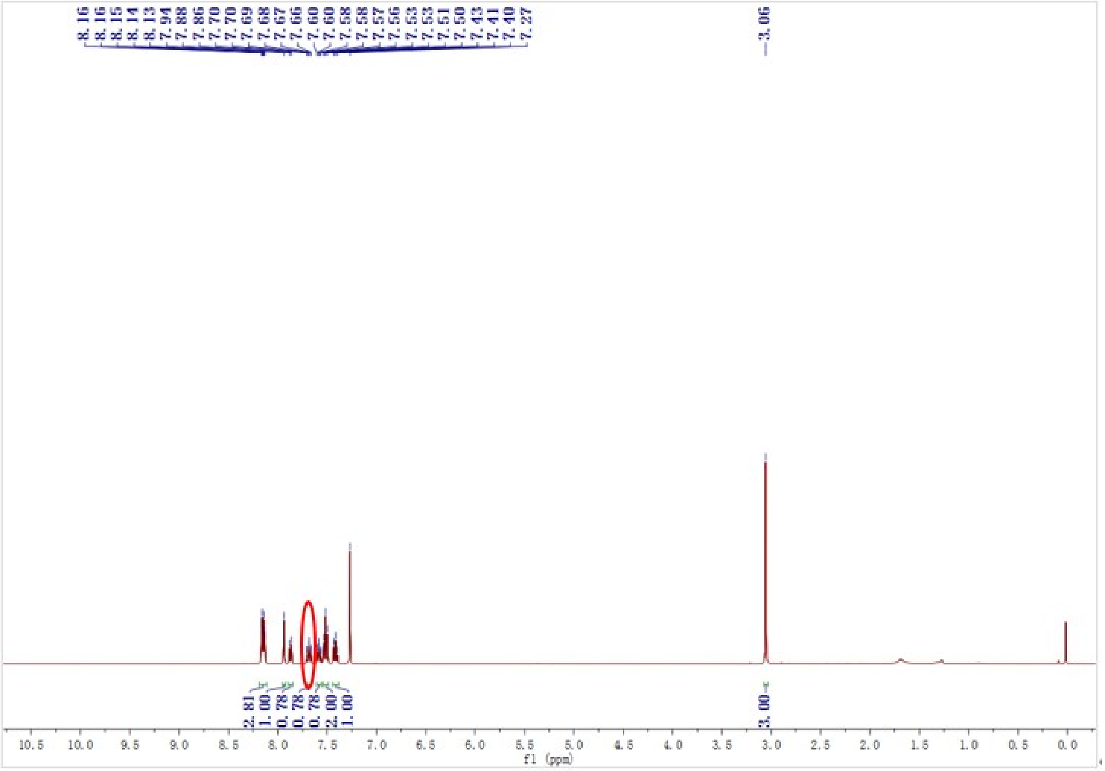

KIE = 0.78/0.22 = 3.5

5.4 Competition Experiment of 1b and 1e

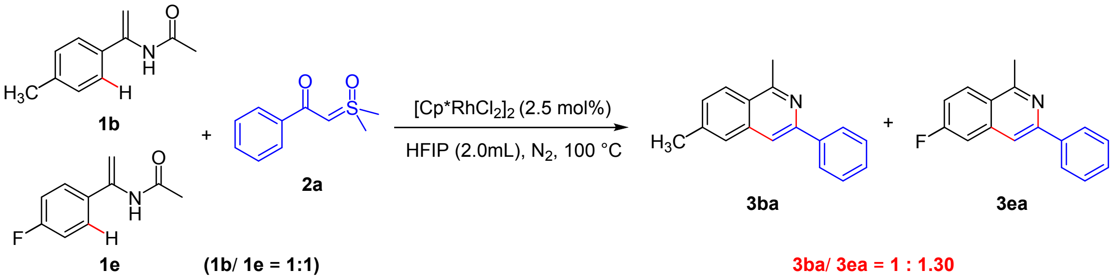

A mixture of 4-methylenamide **1b** (52.6 mg, 0.3 mmol, 1.5 equiv), 4-fluoroenamide **1e** (68.8 mg, 0.3 mmol, 1.5 equiv), sulfoxonium ylide **2a** (39.3 mg, 0.2 mmol, 1.0 equiv) and $[(Cp^*RhCl_2]_2$ (3.0 mg, 0.005 mmol, 2.5 mol%) were weighted in a sealed tube equipped with a stir bar. Dry HFIP (2.0 mL) was added and the mixture was stirred at 100 °C in a pre-heated oil bath for 20 h under N_2 atmosphere. Then, the mixture was cooled to room temperature and concentrated in vacuo and the resulting residue was purified by flash column chromatography on silica gel with PE/EA to give a mixture of products **3ba** and **3ea** at a ratio of 1:1.30 on the basis of ^1H NMR analysis.

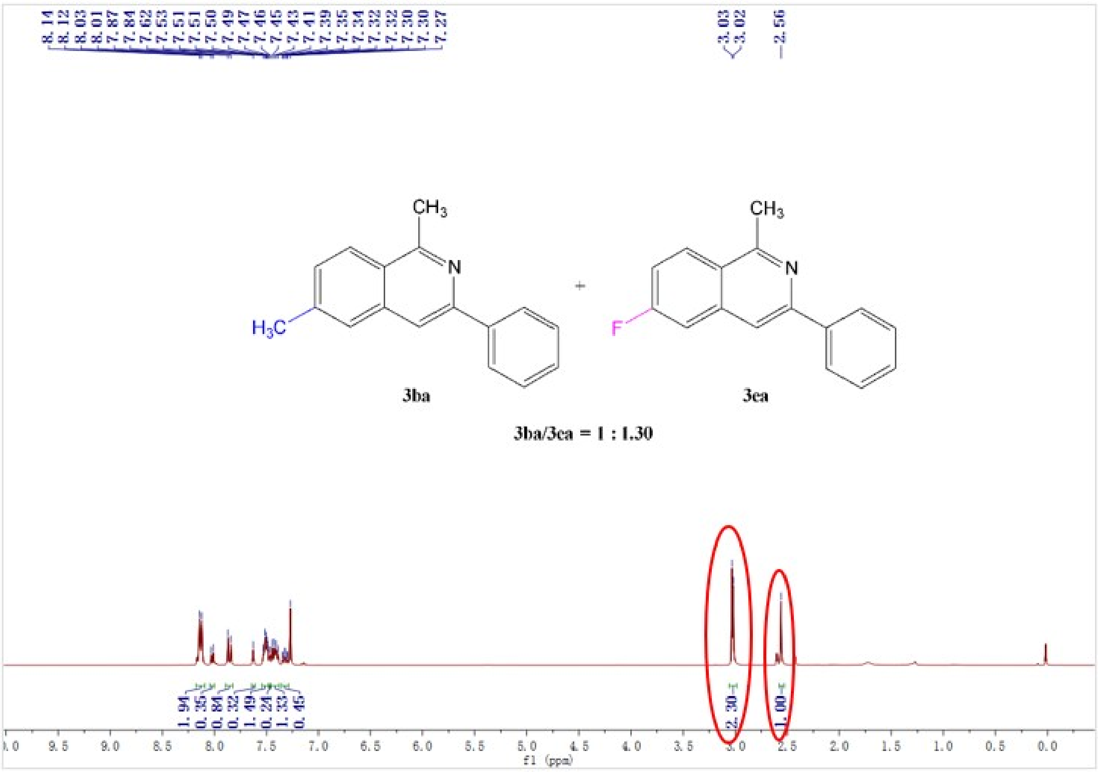

5.5 Competition Experiment of 2b and 2d

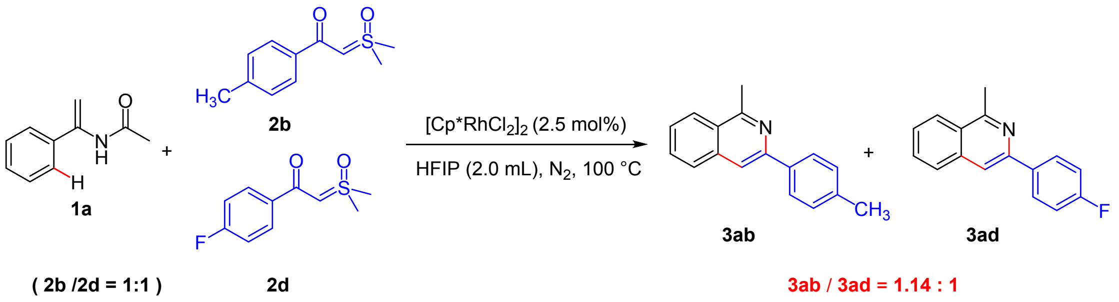

A mixture of enamide **1a** (48.4 mg, 0.3 mmol, 1.5 equiv), *α*-(4-methylbenzoyl)-

sulfoxonium ylide **2b** (42.1 mg, 0.2 mmol, 1.0 equiv), *α*-(4-fluorobenzoyl)-sulfoxonium ylide **2d** (42.9 mg, 0.2 mmol, 1.0 equiv) and $[(Cp^*RhCl_2]_2$ (3.0 mg, 0.005 mmol, 2.5 mol%) were weighted in a sealed tube equipped with a stir bar. Dry HFIP (2.0 mL) was added and the mixture was stirred at 100 ℃ in a pre-heated oil bath for 20 h under N_2 atmosphere. Then, the mixture was cooled to room temperature and concentrated in vacuo and the resulting residue was purified by flash column chromatography on silica gel with PE/EA to give a mixture of products **3ab** and **3ad** at a ratio of 1.14 : 1 on the basis of ^{1}H NMR analysis.

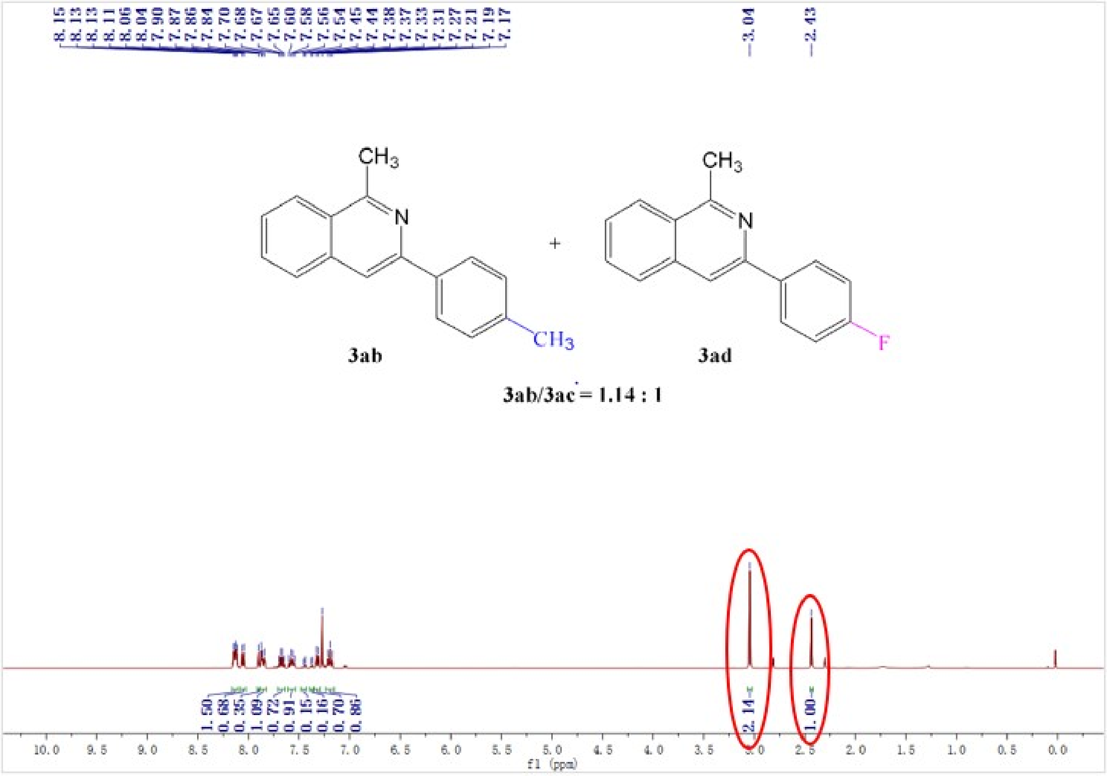

References

1. M. Van den Berg, R. M. Haak, A. J. Minnaard, A. H. M. de Vries, J. G. de Vries, B. L. Feringa, *Adv. Synth. Catal.* **2002**, *344*, 1003-1007.
2. M. Barday, C. Janot, N. R. Halcovitch, J. Muir, C. Aïssa, *Angew. Chem. Int. Ed.* **2017**, *56*, 13117-13121.

6. Characterization Data of Products

1-methyl-3-phenylisoquinoline (3aa)

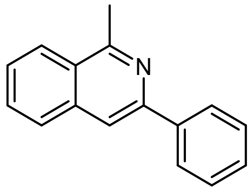

The title compound was prepared according to the general procedure and purified by flash column chromatography to give the corresponding product as a yellow oil, 31.1 mg, 71% yield.

^{1}H NMR (400 MHz, $CDCl_3$) δ 8.15 (d, J = 7.3 Hz, 3H), 7.94 (s, 1H), 7.87 (d, J = 8.2 Hz, 1H), 7.68 (t, J = 7.4 Hz, 1H), 7.58 (t, J = 7.6 Hz, 1H), 7.51 (t, J = 7.6 Hz, 2H), 7.41 (t, J = 7.3 Hz, 1H), 3.06 (s, 3H);

^{13}C NMR (101 MHz, $CDCl_3$) δ 158.60, 150.13, 139.88, 136.78, 130.03, 128.74, 128.31, 127.65, 127.00, 126.79, 126.61, 125.66, 115.25, 22.71;

HRMS (EI-TOF) (M^{+}) calculated for $C_{16}H_{13}N$ 219.1048, found 219.1048.

1,6-dimethyl-3-phenylisoquinoline (3ba)

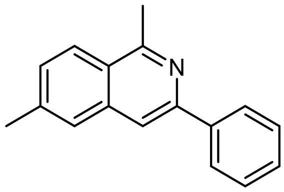

The title compound was prepared according to the general procedure and purified by flash column chromatography to give the corresponding product as a yellow oil, 26.6 mg, 57% yield.

^{1}H NMR (400 MHz, $CDCl_3$) δ 8.17–8.10 (m, 2H), 8.02 (d, J = 8.5 Hz, 1H), 7.84 (s, 1H), 7.63 (s, 1H), 7.51 (t, J = 7.6 Hz, 2H), 7.41 (t, J = 7.4 Hz, 2H), 3.02 (s, 3H), 2.56 (s, 3H);

^{13}C NMR (100 MHz, $CDCl_3$) δ 158.42, 150.28, 140.42, 140.20, 137.26, 129.20, 128.89, 128.40, 127.16, 126.75, 125.68, 125.18, 115.07, 22.80, 22.06;

HRMS (EI-TOF) (M^{+}) calculated for $C_{17}H_{15}N$ 233.1204, found 233.1205.

6-ethyl-1-methyl-3-phenylisoquinoline (3ca)

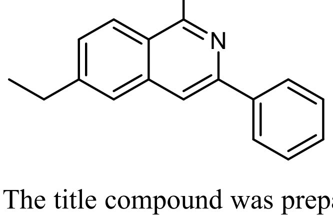

The title compound was prepared according to the general procedure and purified by flash column chromatography to give the corresponding product as a yellow oil, 26.2 mg, 53% yield.

^{1}H NMR (400 MHz, $CDCl_3$) δ 8.14 (d, J = 7.5 Hz, 2H), 8.05 (d, J = 8.6 Hz, 1H), 7.88 (s, 1H), 7.65 (s, 1H), 7.51 (t, J = 7.6 Hz, 2H), 7.48–7.35 (m, 2H), 3.03 (s, 3H), 2.86 (q, J = 7.6 Hz, 2H), 1.37 (t, J = 7.6 Hz, 3H);
^{13}C NMR (100 MHz, $CDCl_3$) δ 158.42, 150.27, 146.60, 140.22, 137.36, 128.89, 128.40, 128.21, 127.17, 125.82, 125.45, 125.41, 115.28, 29.31, 22.81, 15.41;
HRMS (EI-TOF) (M $^+$) calculated for $C_{18}H_{17}N$ 247.1361, found 247.1362.

6-methoxy-1-methyl-3-phenylisoquinoline (3da)

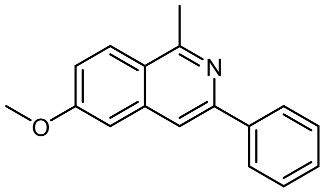

The title compound was prepared according to the general procedure and purified by flash column chromatography to give the corresponding product as a yellow oil, 27.4 mg, 55% yield.
^{1}H NMR (400 MHz, $CDCl_3$) δ 8.18–8.08 (m, 2H), 8.02 (d, J = 9.1 Hz, 1H), 7.83 (s, 1H), 7.51 (t, J = 7.6 Hz, 2H), 7.41 (t, J = 7.3 Hz, 1H), 7.19 (dd, J = 9.1, 2.5 Hz, 1H), 7.12 (d, J = 2.5 Hz, 1H), 3.96 (s, 3H), 2.99 (s, 3H);
^{13}C NMR (100 MHz, $CDCl_3$) δ 160.80, 158.11, 150.88, 140.19, 138.99, 128.87, 128.46, 127.64, 127.20, 122.45, 119.59, 115.02, 105.36, 55.62, 22.76;
HRMS (EI-TOF) (M $^+$) calculated for $C_{17}H_{15}NO$ 249.1154, found 249.1152.

6-fluoro-1-methyl-3-phenylisoquinoline (3ea)

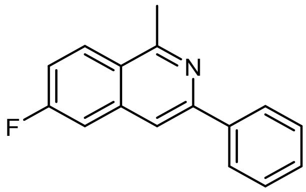

The title compound was prepared according to the general procedure and purified by flash column chromatography to give the corresponding product as a yellow oil, 30.8 mg, 65% yield.
^{1}H NMR (400 MHz, $CDCl_3$) δ 8.14 (dd, J = 8.1, 6.6 Hz, 3H), 7.86 (s, 1H), 7.51 (t, J = 7.5 Hz, 2H), 7.44 (dt, J = 11.0, 4.9 Hz, 2H), 7.32 (dt, J = 8.8, 2.5 Hz, 1H), 3.03 (s, 3H);
^{13}C NMR (101 MHz, $CDCl_3$) δ 164.57, 162.07, 158.69, 151.26, 139.64, 138.66 (d, J = 10.2 Hz), 128.96, 128.83 (d, J = 5.3 Hz), 127.25, 123.99, 117.13 (d, J = 25.1 Hz), 115.05 (d, J = 5.2 Hz), 110.92 (d, J = 20.3 Hz), 22.98; ^{19}F NMR (376 MHz, $CDCl_3$) δ -108.61;
HRMS (EI-TOF) (M $^+$) calculated for $C_{16}H_{12}NF$ 237.0954, found 237.0953.

6-chloro-1-methyl-3-phenylisoquinoline (3fa)

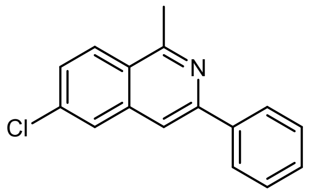

The title compound was prepared according to the general procedure and purified by flash column chromatography to give the corresponding product as a yellow oil, 31.9 mg, 63% yield.

^{1}H NMR (400 MHz, $CDCl_3$) δ 8.12 (d, J = 7.2 Hz, 2H), 8.05 (d, J = 8.9 Hz, 1H), 7.84–7.80 (m, 2H), 7.51 (m, 3H), 7.43 (dt, J = 9.2, 4.2 Hz, 1H), 3.02 (s, 3H);

^{13}C NMR (100 MHz, $CDCl_3$) δ 158.82, 151.34, 139.55, 137.87, 136.36, 128.98, 128.85, 127.83, 127.61, 127.22, 126.47, 124.96, 114.46, 22.88;

HRMS (EI-TOF) (M^+) calculated for $C_{16}H_{12}NCl$ 253.0658, found 253.0656.

6-bromo-1-methyl-3-phenylisoquinoline (3ga)

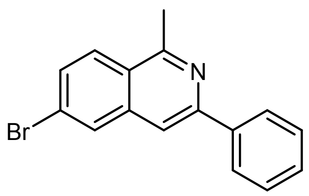

The title compound was prepared according to the general procedure and purified by flash column chromatography to give the corresponding product as a yellow oil, 36.8 mg, 62% yield.

^{1}H NMR (400 MHz, $CDCl_3$) δ 8.15–8.09 (m, 2H), 8.02 (d, J = 1.8 Hz, 1H), 7.98 (d, J = 8.9 Hz, 1H), 7.81 (s, 1H), 7.63 (dd, J = 8.9, 1.9 Hz, 1H), 7.51 (t, J = 7.5 Hz, 2H), 7.43 (t, J = 7.3 Hz, 1H), 3.02 (s, 3H);

^{13}C NMR (100 MHz, $CDCl_3$) δ 158.94, 151.33, 139.55, 138.23, 130.37, 129.84, 128.99, 128.87, 127.59, 127.23, 125.16, 124.93, 114.29, 22.84;

HRMS (EI-TOF) (M^+) calculated for $C_{16}H_{12}NBr$ 297.0153, found 297.0153.

1-methyl-3-phenyl-6-(trifluoromethyl)isoquinoline (3ha)

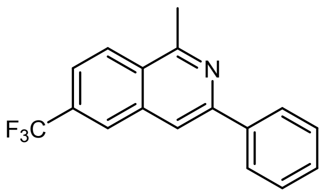

The title compound was prepared according to the general procedure and purified by flash column chromatography to give the corresponding product as a yellow oil, 33.9 mg, 59% yield.

^{1}H NMR (400 MHz, $CDCl_3$) δ 8.24 (d, J = 8.7 Hz, 1H), 8.16 (dd, J = 5.1, 3.3 Hz, 3H), 7.99 (s, 1H), 7.73 (dd, J = 8.7, 1.6 Hz, 1H), 7.56–7.50 (m, 2H), 7.48–7.42 (m, 1H), 3.08 (s, 3H);

^{13}C NMR (100 MHz, $CDCl_3$) δ 159.04, 151.57, 139.29, 136.20, 132.04, 131.71, 129.06, 127.55, 127.24, 127.12, 125.49 (q, J = 4.2 Hz), 122.69, 122.56 (q, J = 4.0 Hz), 115.62, 22.94;

^{19}F NMR (376 MHz, $CDCl_3$) δ -62.94;

HRMS (EI-TOF) (M^+) calculated for $C_{17}H_{12}NF_3$ 287.0922, found 287.0922.

7-fluoro-1-methyl-3-phenylisoquinoline (3ia)

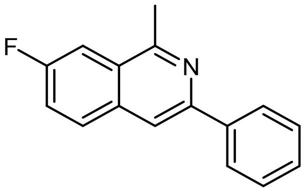

The title compound was prepared according to the general procedure and purified by flash column chromatography to give the corresponding product as a yellow oil, 23.7 mg, 50% yield.

^{1}H NMR (400 MHz, $CDCl_3$) δ 8.17 (dd, J = 5.3, 3.1 Hz, 3H), 7.92 (d, J = 8.4 Hz, 1H), 7.55–7.49 (m, 3H), 7.43 (t, J = 6.7 Hz, 1H), 7.35 (dd, J = 9.9, 7.8 Hz, 1H), 3.06 (s, 3H);

^{13}C NMR (100 MHz, $CDCl_3$) δ 159.89, 158.60 (d, J = 3.0 Hz), 157.37, 150.61, 139.70, 128.99, 128.83, 127.27, 127.10, 126.50 (d, J = 7.8 Hz), 121.68 (d, J = 4.4 Hz), 113.68 (d, J = 19.5 Hz), 107.80 (d, J = 4.5 Hz), 23.23;

^{19}F NMR (376 MHz, $CDCl_3$) δ -122.66;

HRMS (EI-TOF) (M^+) calculated for $C_{16}H_{12}NF$ 237.0954, found 237.0955.

1-methyl-3-phenyl-7-(trifluoromethyl)isoquinoline (3ja)

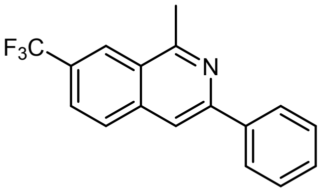

The title compound was prepared according to the general procedure and purified by flash column chromatography to give the corresponding product as a yellow oil, 31.0 mg, 54% yield.

^{1}H NMR (400 MHz, $CDCl_3$) δ 8.41 (s, 1H), 8.19–8.12 (m, 2H), 7.96 (t, J = 4.2 Hz, 2H), 7.83 (dd, J = 8.7, 1.5 Hz, 1H), 7.56–7.49 (m, 2H), 7.48–7.42 (m, 1H), 3.09 (s, 3H);

^{13}C NMR (100 MHz, $CDCl_3$) δ 159.79, 152.22, 139.26, 138.41, 129.14, 129.02 (d, J = 5.3 Hz), 127.31, 125.81 (q, J = 2.8 Hz), 125.58, 123.71 (q, J = 4.5 Hz), 114.90, 22.84;

^{19}F NMR (376 MHz, $CDCl_3$) δ -62.59;

HRMS (EI-TOF) (M^+) calculated for $C_{17}H_{12}NF_3$ 287.0922, found 287.0924.

8-methoxy-1-methyl-3-phenylisoquinoline (3ka)

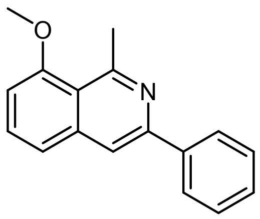

The title compound was prepared according to the general procedure and purified by flash column chromatography to give the corresponding product as a yellow oil, 16.9 mg, 34% yield.

^{1}H NMR (400 MHz, $CDCl_3$) δ 8.15 (dd, J = 5.2, 3.3 Hz, 2H), 7.84 (s, 1H), 7.57–7.47 (m, 3H), 7.45–7.35 (m, 2H), 6.88 (d, J = 7.7 Hz, 1H), 4.00 (s, 3H), 3.20 (s, 3H);

^{13}C NMR (100 MHz, $CDCl_3$) δ 158.65, 158.36, 150.03, 139.86, 139.78, 130.47, 128.87, 128.53, 127.12, 120.07, 119.56, 115.02, 106.27, 55.65, 29.31;

HRMS (EI-TOF) (M^+) calculated for $C_{17}H_{15}NO$ 249.1154, found 249.1156.

8-fluoro-1-methyl-3-phenylisoquinoline (3la)

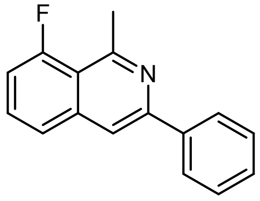

The title compound was prepared according to the general procedure and purified by flash column chromatography to give the corresponding product as a yellow oil, 18.9 mg, 40% yield.

^{1}H NMR (400 MHz, $CDCl_3$) δ 8.18–8.12 (m, 2H), 7.90 (d, J = 2.7 Hz, 1H), 7.66–7.61 (m, 1H), 7.58 (dt, J = 7.8, 4.8 Hz, 1H), 7.52 (t, J = 7.5 Hz, 2H), 7.43 (t, J = 7.3 Hz, 1H), 7.20 (ddd, J = 12.2, 7.5, 0.9 Hz, 1H), 3.17 (d, J = 6.9 Hz, 3H);

^{13}C NMR (100 MHz, $CDCl_3$) δ 161.68, 159.14, 156.87 (d, J = 6.2 Hz), 150.75 (d, J = 1.3 Hz), 139.70 (d, J = 3.4 Hz), 139.40, 130.48 (d, J = 9.2 Hz), 128.92 (d, J = 10.8 Hz), 127.19, 123.81 (d, J = 4.4 Hz), 117.51 (d, J = 14.4 Hz), 114.50 (d, J = 3.7 Hz), 112.20 (d, J = 23.5 Hz), 27.47 (d, J = 10.2 Hz);

^{19}F NMR (376 MHz, $CDCl_3$) δ -109.00.

HRMS (EI-TOF) (M^+) calculated for $C_{16}H_{12}NF$ 237.0954, found 237.0956.

1,6,8-trimethyl-3-phenylisoquinoline (3ma)

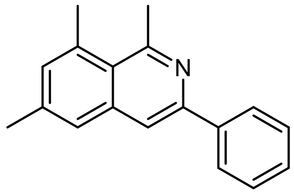

The title compound was prepared according to the general procedure and purified by flash column chromatography to give the corresponding product as a yellow oil, 14.9 mg, 30% yield.

^{1}H NMR (400 MHz, CDCl$_3$) δ 8.22–8.04 (m, 2H), 7.81 (s, 1H), 7.53–7.44 (m, 3H), 7.42–7.36 (m, 1H), 7.19 (s, 1H), 3.20 (s, 3H), 2.93 (s, 3H), 2.47 (s, 3H);
^{13}C NMR (100 MHz, CDCl$_3$) δ 158.47, 149.09, 139.78, 139.57, 139.54, 136.12, 132.83, 128.87, 128.41, 127.00, 126.08, 125.84, 115.93, 29.77, 25.81, 21.61;
HRMS (EI-TOF) (M $^+$) calculated for $C_{18}H_{17}N$ 247.1361, found 247.1361.

6,7-dimethoxy-1-methyl-3-phenylisoquinoline (3na)

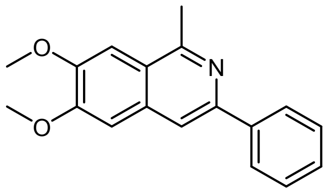

The title compound was prepared according to the general procedure and purified by flash column chromatography to give the corresponding product as a yellow oil, 31.3 mg, 56% yield.
^{1}H NMR (400 MHz, CDCl$_3$) δ 8.10 (d, J = 7.6 Hz, 2H), 7.80 (s, 1H), 7.49 (t, J = 7.6 Hz, 2H), 7.38 (t, J = 7.3 Hz, 1H), 7.29 (s, 1H), 7.12 (s, 1H), 4.06 (s, 3H), 4.05 (s, 3H), 2.97 (s, 3H);
^{13}C NMR (100 MHz, CDCl$_3$) δ 156.12, 152.79, 149.94, 149.34, 140.32, 133.62, 128.86, 128.18, 126.96, 122.42, 114.61, 105.83, 104.04, 56.23, 56.19, 22.93;
HRMS (EI-TOF) (M $^+$) calculated for $C_{18}H_{17}NO_2$ 279.1259, found 279.1257.

2-phenyl-8,9-dihydro-7*H*-benzo[*de*]quinoline (3oa)

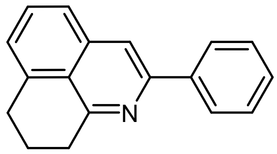

The title compound was prepared according to the general procedure and purified by flash column chromatography to give the corresponding product as a yellow oil, 29.4 mg, 60% yield.
^{1}H NMR (400 MHz, CDCl$_3$) δ 8.16–8.08 (m, 2H), 7.89 (s, 1H), 7.69 (d, J = 8.0 Hz, 1H), 7.57 (dd, J = 8.1, 7.1 Hz, 1H), 7.53–7.48 (m, 2H), 7.44–7.37 (m, 1H), 7.32 (dd, J = 7.0, 1.1 Hz, 1H), 3.39-3.32 (t, J = 6.1 Hz, 2H), 3.16 (t, J = 6.1 Hz, 2H), 2.28-2.21 (m, 2H);
^{13}C NMR (100 MHz, CDCl$_3$) δ 160.51, 150.32, 140.26, 138.97, 137.14, 130.30, 128.91, 128.44, 127.26, 124.98, 124.97, 124.53, 115.30, 34.84, 30.67, 23.58;
HRMS (EI-TOF) (M $^+$) calculated for $C_{18}H_{15}N$ 245.1204, found 245.1205.

1-ethyl-3-phenylisoquinoline (3pa)

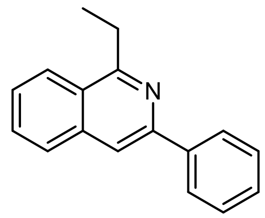

The title compound was prepared according to the general procedure and purified by flash column chromatography to give the corresponding product as a yellow oil, 10.7 mg, 23% yield.

^{1}H NMR (400 MHz, $CDCl_3$) *δ* 8.27–8.12 (m, 3H), 7.94 (s, 1H), 7.88 (d, *J* = 8.2 Hz, 1H), 7.71–7.64 (m, 1H), 7.57 (m, 1H), 7.55–7.47 (m, 2H), 7.45–7.37 (m, 1H), 3.42 (q, *J* = 7.5 Hz, 2H), 1.54 (t, *J* = 7.5 Hz, 3H);

^{13}C NMR (100 MHz, $CDCl_3$) *δ* 163.03, 150.02, 140.18, 137.27, 130.01, 128.91, 128.49, 128.03, 127.17, 126.90, 126.07, 125.41, 115.18, 28.72, 13.60;

HRMS (EI-TOF) (M^+) calculated for $C_{17}H_{15}N$ 233.1204, found 233.1205.

1-benzyl-3-phenylisoquinoline (3qa)

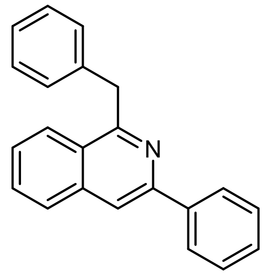

The title compound was prepared according to the general procedure and purified by flash column chromatography to give the corresponding product as a pale brown oil, 15.4 mg, 26% yield.

^{1}H NMR (400 MHz, $CDCl_3$) *δ* 8.21 (dd, *J* = 5.2, 3.3 Hz, 2H), 8.16 (d, *J* = 8.0 Hz, 1H), 8.00 (s, 1H), 7.87 (d, *J* = 8.2 Hz, 1H), 7.66–7.61 (m, 1H), 7.55–7.48 (m, 3H), 7.45–7.41 (m, 1H), 7.38 (d, *J* = 7.5 Hz, 2H), 7.31–7.26 (m , 2H), 7.19 (t, *J* = 7.3 Hz, 1H), 4.77 (s, 2H);

^{13}C NMR (100 MHz, $CDCl_3$) *δ* 160.14, 150.13, 139.93, 139.81, 137.68, 130.11, 128.96, 128.94, 128.65, 128.60, 128.00, 127.19, 127.13, 126.44, 126.39, 126.01, 115.77, 42.47;

HRMS (EI-TOF) (M^+) calculated for $C_{22}H_{17}N$ 295.1361, found 295.1363.

1-methyl-3-(*p*-tolyl)isoquinoline (3ab)

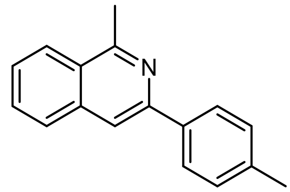

The title compound was prepared according to the general procedure and purified by flash column chromatography to give the corresponding product as a yellow oil, 31.7 mg, 68% yield.

^{1}H NMR (400 MHz, $CDCl_3$) δ 8.12 (d, J = 8.3 Hz, 1H), 8.06 (d, J = 8.2 Hz, 2H), 7.90 (s, 1H), 7.85 (d, J = 8.2 Hz, 1H), 7.69–7.64 (m, 1H), 7.59–7.54 (m, 1H), 7.32 (d, J = 8.1 Hz, 2H), 3.05 (s, 3H), 2.44 (s, 3H);
^{13}C NMR (100 MHz, $CDCl_3$) δ 158.65, 150.22, 138.36, 137.20, 136.98, 130.14, 129.64, 127.73, 127.01, 126.75, 126.64, 125.81, 114.93, 22.87, 21.47;
HRMS (EI-TOF) (M^{+}) calculated for $C_{17}H_{15}N$ 233.1204, found 233.1206.

3-(4-methoxyphenyl)-1-methylisoquinoline (3ac)

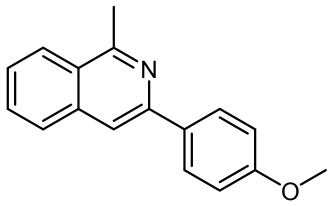

The title compound was prepared according to the general procedure and purified by flash column chromatography to give the corresponding product as a yellow oil, 30.4 mg, 61% yield.
^{1}H NMR (400 MHz, $CDCl_3$) δ 8.13–8.09 (m, 3H), 7.86–7.81 (m, 2H), 7.68–7.63 (m, 1H), 7.54 (m, 1H), 7.07–7.02 (m, 2H), 3.89 (s, 3H), 3.04 (s, 3H);
^{13}C NMR (100 MHz, $CDCl_3$) δ 160.15, 158.61, 149.91, 137.04, 132.69, 130.13, 128.36, 127.63, 126.58, 126.42, 125.81, 114.31, 114.28, 55.55, 22.86;
HRMS (EI-TOF) (M^{+}) calculated for $C_{17}H_{15}NO$ 249.1154, found 249.1153.

3-(4-fluorophenyl)-1-methylisoquinoline (3ad)

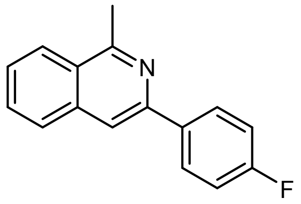

The title compound was prepared according to the general procedure and purified by flash column chromatography to give the corresponding product as a yellow oil, 15.4 mg, 26% yield.
^{1}H NMR (400 MHz, $CDCl_3$) δ 8.17–8.09 (m, 3H), 7.86 (d, J = 11.2 Hz, 2H), 7.71–7.65 (m, 1H), 7.60–7.56 (m, 1H), 7.19 (t, J = 8.7 Hz, 2H), 3.04 (s, 3H);
^{13}C NMR (100 MHz, $CDCl_3$) δ 164.61, 162.14, 158.88, 149.23, 136.94, 136.19 (d, J = 2.8 Hz), 130.35, 128.89 (d, J = 8.2 Hz), 127.77, 126.88 (d, J = 35.5 Hz), 125.87, 115.78 (d, J = 21.7 Hz), 115.11, 22.86;
^{19}F NMR (376 MHz, $CDCl_3$) δ -114.23;
HRMS (EI-TOF) (M^{+}) calculated for $C_{16}H_{12}NF$ 237.0954, found 237.0954.

3-(4-chlorophenyl)-1-methylisoquinoline (3ae)

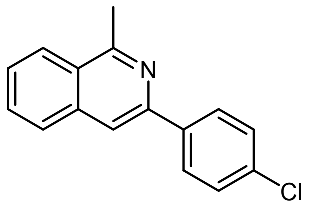

The title compound was prepared according to the general procedure and purified by flash column chromatography to give the corresponding product as a yellow oil, 27.9 mg, 55% yield.

^{1}H NMR (400 MHz, $CDCl_3$) δ 8.14 (dd, J = 8.4, 0.6 Hz, 1H), 8.12–8.07 (m, 2H), 7.90 (s, 1H), 7.86 (d, J = 8.2 Hz, 1H), 7.71–7.67 m, 1H), 7.61–7.57 (m, 1H), 7.50–7.43 (m, 2H), 3.04 (s, 3H);

^{13}C NMR (100 MHz, $CDCl_3$) δ 158.97, 148.92, 138.46, 136.87, 134.53, 130.40, 129.06, 128.41, 127.84, 127.24, 126.88, 125.88, 115.35, 22.87;

HRMS (EI-TOF) (M $^+$) calculated for $C_{16}H_{12}NCl$ 253.0658, found 253.0657.

3-(4-bromophenyl)-1-methylisoquinoline (3af)

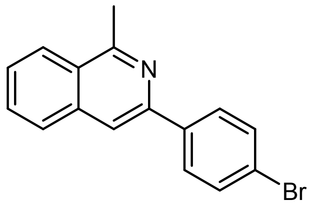

The title compound was prepared according to the general procedure and purified by flash column chromatography to give the corresponding product as a yellow oil, 29.8 mg, 50% yield.

^{1}H NMR (400 MHz, $CDCl_3$) δ 8.14 (d, J = 8.3 Hz, 1H), 8.08–7.98 (m, 2H), 7.91 (s, 1H), 7.86 (d, J = 8.2 Hz, 1H), 7.72–7.66 (m, 1H), 7.66–7.55 (m, 3H), 3.04 (s, 3H);

^{13}C NMR (100 MHz, $CDCl_3$) δ 159.00, 148.94, 138.92, 136.87, 132.02, 130.42, 128.73, 127.85, 127.27, 126.92, 125.90, 122.85, 115.35, 22.87;

HRMS (EI-TOF) (M $^+$) calculated for $C_{16}H_{12}NBr$ 297.0153, found 297.0153.

1-methyl-3-(4-(trifluoromethyl)phenyl)isoquinoline (3ag)

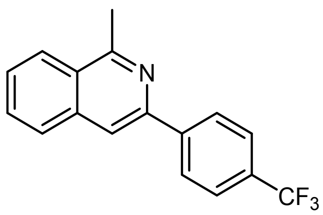

The title compound was prepared according to the general procedure and purified by flash column chromatography to give the corresponding product as a yellow oil, 26.4 mg, 46% yield.

^{1}H NMR (400 MHz, $CDCl_3$) δ 8.26 (d, J = 8.2 Hz, 2H), 8.16 (d, J = 8.3 Hz, 1H), 7.98 (s, 1H), 7.89 (d, J = 8.1 Hz, 1H), 7.77–7.69 (m, 3H), 7.66–7.59 (m, 1H), 3.06 (s, 3H);

^{13}C NMR (100 MHz, $CDCl_3$) δ 159.20, 148.56, 143.40, 136.77, 130.53, 130.47, 130.15, 127.98, 127.63, 127.38, 127.18, 125.84 (q, J = 3.7 Hz), 123.21, 116.23, 22.87;

^{19}F NMR (376 MHz, $CDCl_3$) δ -62.79;

HRMS (EI-TOF) (M^+) calculated for $C_{17}H_{12}NF_3$ 287.0922, found 287.0921.

1-methyl-3-(m-tolyl)isoquinoline (3ah)

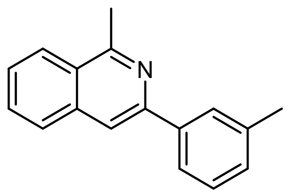

The title compound was prepared according to the general procedure and purified by flash column chromatography to give the corresponding product as a yellow oil, 28.0 mg, 61% yield.

^{1}H NMR (400 MHz, $CDCl_3$) δ 8.14 (d, J = 8.4 Hz, 1H), 7.99 (s, 1H), 7.92 (t, J = 3.5 Hz, 2H), 7.86 (d, J = 8.2 Hz, 1H), 7.71–7.65 (m, 1H), 7.60–7.55 (m, 1H), 7.41 (t, J = 7.6 Hz, 1H), 7.24 (d, J = 7.5 Hz, 1H), 3.06 (s, 3H), 2.49 (s, 3H);

^{13}C NMR (100 MHz, $CDCl_3$) δ 158.71, 150.38, 139.99, 138.50, 136.96, 130.18, 129.29, 128.82, 127.92, 127.79, 126.90, 126.75, 125.83, 124.28, 115.47, 22.87, 21.83.

HRMS (EI-TOF) (M^+) calculated for $C_{17}H_{15}N$ 233.1204, found 233.1207.

3-(3-methoxyphenyl)-1-methylisoquinoline (3ai)

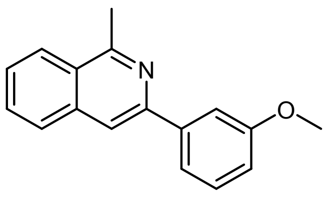

The title compound was prepared according to the general procedure and purified by flash column chromatography to give the corresponding product as a yellow oil, 25.4 mg, 51% yield.

^{1}H NMR (400 MHz, $CDCl_3$) δ 8.14 (d, J = 8.7 Hz, 1H), 7.92 (s, 1H), 7.87 (d, J = 8.2 Hz, 1H), 7.77–7.73 (m, 1H), 7.69 (m, 2H), 7.58 (m, 1H), 7.42 (t, J = 7.9 Hz, 1H), 6.97 (m, 1H), 3.94 (s, 3H), 3.05 (s, 3H);

^{13}C NMR (100 MHz, $CDCl_3$) δ 160.26, 158.75, 149.96, 141.56, 136.91, 130.24, 129.90, 127.85, 127.04, 126.88, 125.84, 119.61, 115.63, 114.40, 112.53, 55.58, 22.88;

HRMS (EI-TOF) (M^+) calculated for $C_{17}H_{15}NO$ 249.1154, found 249.1152.

3-(3-chlorophenyl)-1-methylisoquinoline (3aj)

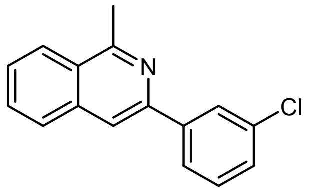

The title compound was prepared according to the general procedure and purified by flash column

chromatography to give the corresponding product as a yellow oil, 22.8 mg, 45% yield.

^{1}H NMR (400 MHz, $CDCl_3$) δ 8.24–8.11 (m, 2H), 8.02 (d, J = 7.6 Hz, 1H), 7.92 (s, 1H), 7.88 (d, J = 8.1 Hz, 1H), 7.73–7.67 (m, 1H), 7.64–7.58 (m, 1H), 7.43 (t, J = 7.8 Hz, 1H), 7.37 (dd, J = 6.8, 1.8 Hz, 1H), 3.05 (s, 3H);

^{13}C NMR (100 MHz, $CDCl_3$) δ 159.06, 148.66, 141.88, 136.83, 135.00, 130.45, 130.14, 128.48, 127.92, 127.40, 127.34, 127.05, 125.90, 125.17, 115.77, 22.86;

HRMS (EI-TOF) (M^+) calculated for $C_{16}H_{12}NCl$ 253.0658, found 253.0656.

1-methyl-3-(*o*-tolyl)isoquinoline (3ak)

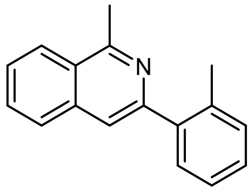

The title compound was prepared according to the general procedure and purified by flash column chromatography to give the corresponding product as a yellow oil, 24.7 mg, 53% yield.

^{1}H NMR (400 MHz, $CDCl_3$) δ 8.18 (d, J = 8.3 Hz, 1H), 7.85 (d, J = 8.1 Hz, 1H), 7.73–7.68 (m, 1H), 7.65-7.59 (m, 2H), 7.52–7.48 (m, 1H), 7.31 (dd, J = 7.0, 3.4 Hz, 3H), 3.04 (s, 3H), 2.43 (s, 3H);

^{13}C NMR (100 MHz, $CDCl_3$) δ 158.19, 152.70, 140.94, 136.54, 136.35, 130.91, 130.22, 130.16, 128.16, 127.60, 127.04, 126.24, 126.07, 125.81, 118.98, 22.68, 20.69;

HRMS (EI-TOF) (M^+) calculated for $C_{17}H_{15}N$ 233.1204, found 233.1201.

3-(2-methoxyphenyl)-1-methylisoquinoline (3al)

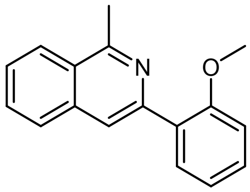

The title compound was prepared according to the general procedure and purified by flash column chromatography to give the corresponding product as a yellow oil, 33.4 mg, 67% yield.

^{1}H NMR (400 MHz, $CDCl_3$) δ 8.13 (d, J = 8.3 Hz, 1H), 8.07 (s, 1H), 7.95 (d, J = 7.5 Hz, 1H), 7.85 (d, J = 8.1 Hz, 1H), 7.67 (t, J = 7.4 Hz, 1H), 7.58 (t, J = 7.5 Hz, 1H), 7.38 (t, J = 7.7 Hz, 1H), 7.14 (t, J = 7.4 Hz, 1H), 7.05 (d, J = 8.2 Hz, 1H), 3.90 (s, 3H), 3.05 (s, 3H);

^{13}C NMR (100 MHz, $CDCl_3$) δ 158.21, 157.29, 148.08, 136.44, 131.62, 129.94, 129.62, 129.45, 127.90, 126.88, 126.42, 125.65, 121.25, 120.09, 111.67, 55.89, 22.72;

HRMS (EI-TOF) (M^+) calculated for $C_{17}H_{15}NO$ 249.1154, found 249.1152.

3-(2-fluorophenyl)-1-methylisoquinoline (3am)

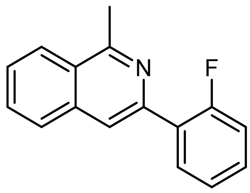

The title compound was prepared according to the general procedure and purified by flash column chromatography to give the corresponding product as a yellow oil, 26.6 mg, 56% yield.

^{1}H NMR (400 MHz, $CDCl_3$) δ 8.20–8.14 (m, 2H), 8.05 (s, 1H), 7.88 (d, *J* = 8.1 Hz, 1H), 7.74–7.66 (m, 1H), 7.63–7.59 (m, 1H), 7.39–7.34 (m, 1H), 7.33–7.29 (m, 1H), 7.22–7.17 (m, 1H), 3.05 (s, 3H);

^{13}C NMR (100 MHz, $CDCl_3$) δ 162.05, 159.57, 158.76, 145.40, 136.58, 131.48 (d, *J* = 2.8 Hz), 130.29, 129.84 (d, *J* = 8.3 Hz), 128.05, 127.42, 126.78, 125.74, 124.68 (d, *J* = 3.6 Hz), 119.97 (d, *J* = 9.7 Hz), 116.35 (d, *J* = 22.9 Hz), 22.80;

^{19}F NMR (376 MHz, $CDCl_3$) δ -116.81;

HRMS (EI-TOF) (M^+) calculated for $C_{16}H_{12}NF$ 237.0954, found 237.0953.

3-(furan-2-yl)-1-methylisoquinoline (3an)

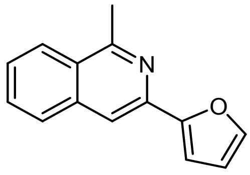

The title compound was prepared according to the general procedure and purified by flash column chromatography to give the corresponding product as a yellow oil, 14.6 mg, 35% yield.

^{1}H NMR (400 MHz, $CDCl_3$) δ 8.09 (d, *J* = 8.4 Hz, 1H), 7.88 (s, 1H), 7.83 (d, *J* = 8.2 Hz, 1H), 7.65 (t, *J* = 7.5 Hz, 1H), 7.61–7.48 (m, 2H), 7.13 (d, *J* = 3.3 Hz, 1H), 6.56 (dd, *J* = 3.3, 1.7 Hz, 1H), 3.00 (s, 3H) ;

^{13}C NMR (100 MHz, $CDCl_3$) δ 159.15, 154.56, 143.08, 142.43, 136.66, 130.43, 127.78, 126.87, 126.82, 125.96, 113.19, 112.16, 108.23, 22.81;

HRMS (EI-TOF) (M^+) calculated for $C_{14}H_{11}NO$ 209.0841, found 209.0841.

3-(*tert*-butyl)-1-methylisoquinoline (3ao)

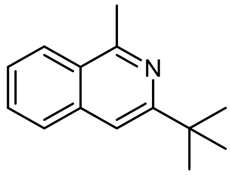

The title compound was prepared according to the general procedure and purified by flash column chromatography to give the corresponding product as a colorless oil, 16.3 mg, 41% yield.

^{1}H NMR (400 MHz, $CDCl_3$) δ 8.07 (dd, *J* = 8.4, 0.6 Hz, 1H), 7.76 (d, *J* = 8.2 Hz, 1H), 7.61 (m, 1H), 7.51 (m, 1H), 7.46 (s, 1H), 2.96 (s, 3H), 1.46 (s, 9H);

^{13}**C NMR** (100 MHz, $CDCl_3$) δ 161.77, 157.24, 136.58, 129.40, 127.25, 125.95, 125.63, 125.36, 112.58, 36.93, 30.16, 22.60;

HRMS (EI-TOF) (M^+) calculated for $C_{14}H_{17}N$ 199.1361, found 199.1362.

3-cyclohexyl-1-methylisoquinoline (3ap)

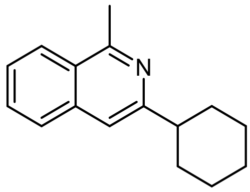

The title compound was prepared according to the general procedure and purified by flash column chromatography to give the corresponding product as a colorless oil, 25.0 mg, 56% yield.

^{1}**H NMR** (400 MHz, $CDCl_3$) δ 8.07 (d, J = 9.0 Hz, 1H), 7.75 (d, J = 8.2 Hz, 1H), 7.62 (m, 1H), 7.51 (m, 1H), 7.32 (s, 1H), 2.95 (s, 3H), 2.82 (m, 1H), 2.11 (d, J = 10.9 Hz, 2H), 1.93–1.85 (m, 2H), 1.79 (d, J = 12.7 Hz, 1H), 1.60–1.44 (m, 4H), 1.32 (m, 1H);

^{13}**C NMR** (100 MHz, $CDCl_3$) δ 159.24, 157.91, 136.92, 129.82, 127.20, 126.21, 126.14, 125.69, 114.54, 46.27, 33.47, 26.93, 26.46, 22.59;

HRMS (EI-TOF) (M^+) calculated for $C_{16}H_{19}N$ 225.1517, found 225.1514.

3-((3*r*,5*r*,7*r*)-adamantan-1-yl)-1-methylisoquinoline (3aq)

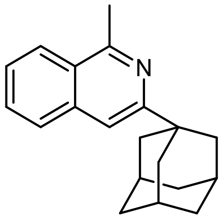

The title compound was prepared according to the general procedure and purified by flash column chromatography to give the corresponding product as a pale brown solid, m.p=107-109 ºC, 44.3 mg, 80% yield.

1H NMR (400 MHz, CDCl3) δ 8.07 (d, J = 8.3 Hz, 1H), 7.77 (d, J = 8.1 Hz, 1H), 7.61 (t, J = 7.4 Hz, 1H), 7.51 (t, J = 7.5 Hz, 1H), 7.38 (s, 1H), 2.97 (s, 3H), 2.16 (s, 3H), 2.12 (d, J = 2.3 Hz, 6H), 1.85 (s, 6H);

13C NMR (100 MHz, CDCl3) δ 161.93, 157.43, 136.84, 129.49, 127.48, 126.01 (d, J = 10.2 Hz), 125.54, 112.86, 42.09, 38.60, 37.21, 29.10, 22.83;

HRMS (EI-TOF) (M^+) calculated for $C_{20}H_{23}N$ 277.1830, found 277.1832.

7. NMR Spectra of Products

^{1}H NMR and ^{13}C NMR spectra of **3aa**

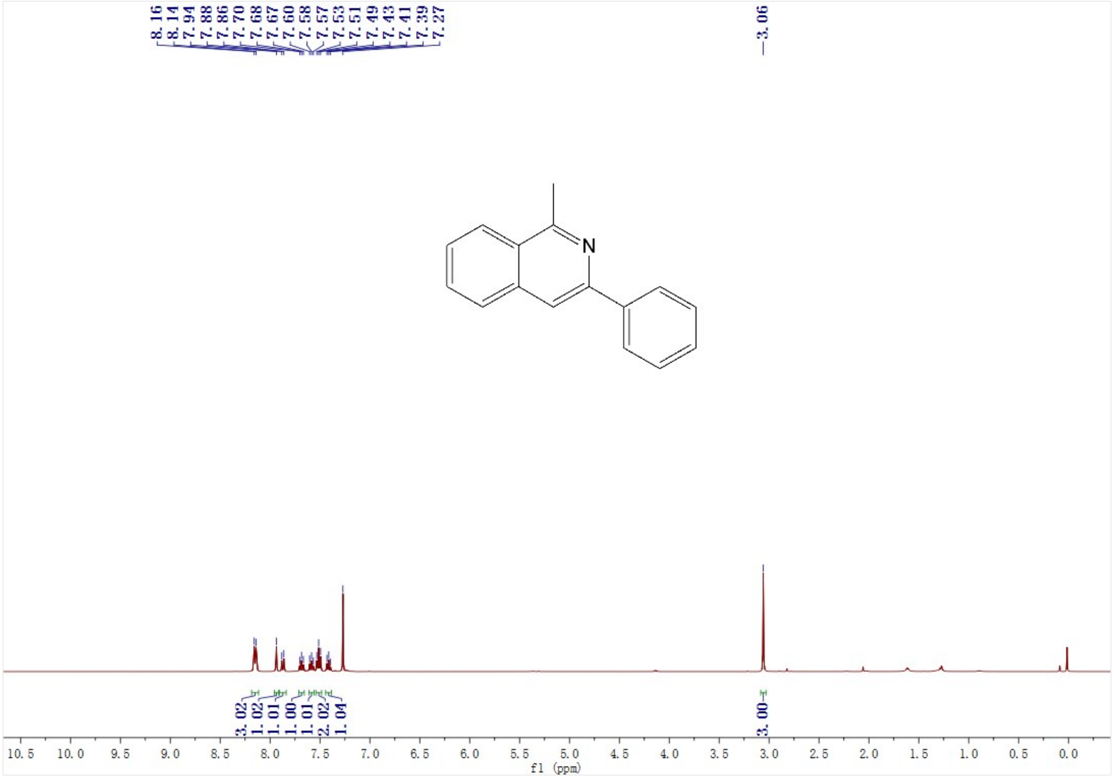

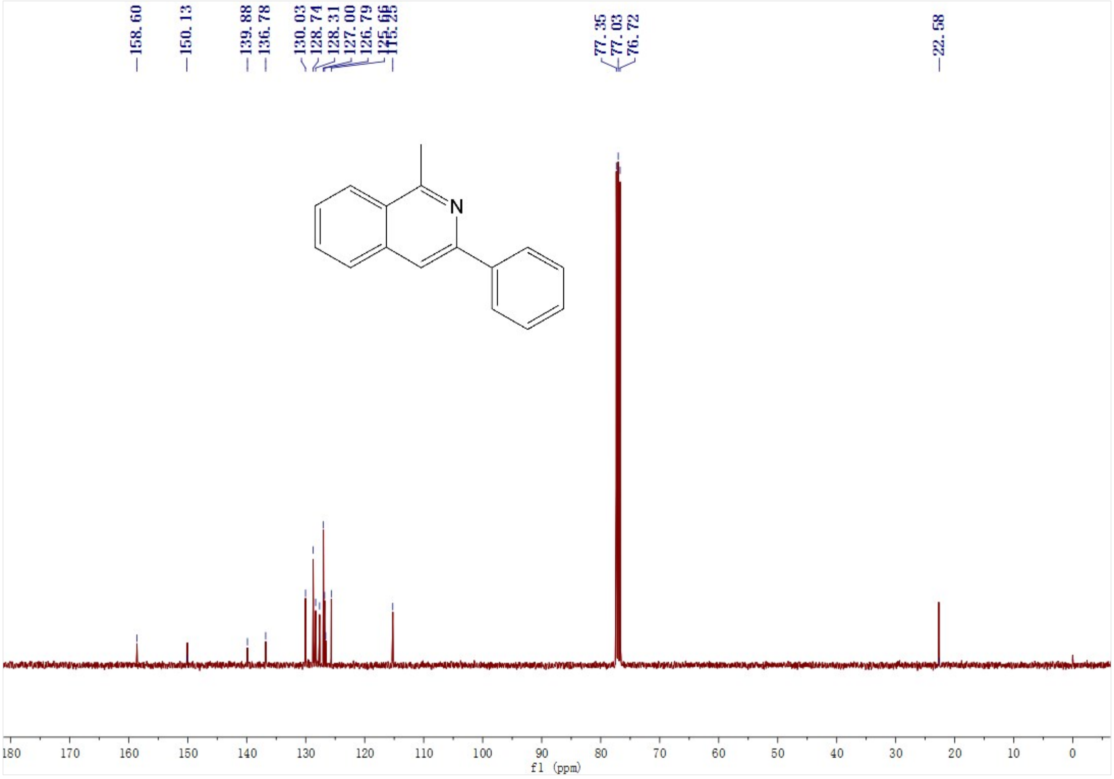

^{1}H NMR and ^{13}C NMR spectra of **3ba**

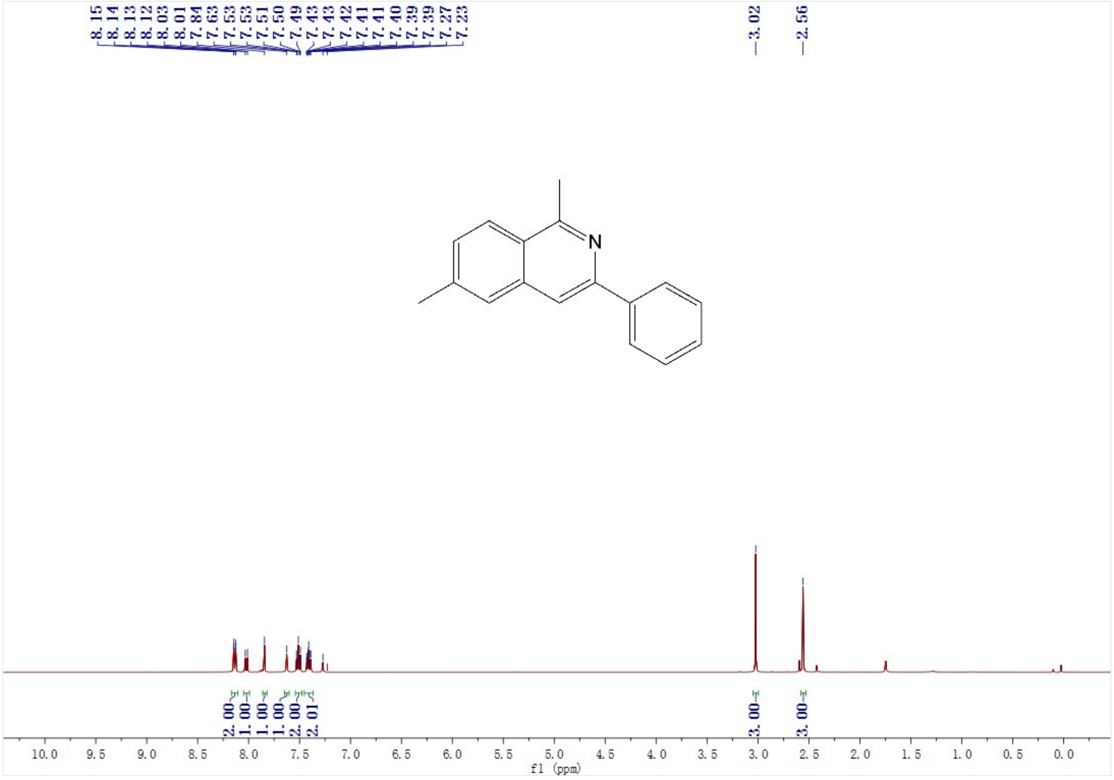

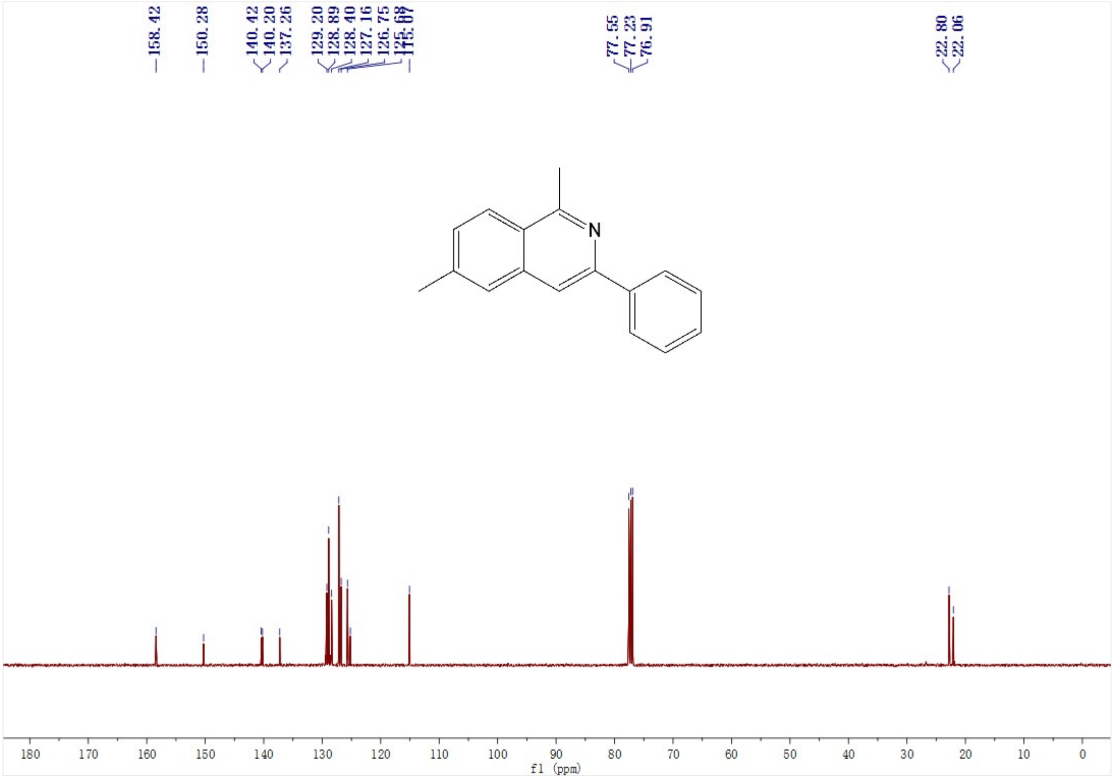

^{1}H NMR and ^{13}C NMR spectra of **3ca**

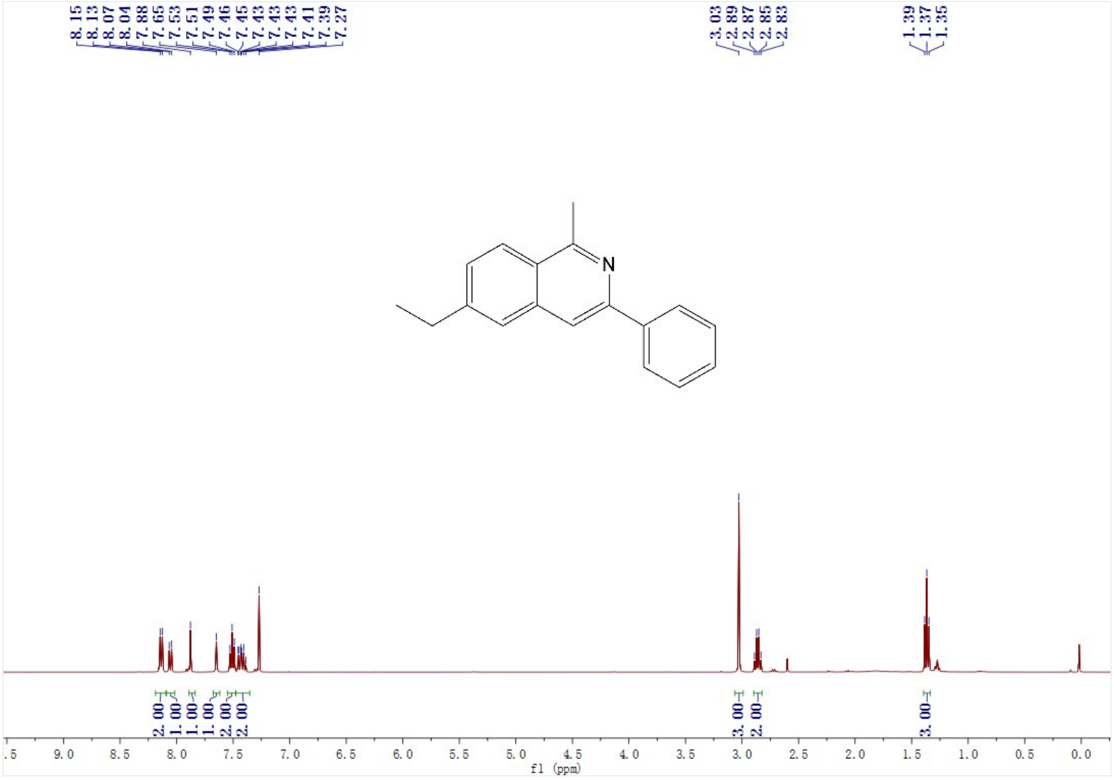

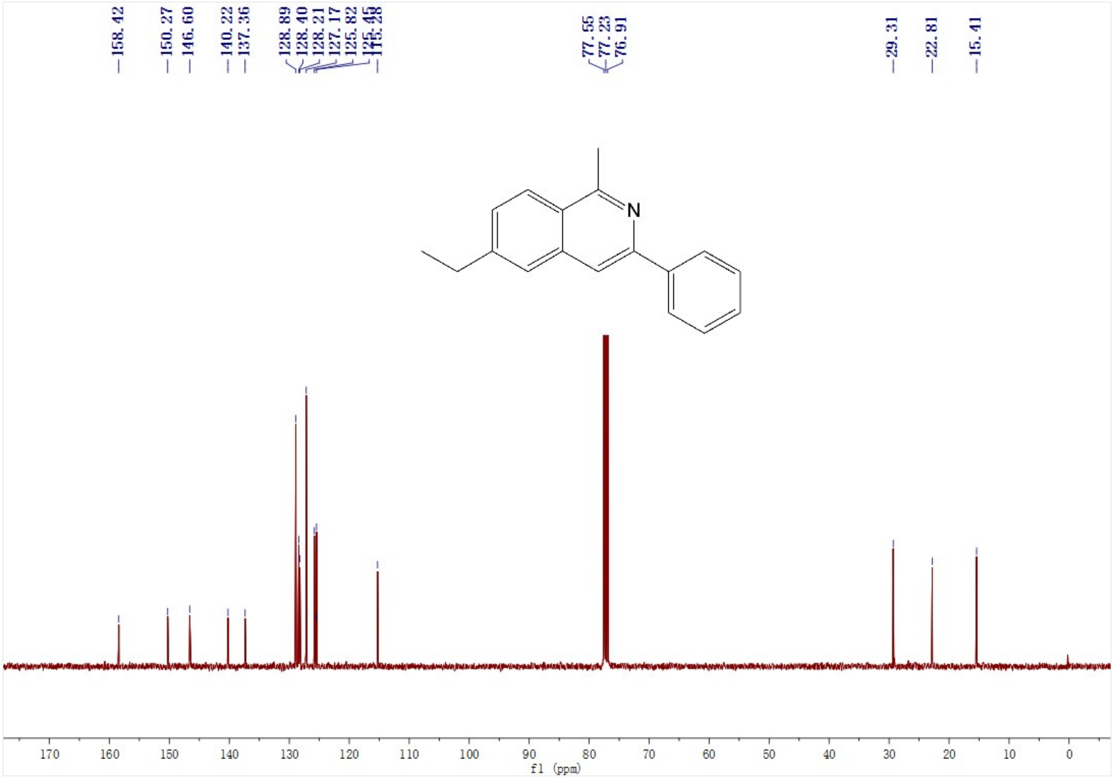

^{1}H NMR and ^{13}C NMR spectra of **3da**

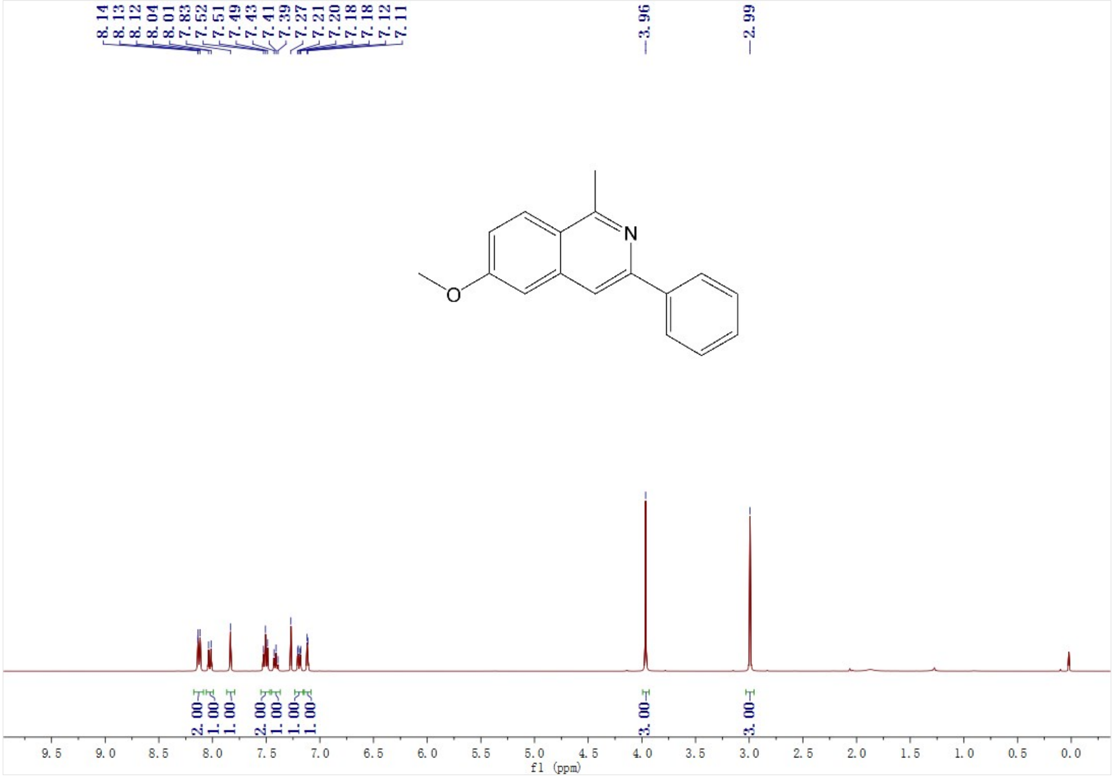

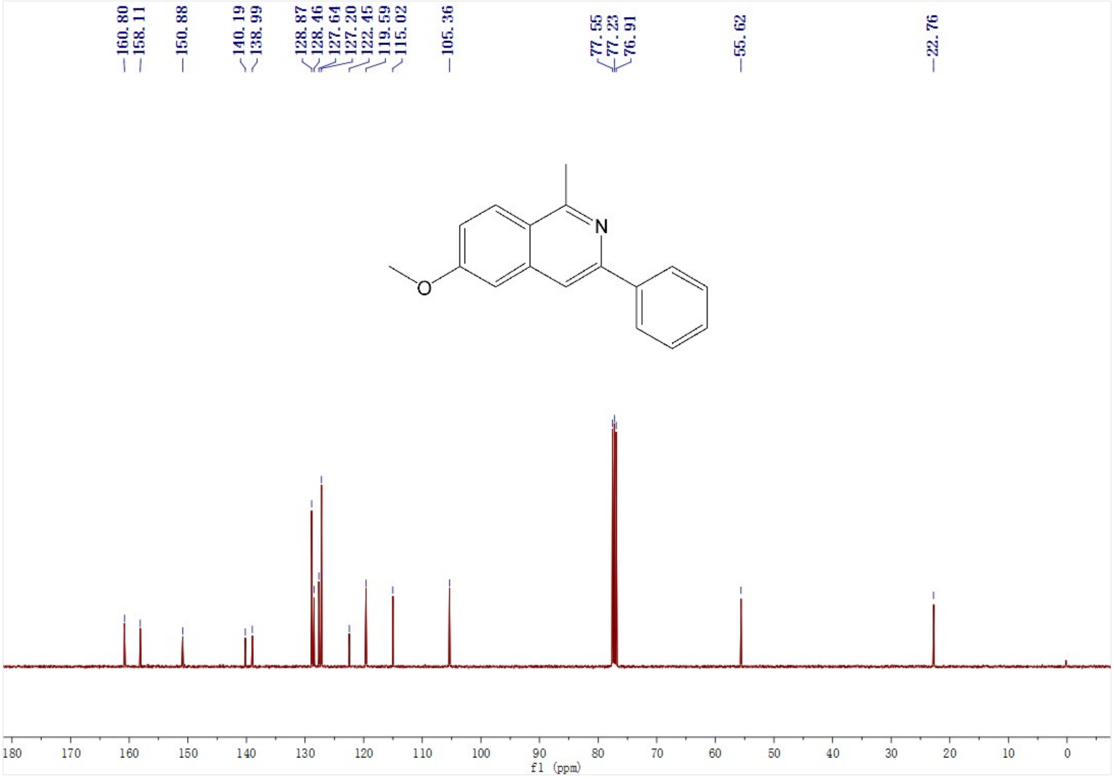

^{1}H NMR and ^{13}C NMR spectra of **3ea**

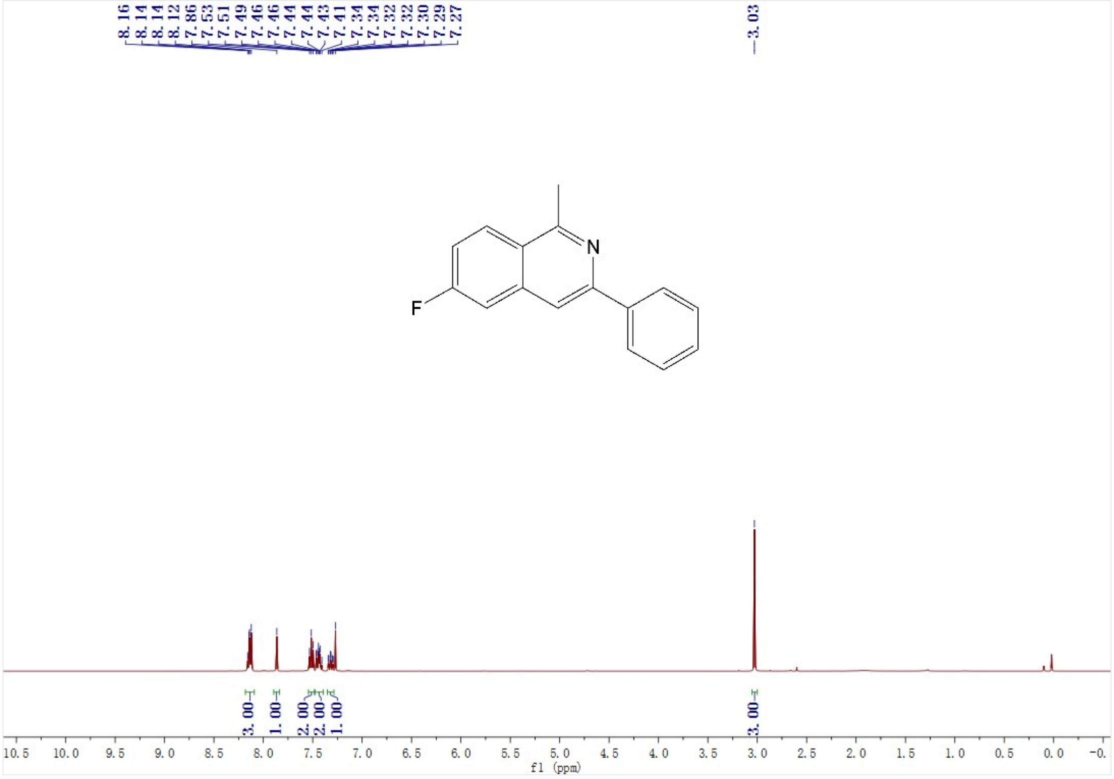

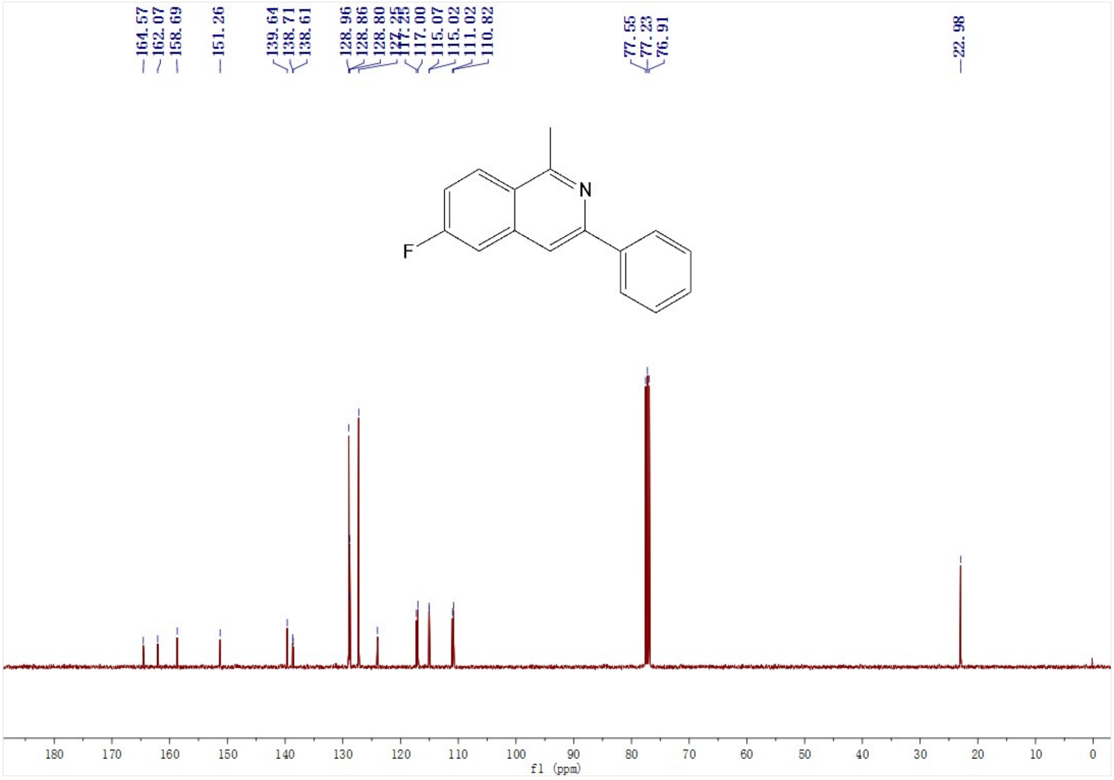

^{19}F NMR spectra of **3ea**

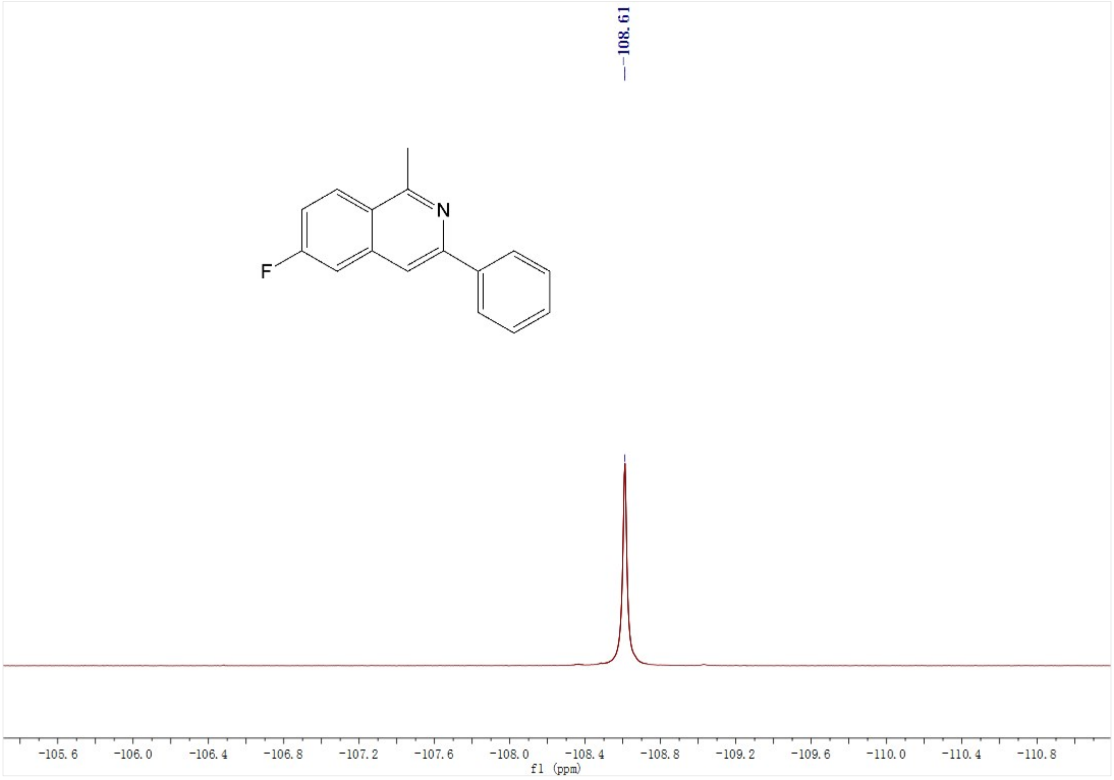

^{1}H NMR and ^{13}C NMR spectra of **3fa**

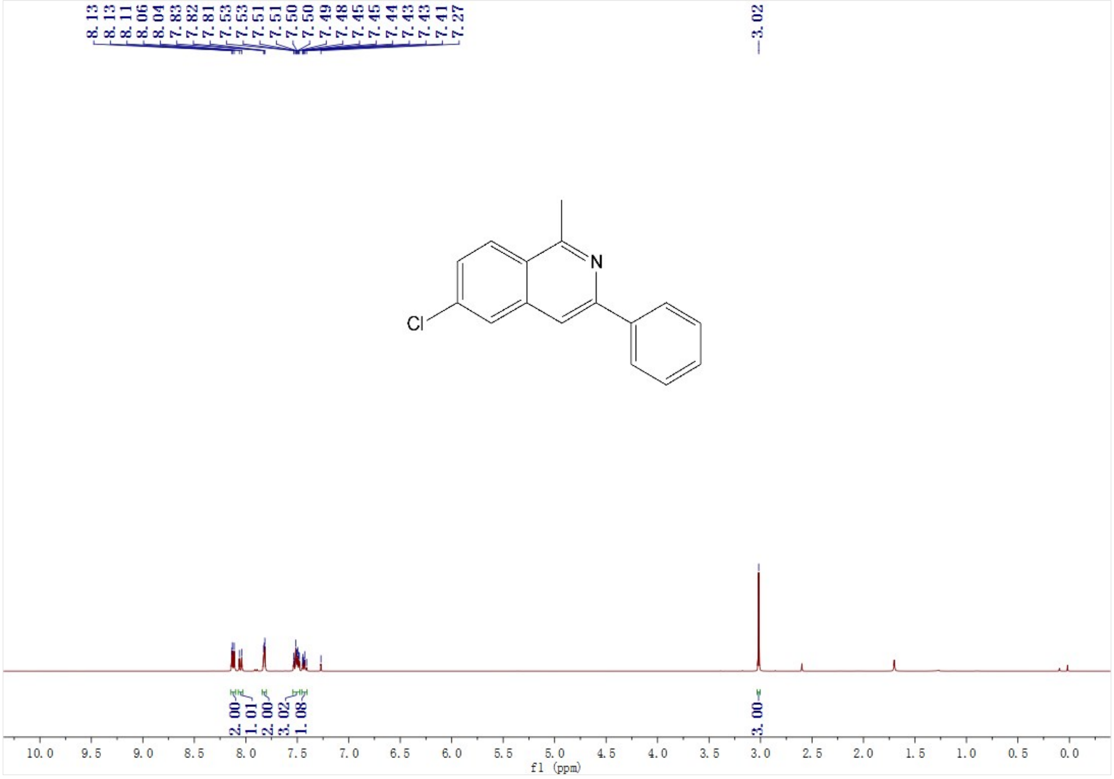

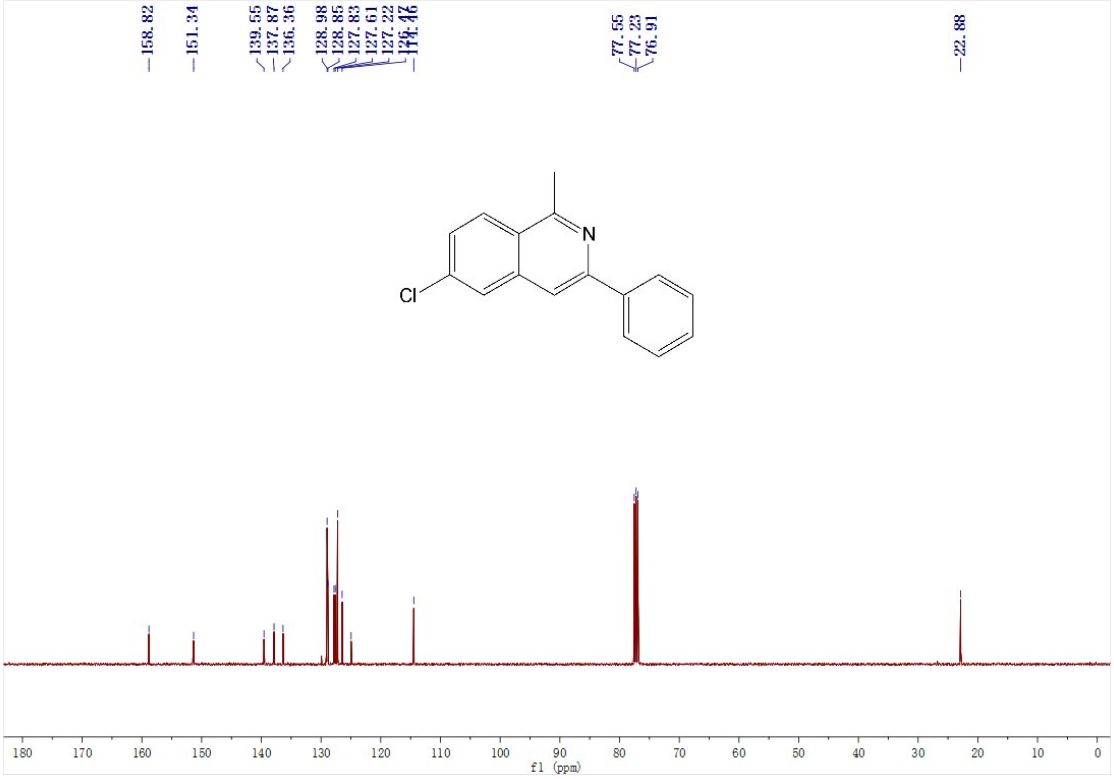

^{1}H NMR and ^{13}C NMR spectra of **3ga**

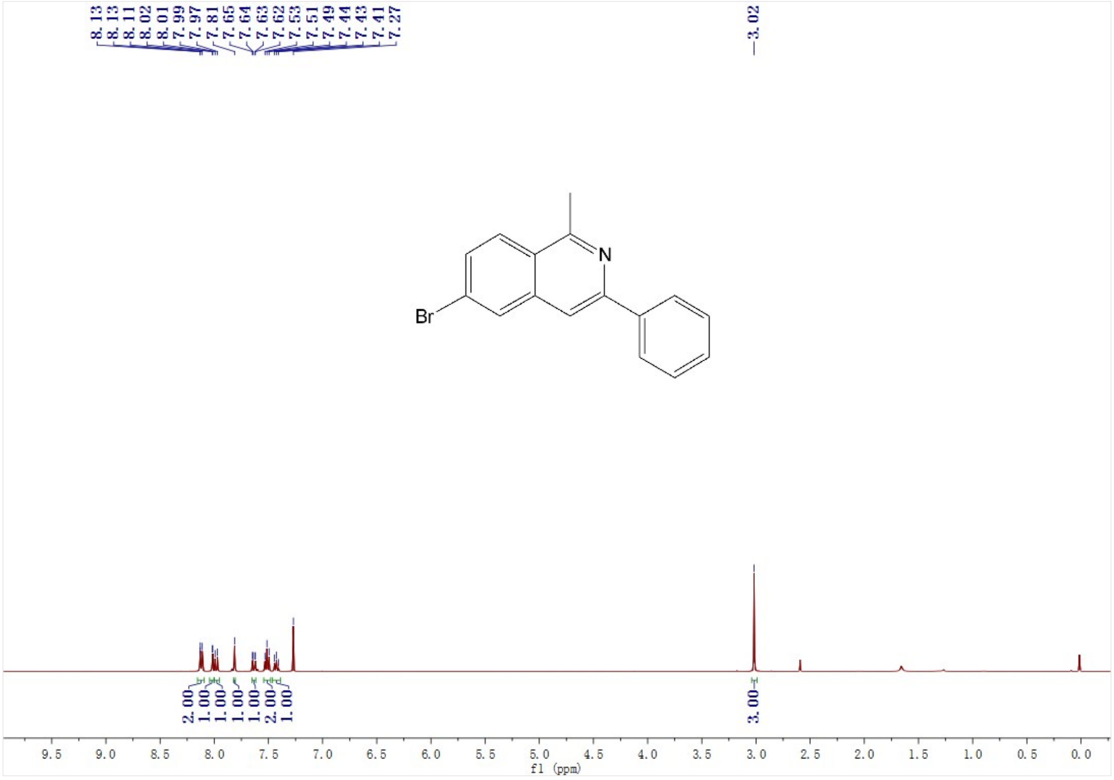

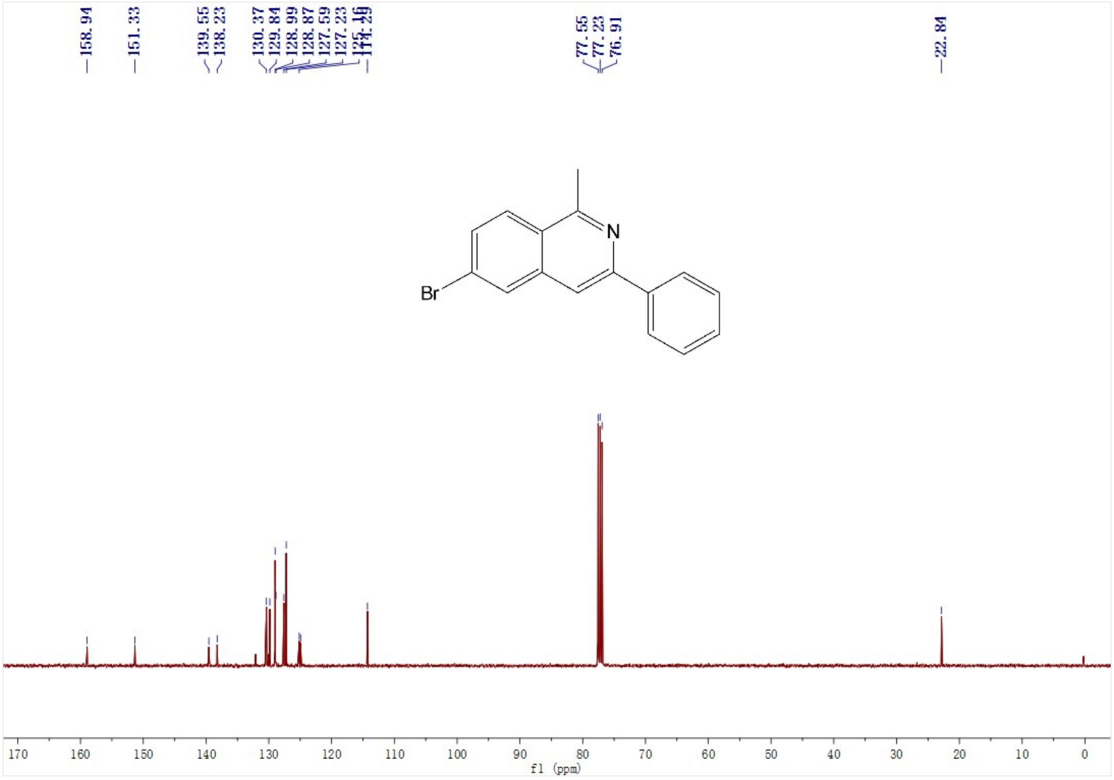

^{1}H NMR and ^{13}C NMR spectra of **3ha**

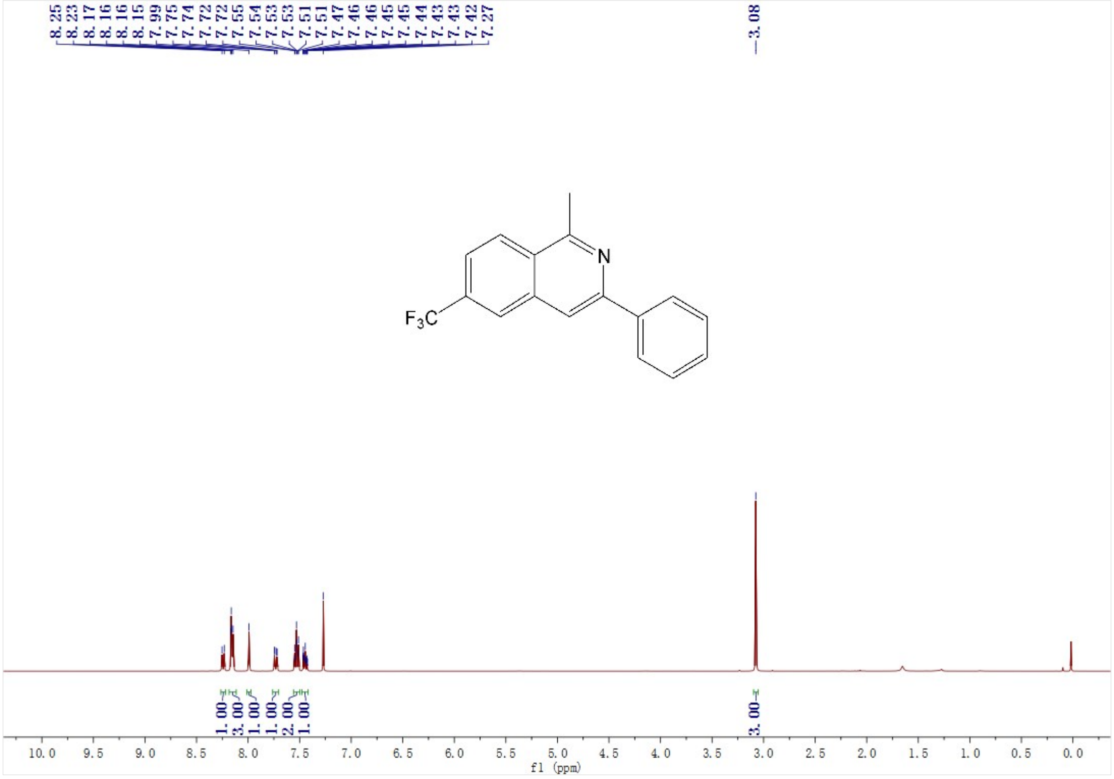

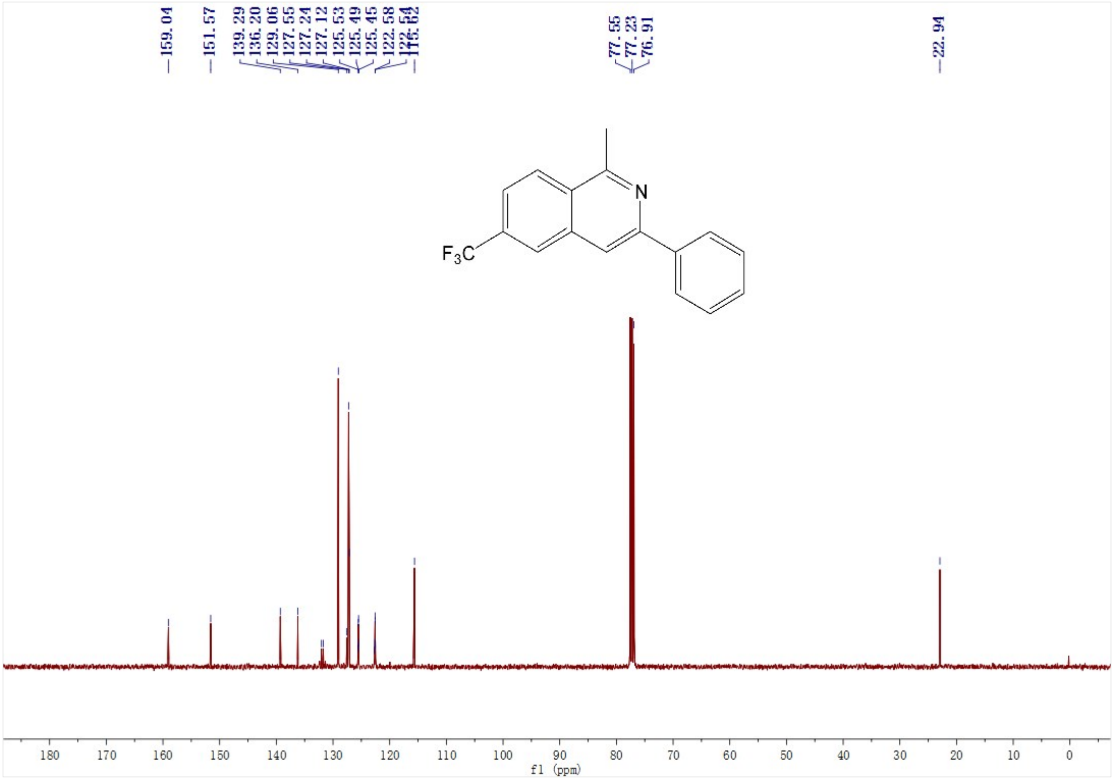

^{19}F NMR spectra of **3ha**

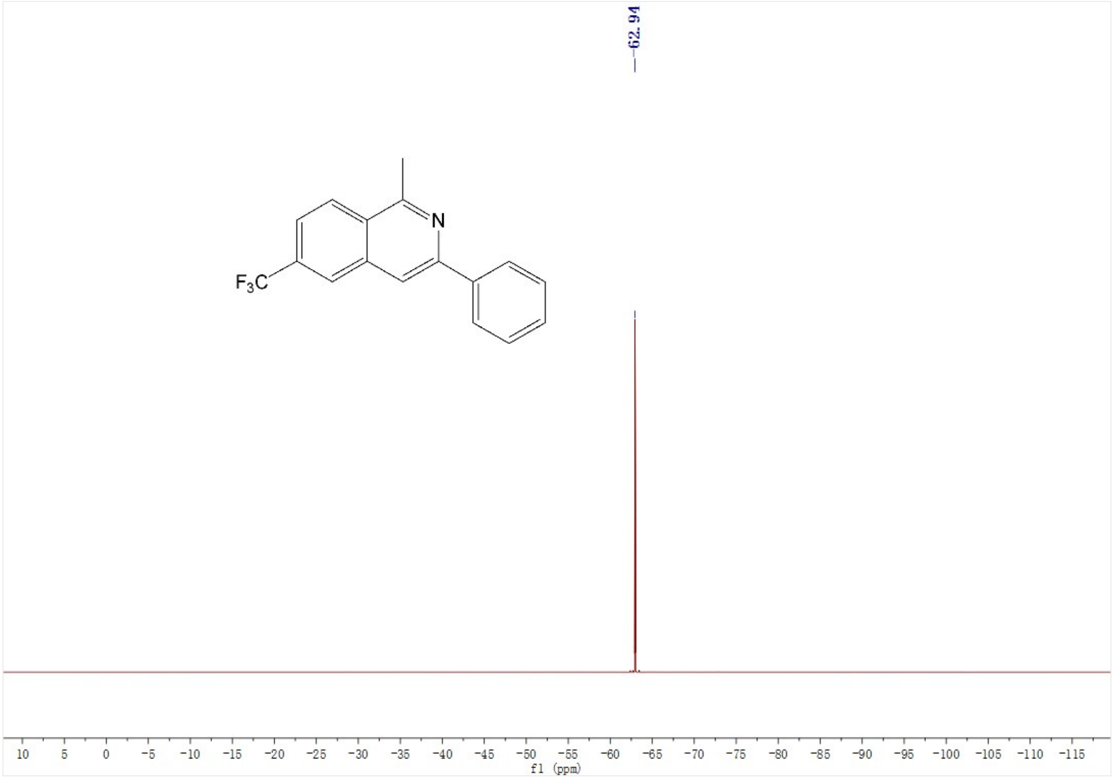

^{1}H NMR and ^{13}C NMR spectra of **3ia**

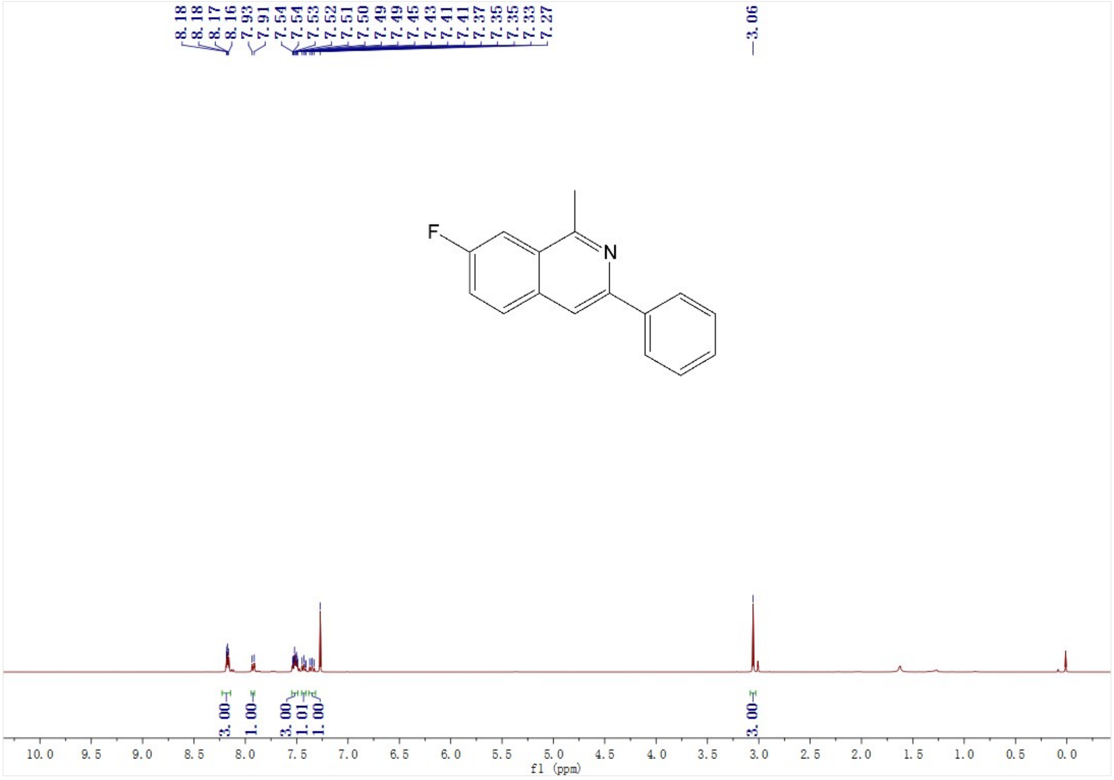

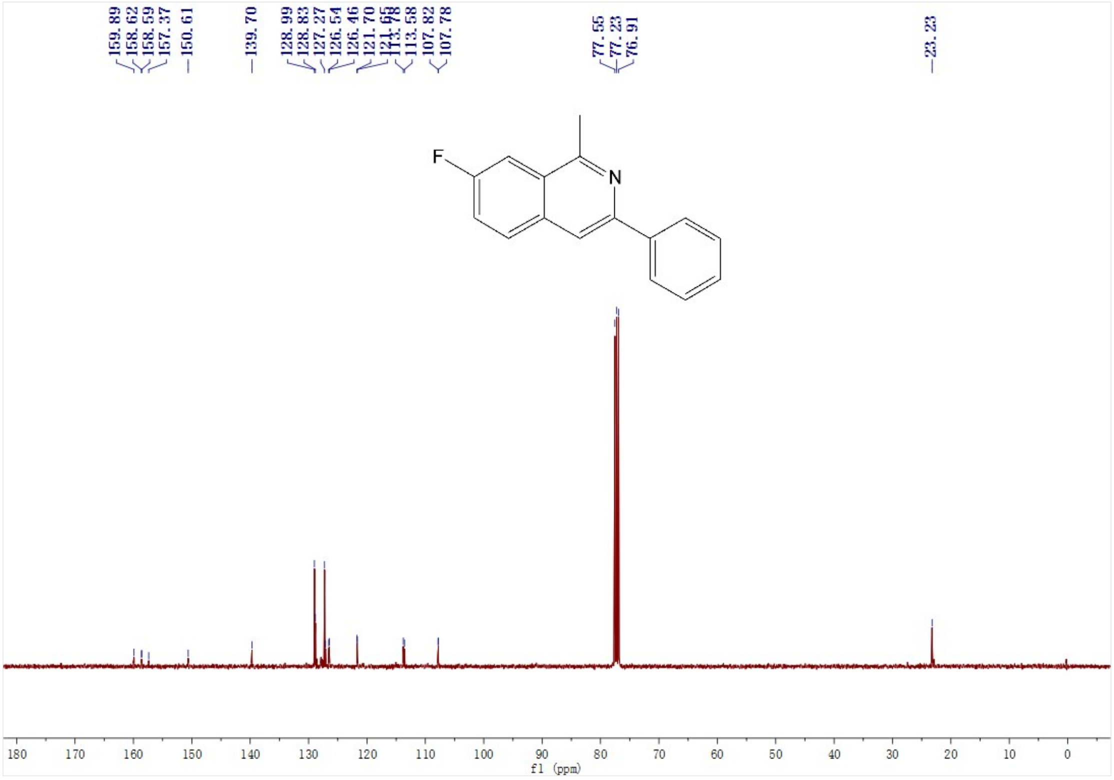

^{19}F NMR spectra of **3ia**

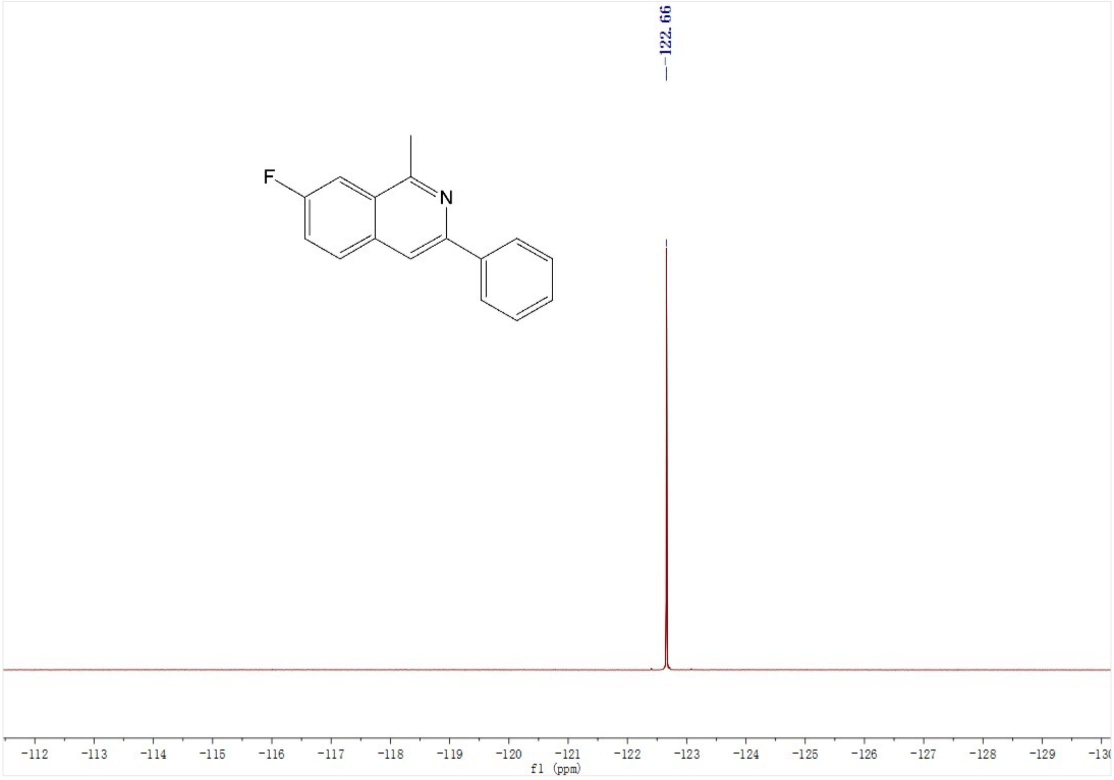

^{1}H NMR and ^{13}C NMR spectra of **3ja**

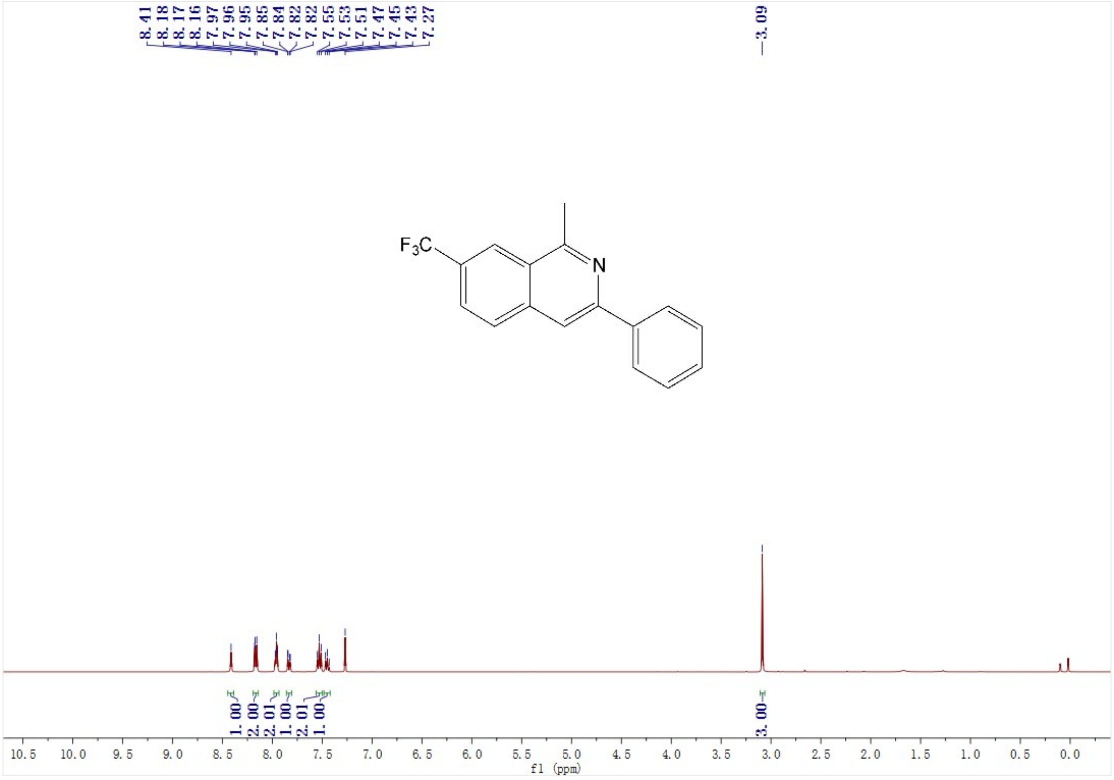

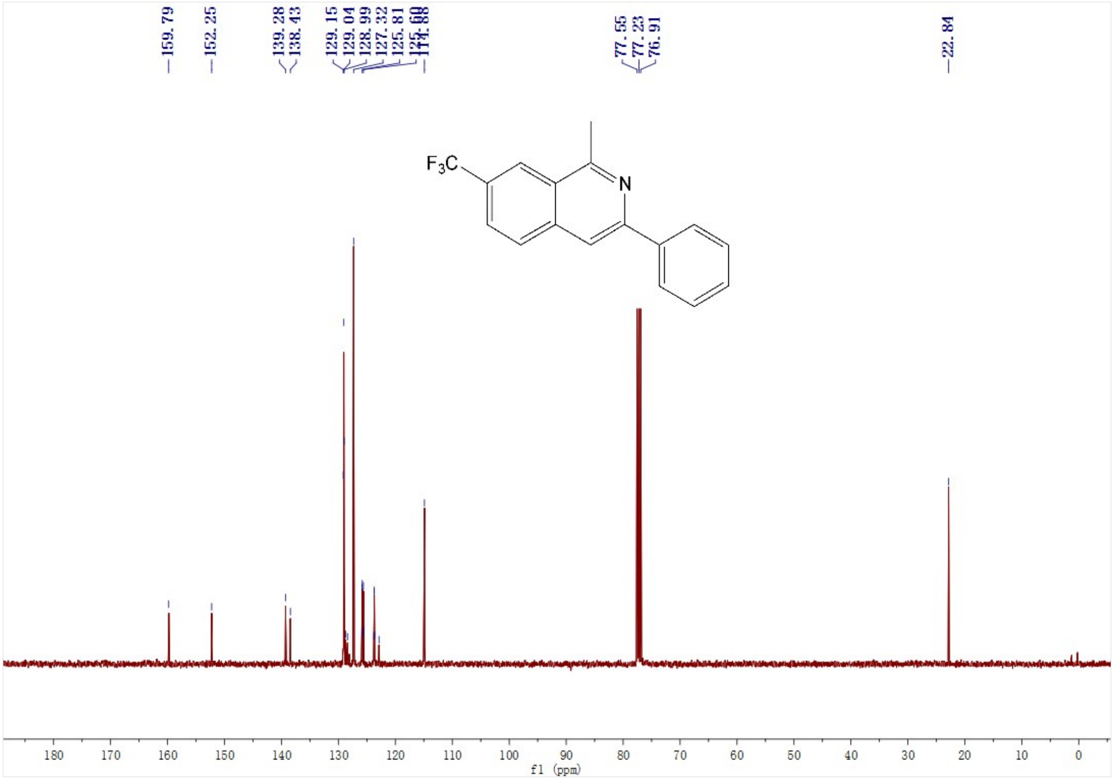

^{19}F NMR spectra of **3ja**

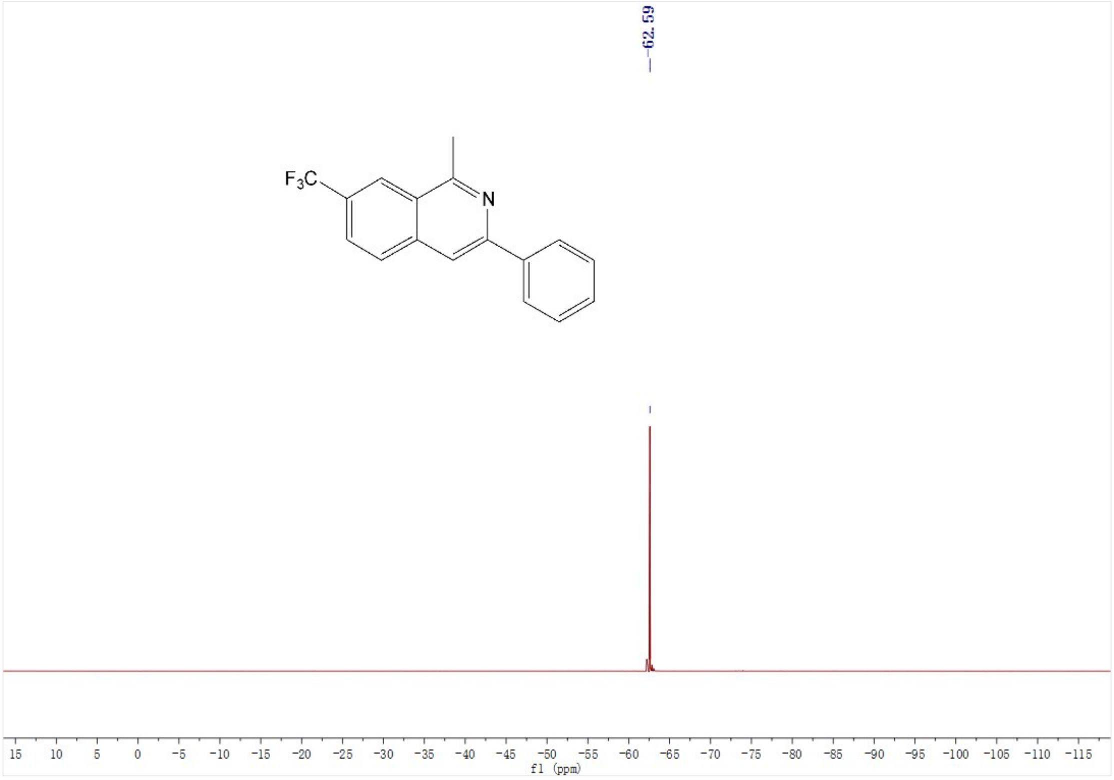

^{1}H NMR and ^{13}C NMR spectra of **3ka**

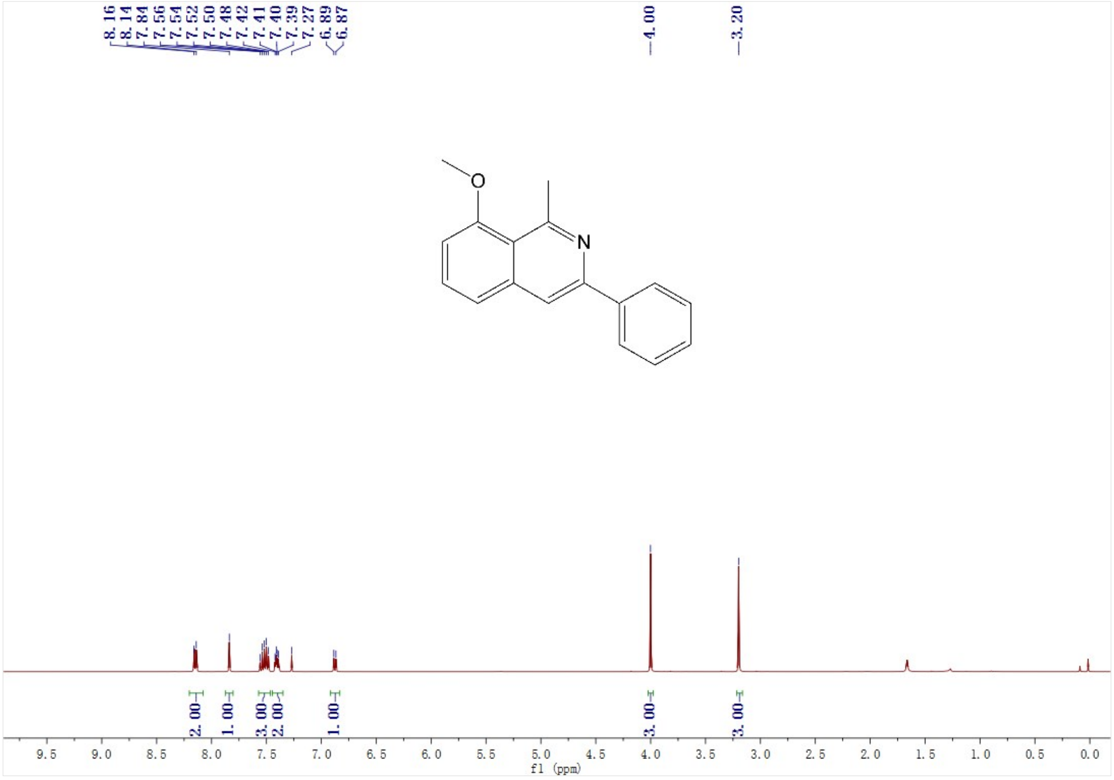

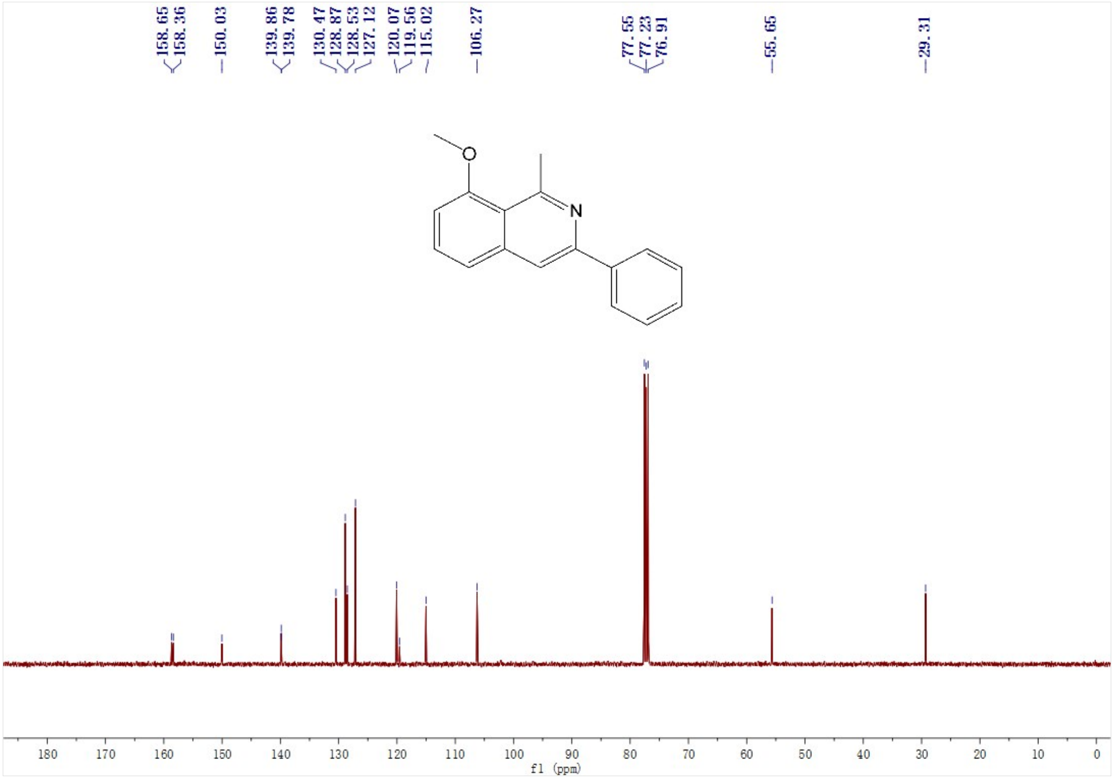

^{1}H NMR and ^{13}C NMR spectra of **3la**

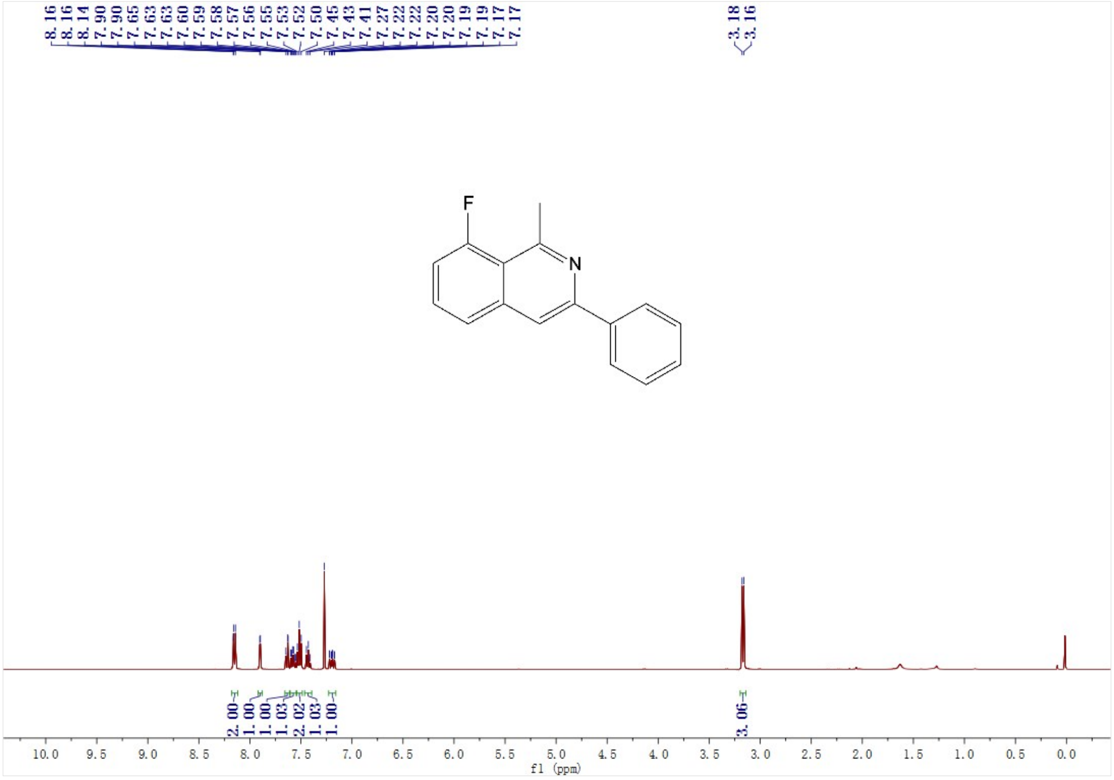

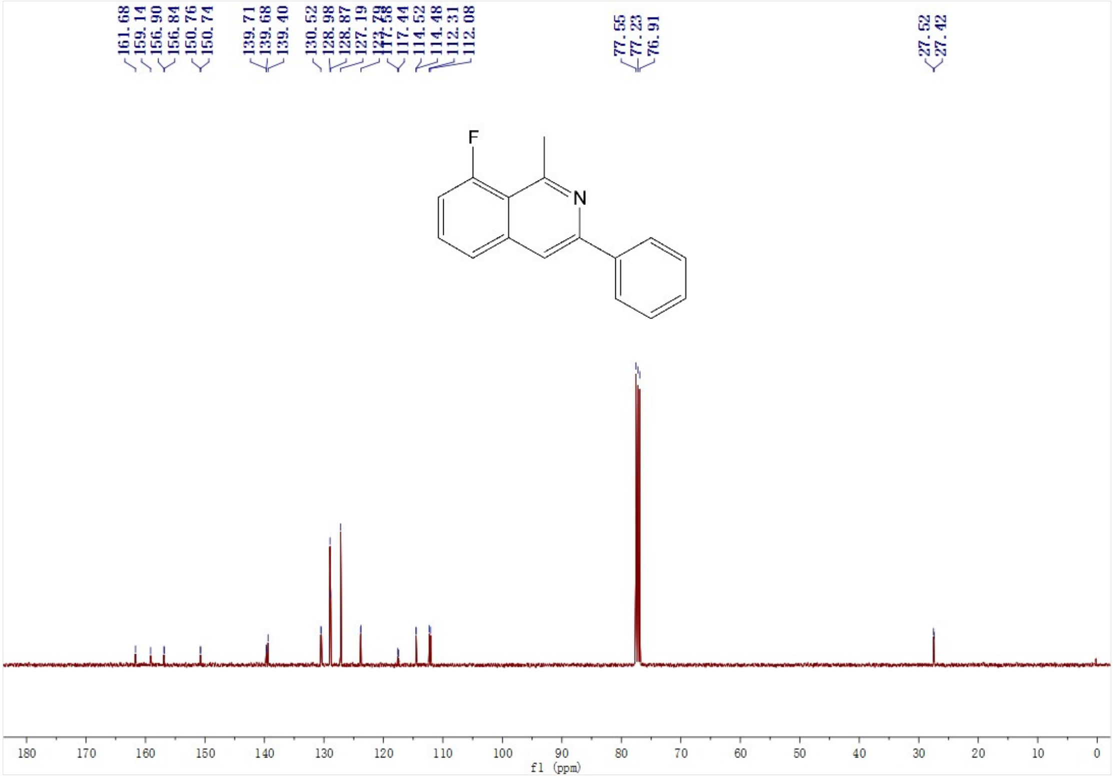

^{19}F NMR spectra of **3la**

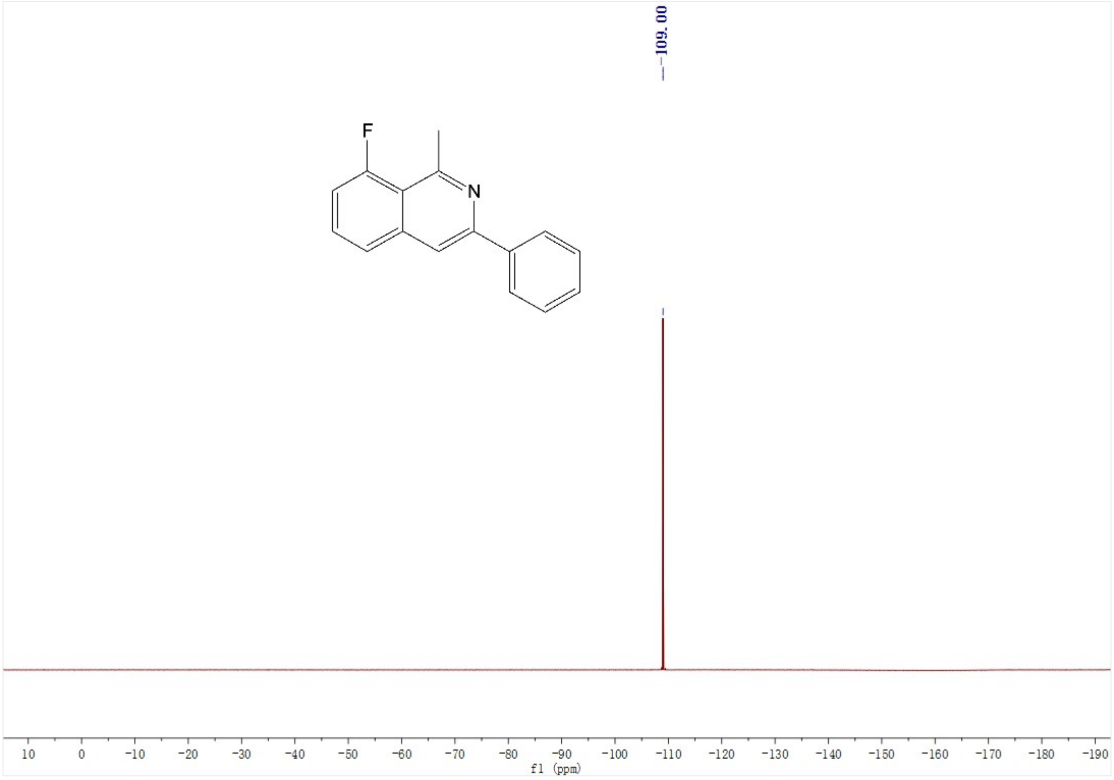

^{1}H NMR and ^{13}C NMR spectra of **3ma**

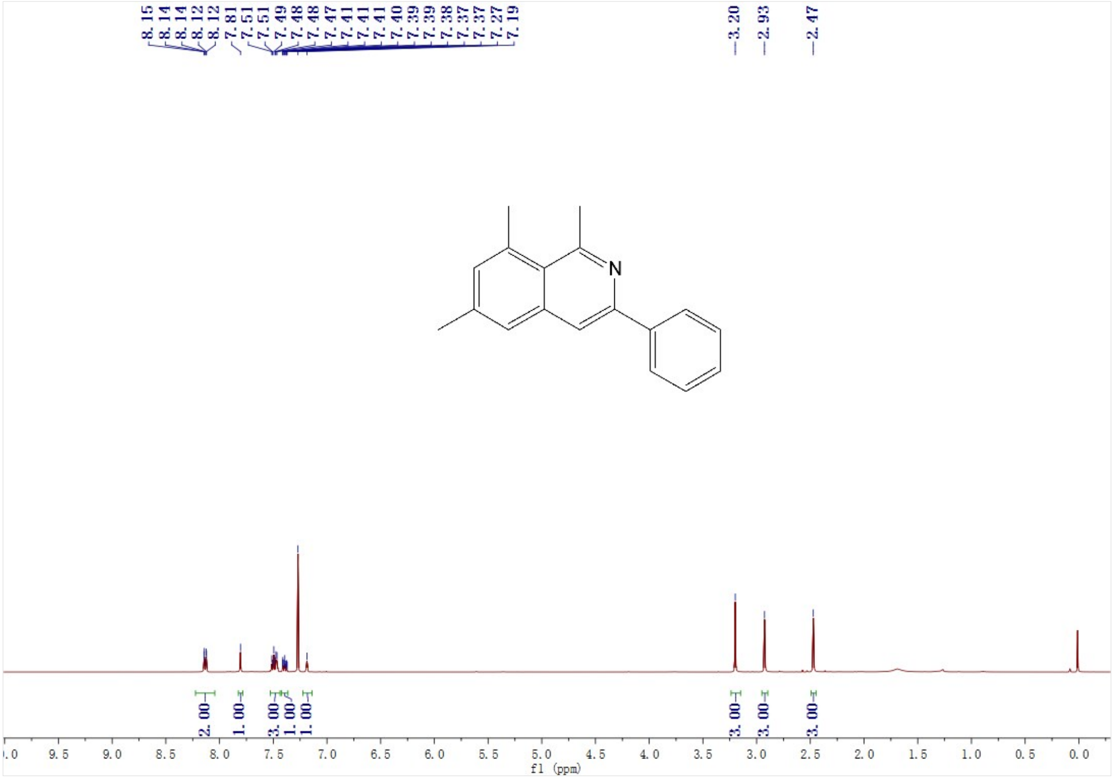

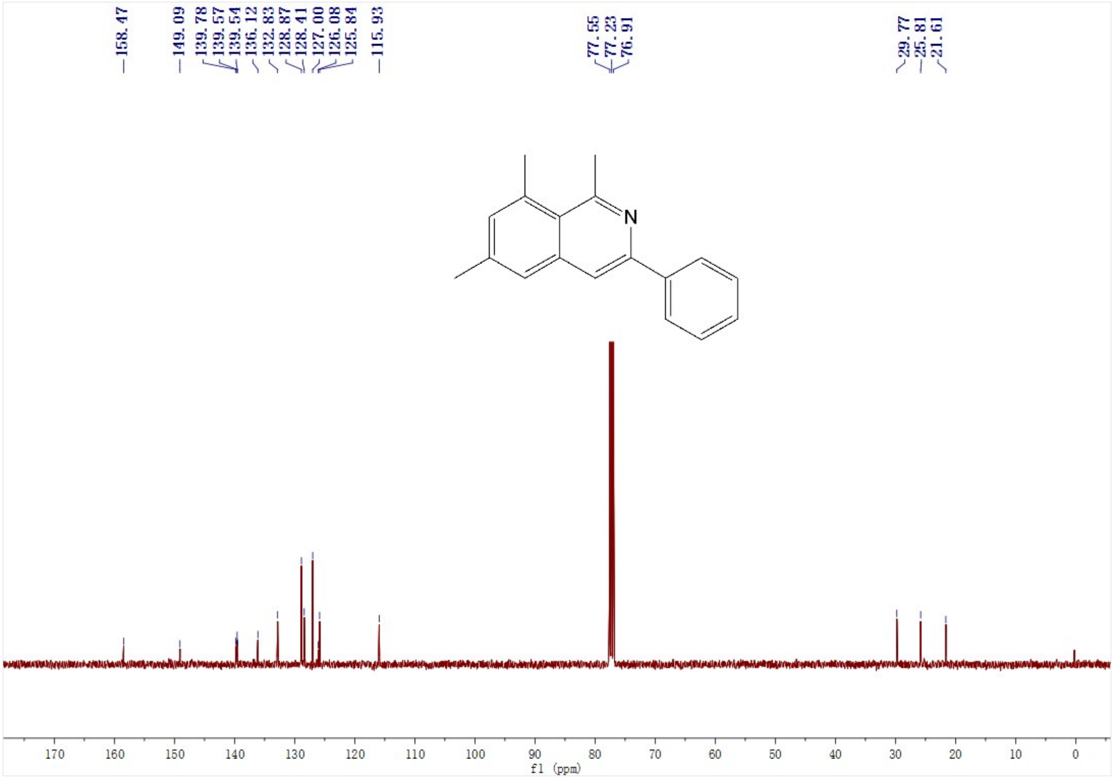

^{1}H NMR and ^{13}C NMR spectra of **3na**

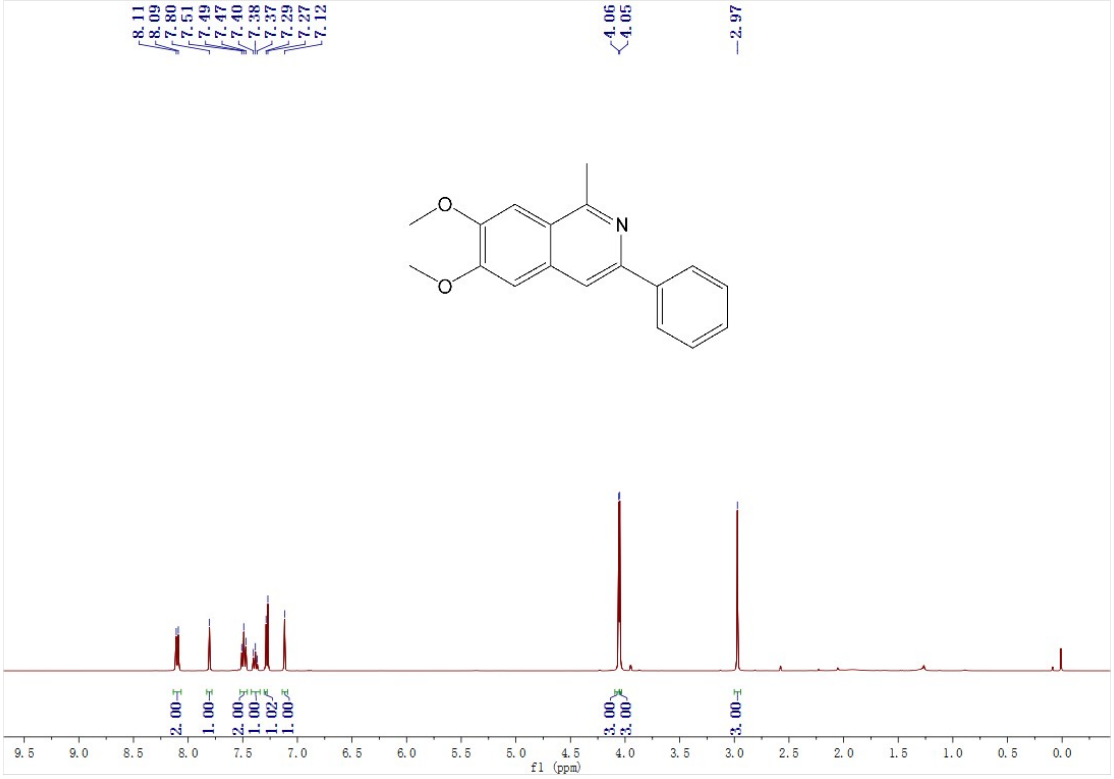

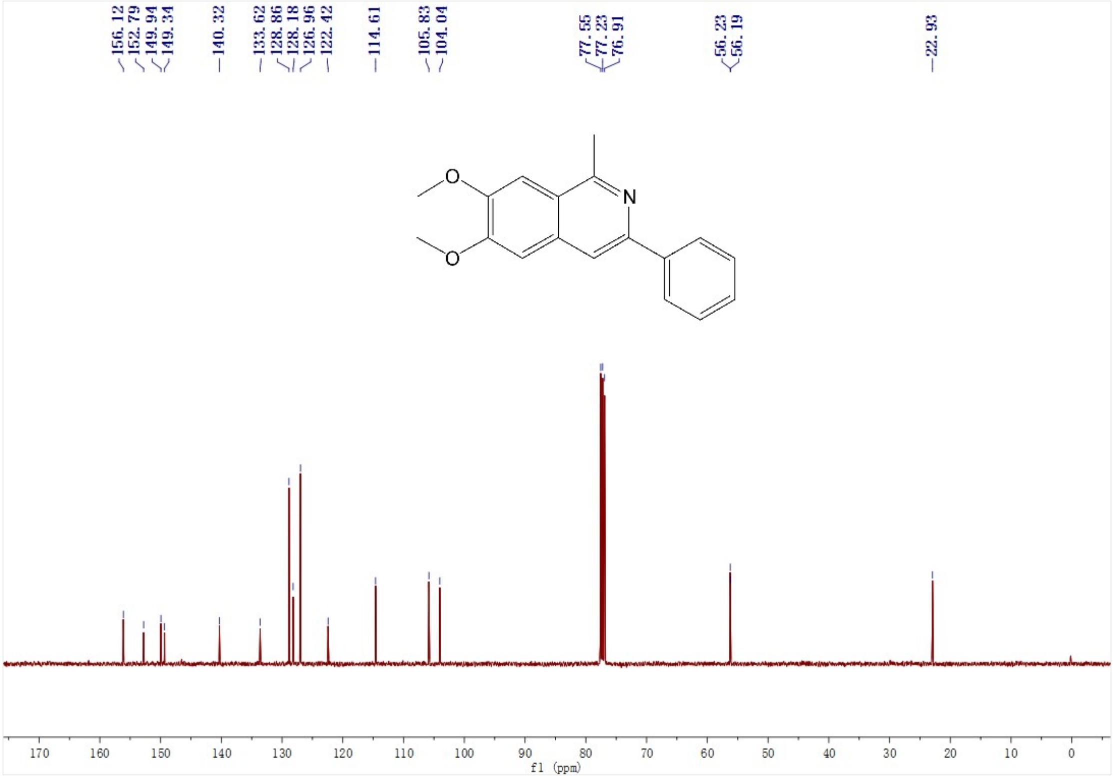

^{1}H NMR and ^{13}C NMR spectra of **3oa**

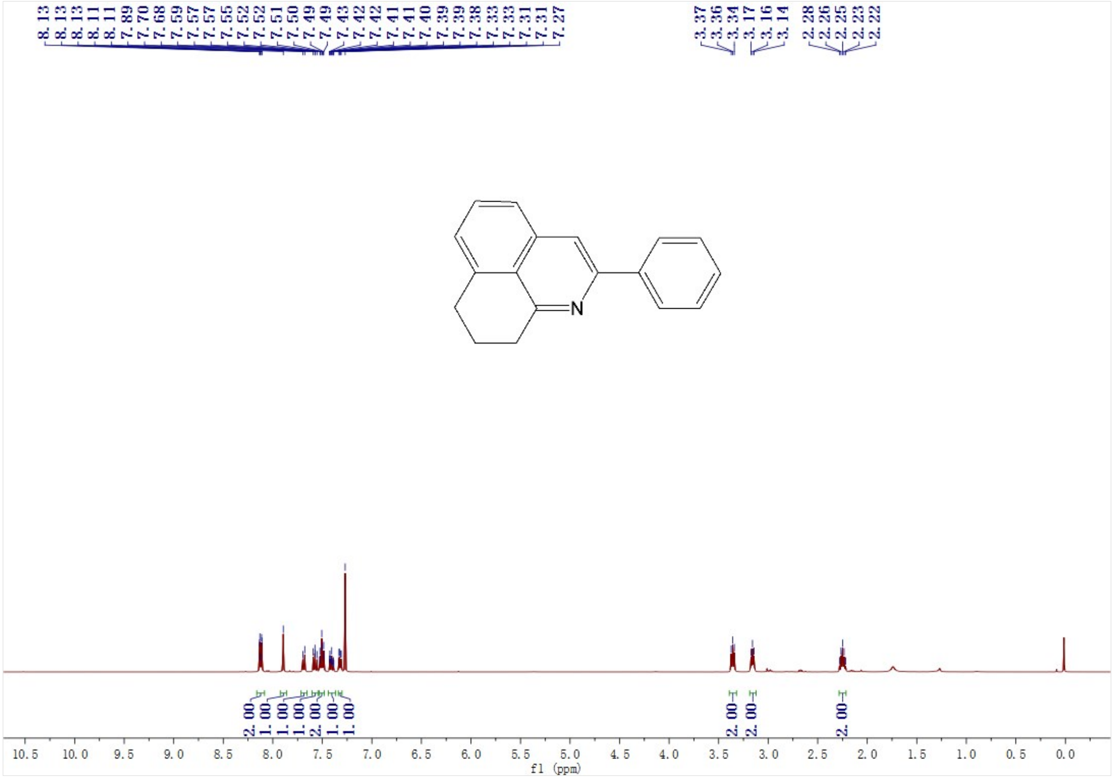

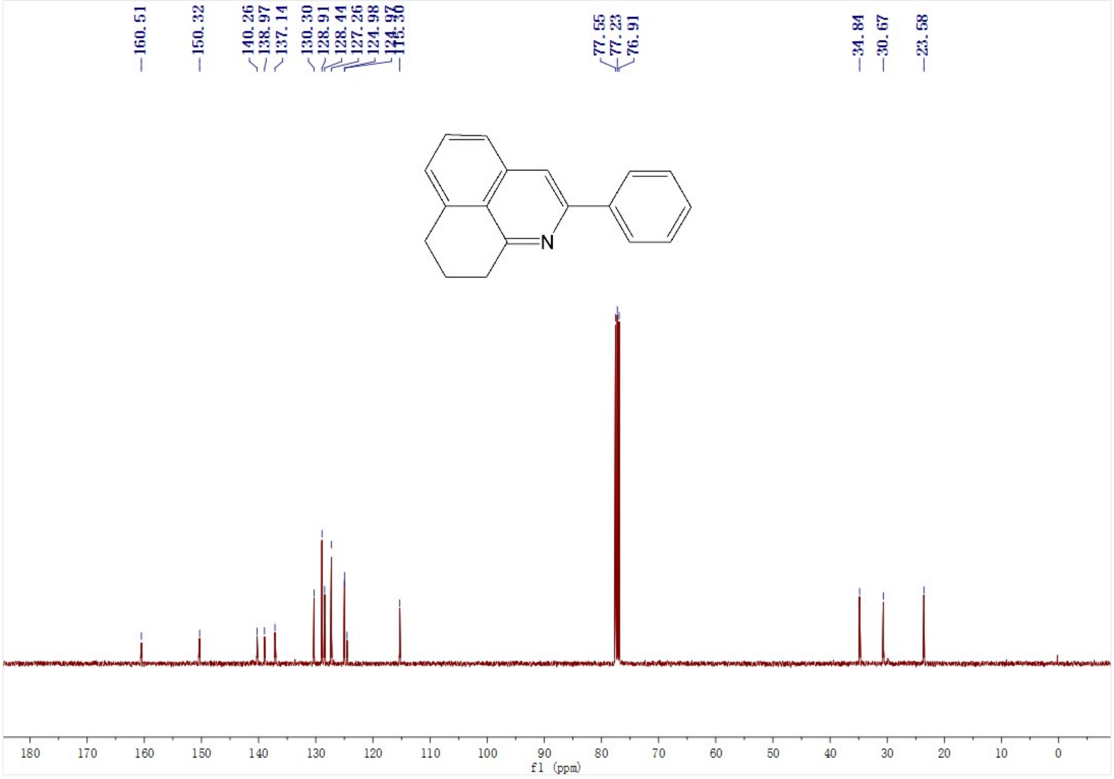

^{1}H NMR and ^{13}C NMR spectra of **3pa**

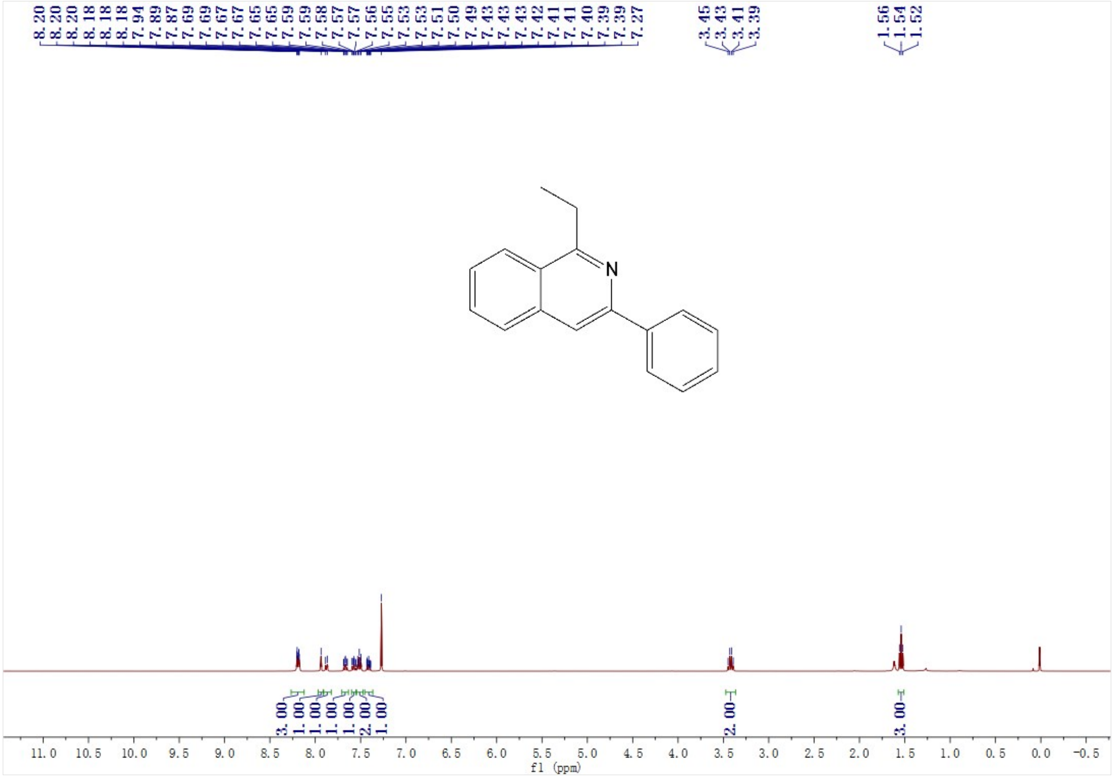

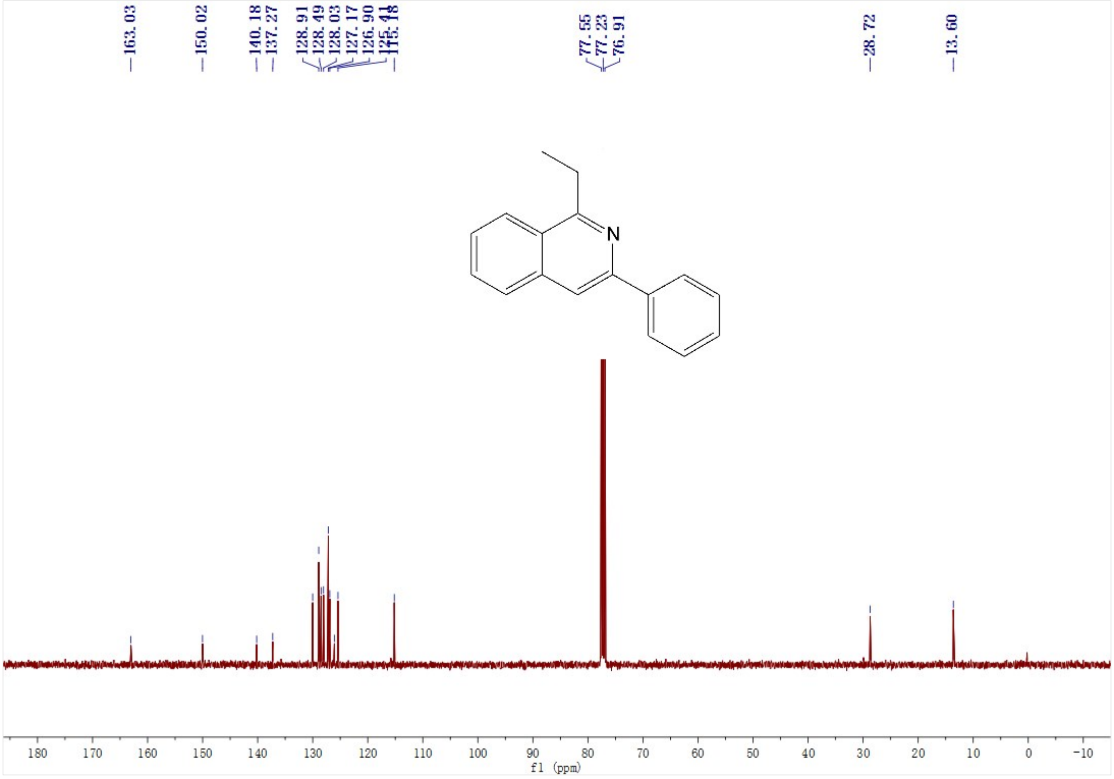

^{1}H NMR and ^{13}C NMR spectra of **3qa**

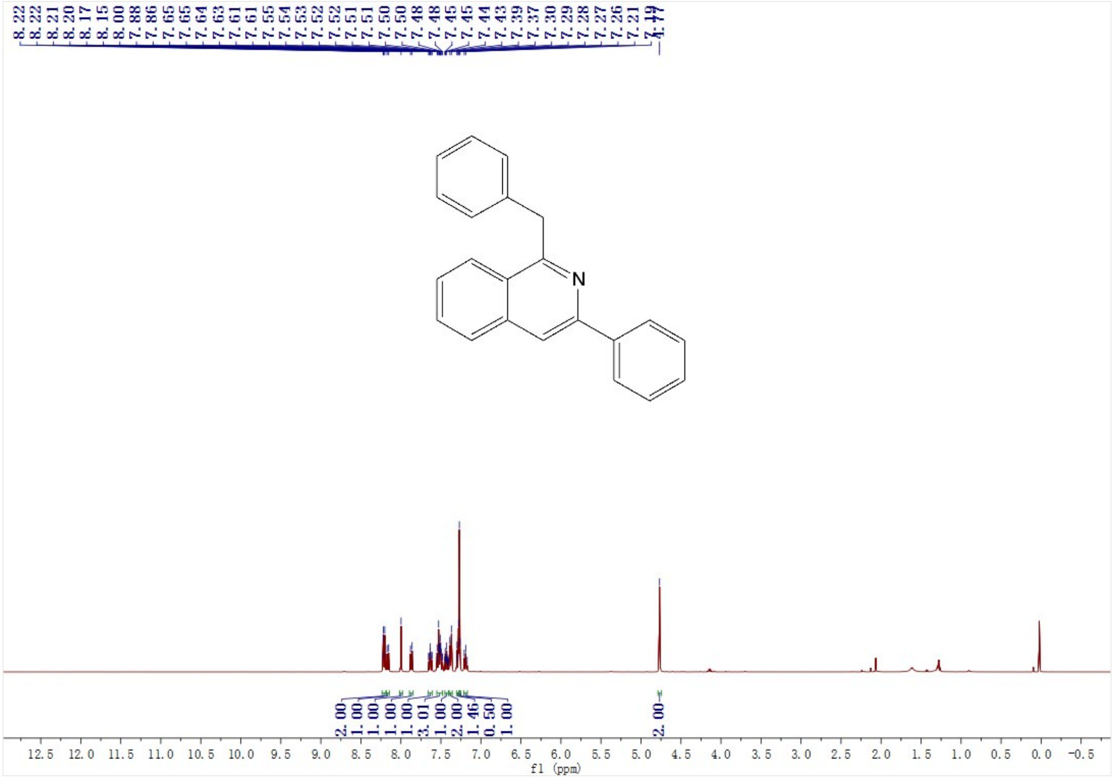

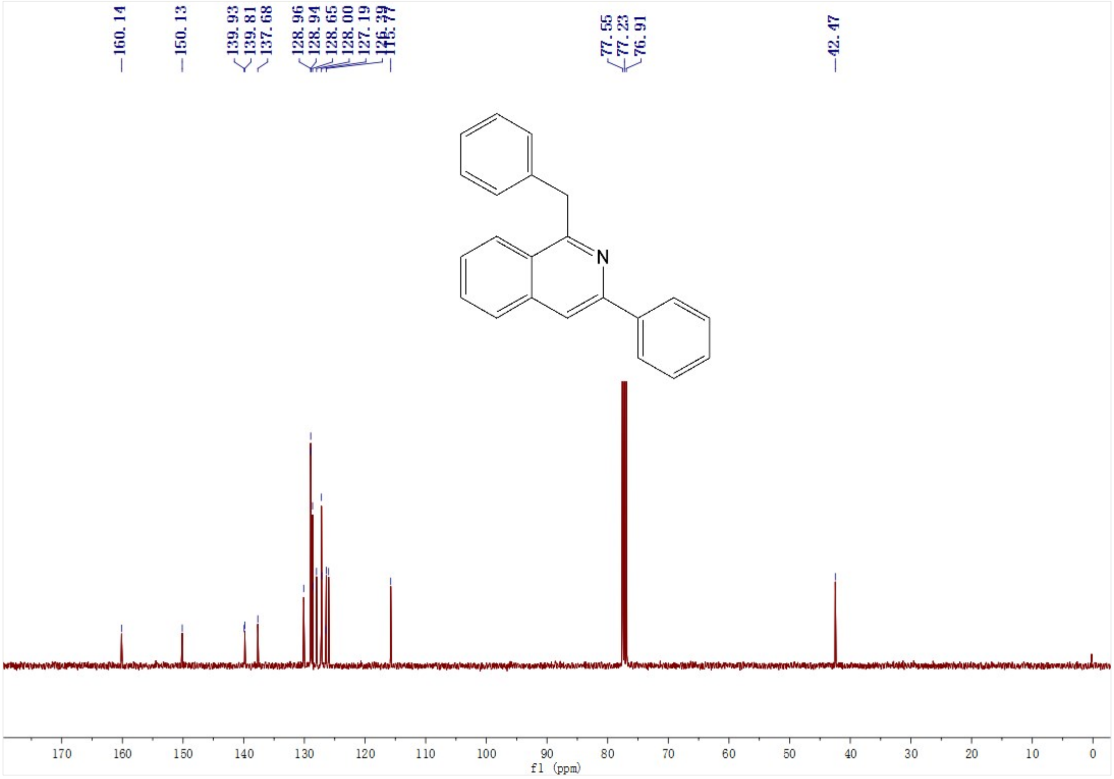

^{1}H NMR and ^{13}C NMR spectra of **3ab**

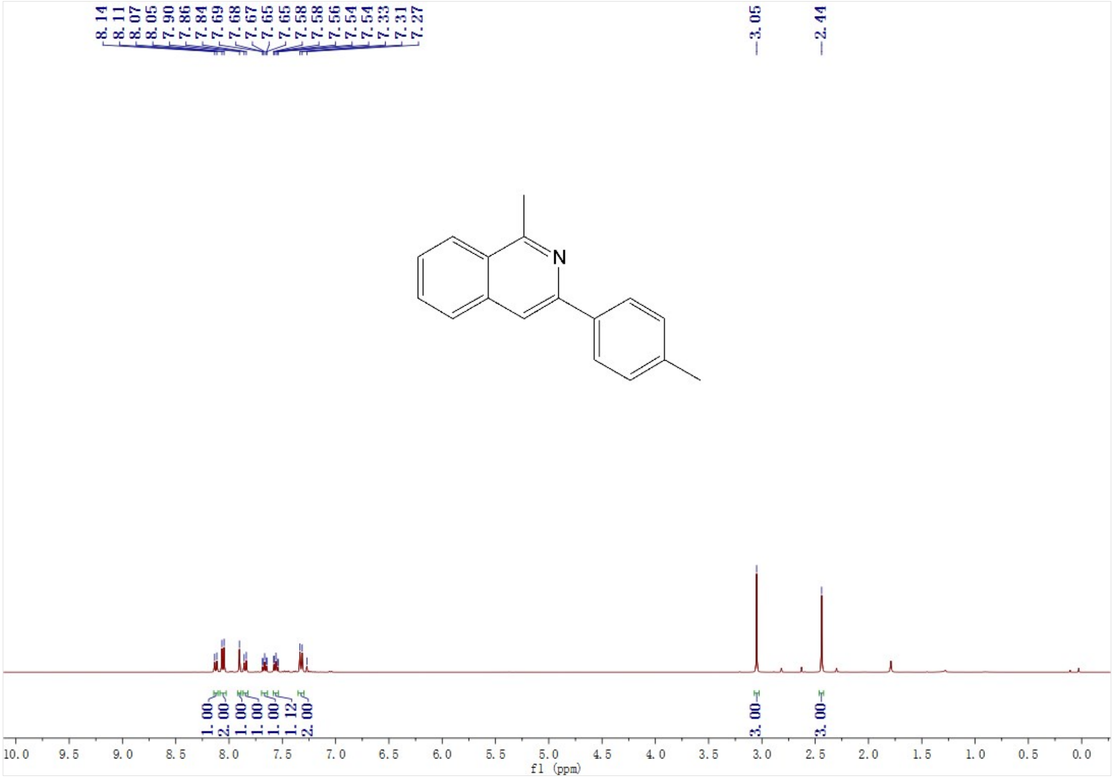

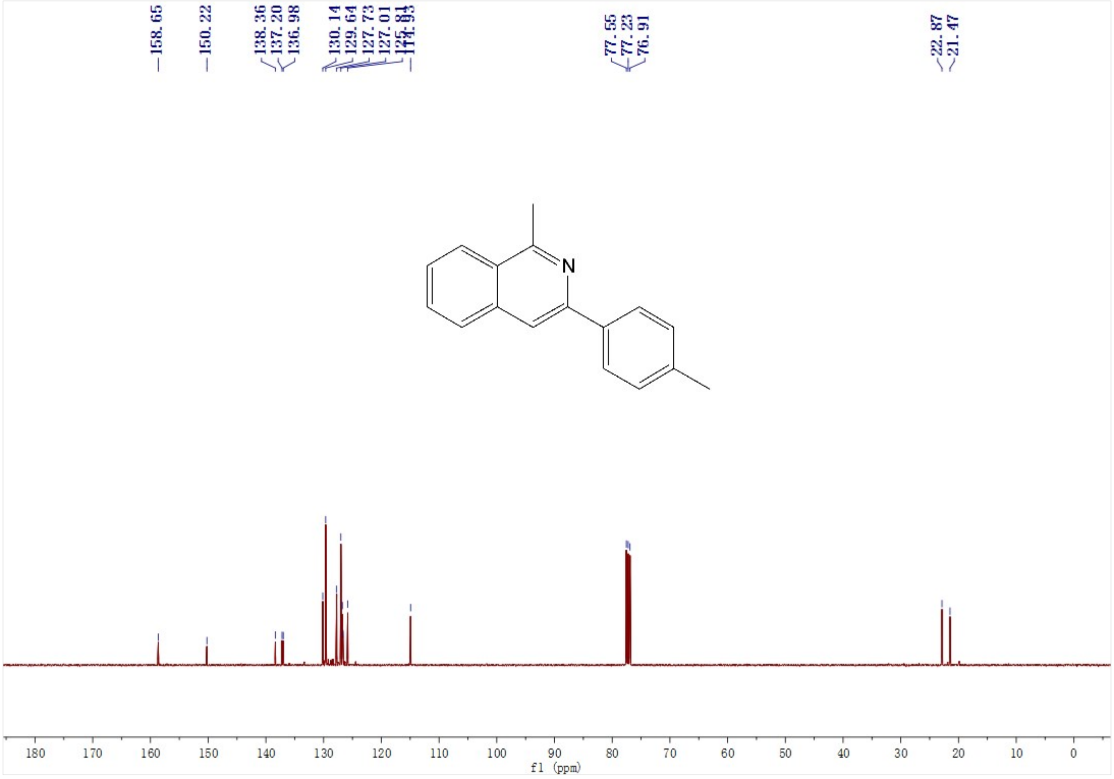

^{1}H NMR and ^{13}C NMR spectra of **3ac**

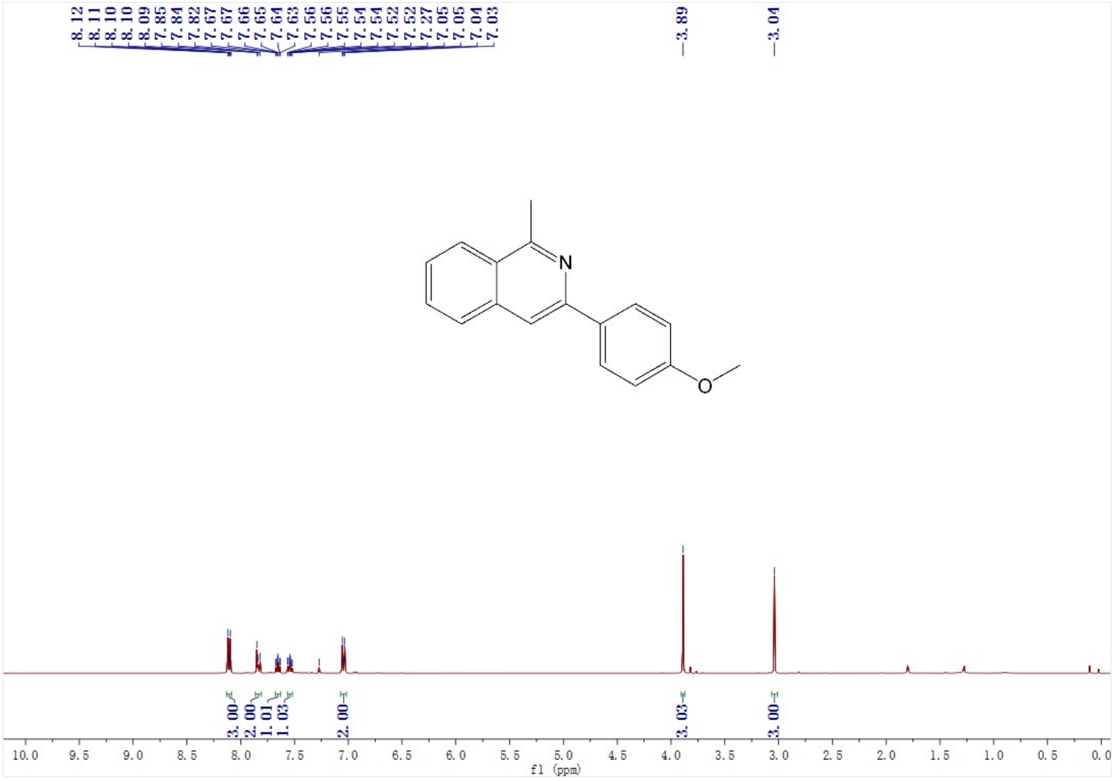

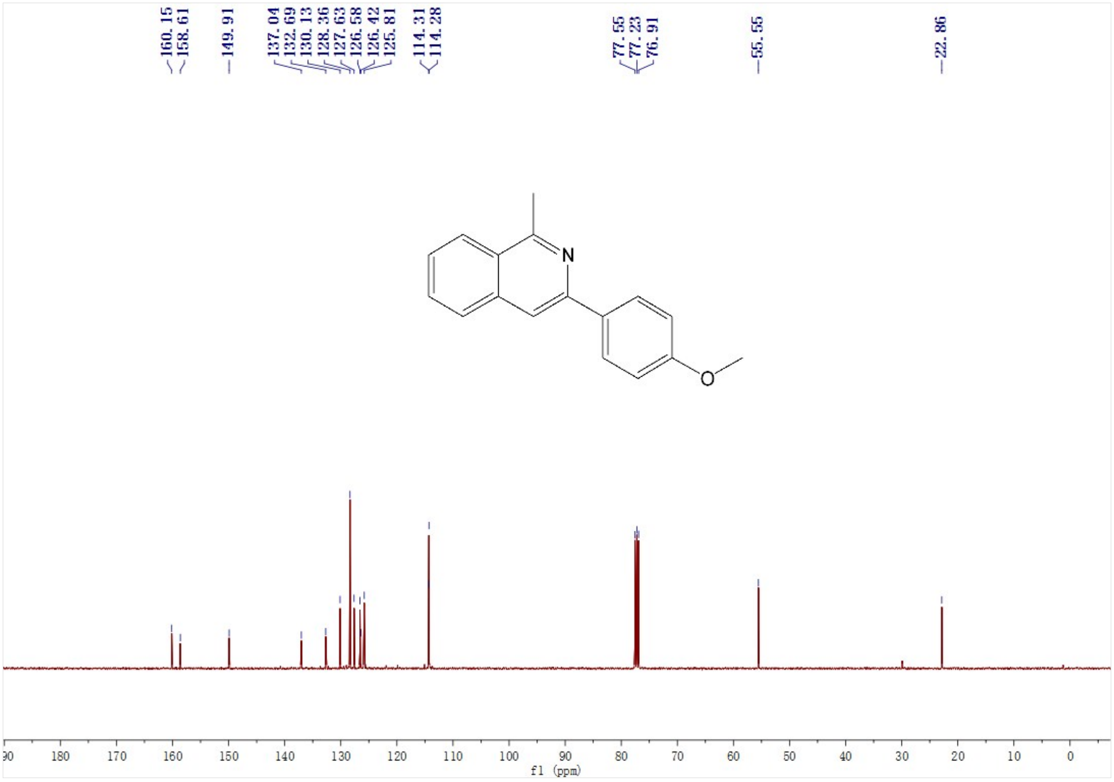

^{1}H NMR and ^{13}C NMR spectra of **3ad**

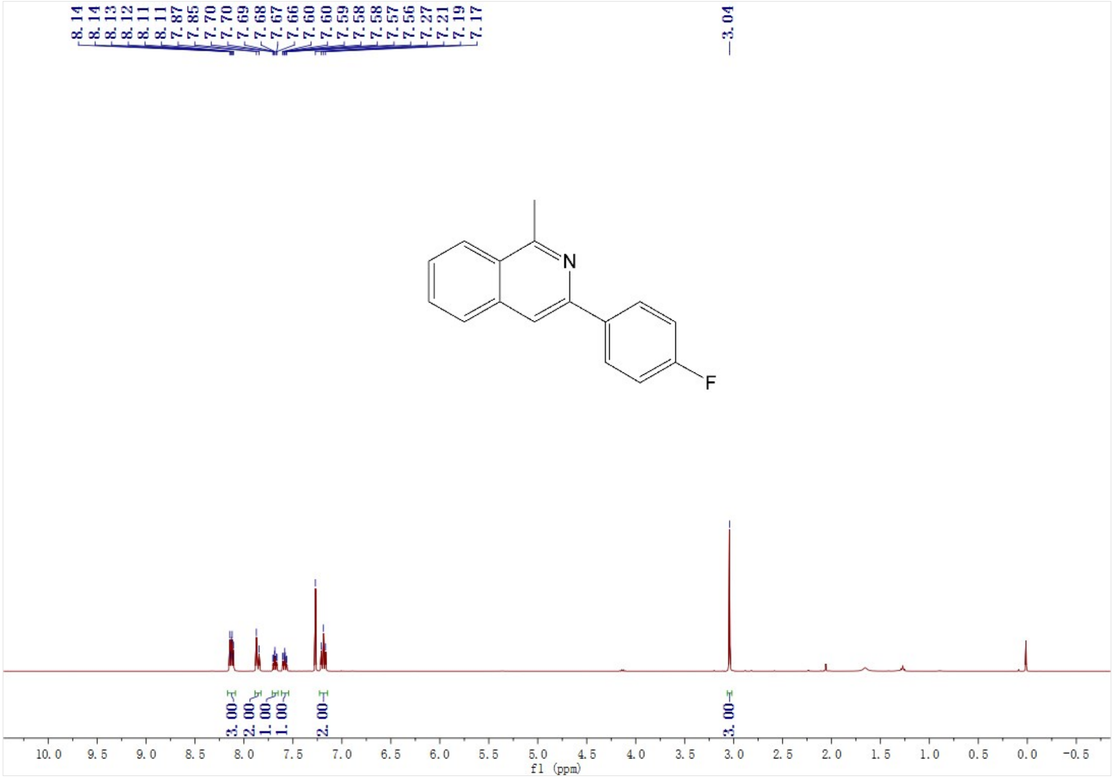

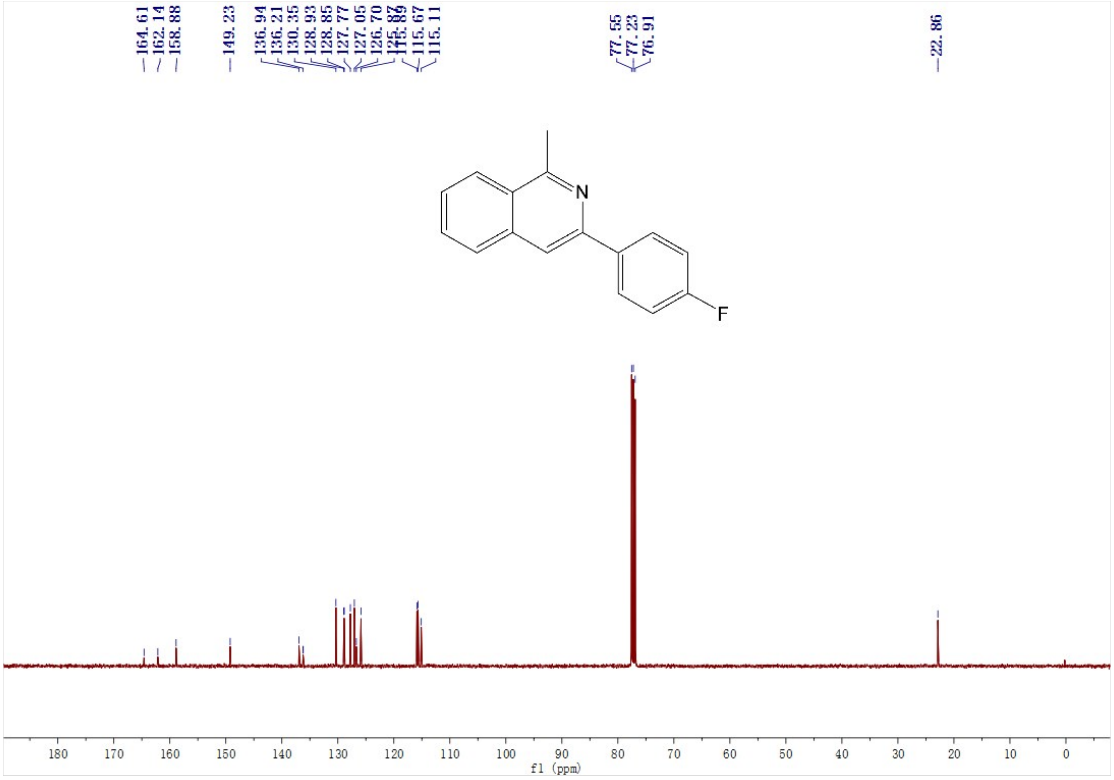

^{19}F NMR spectra of **3ad**

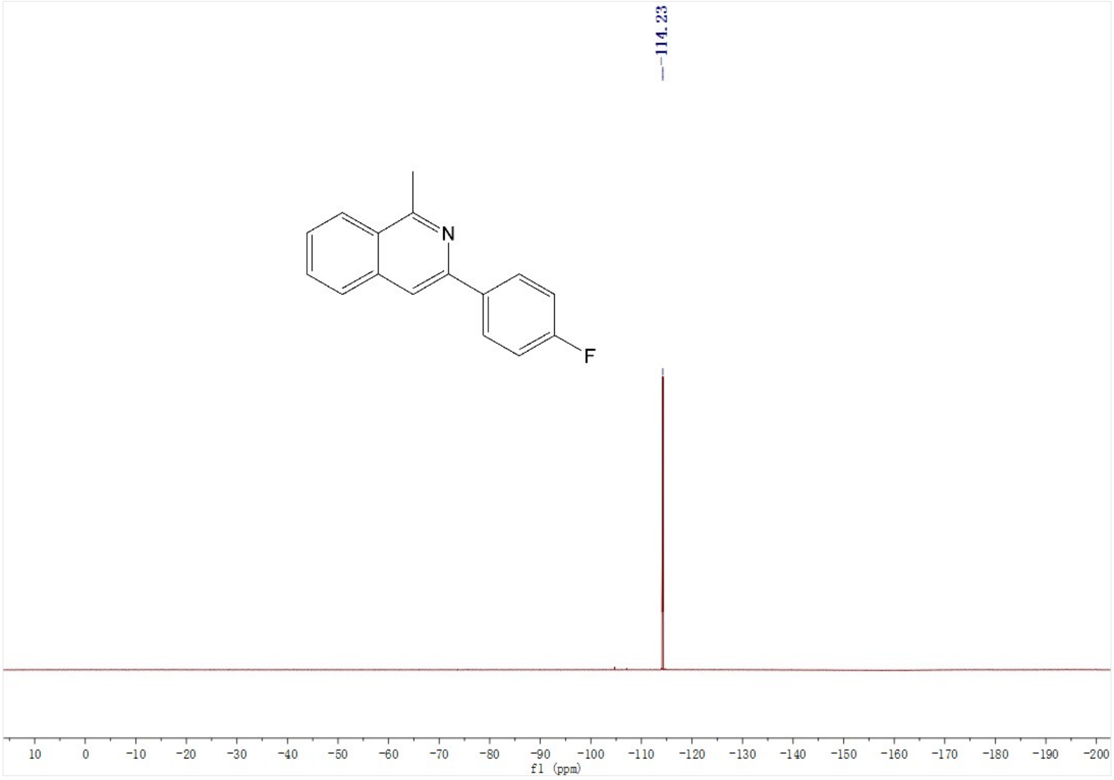

^{1}H NMR and ^{13}C NMR spectra of **3ae**

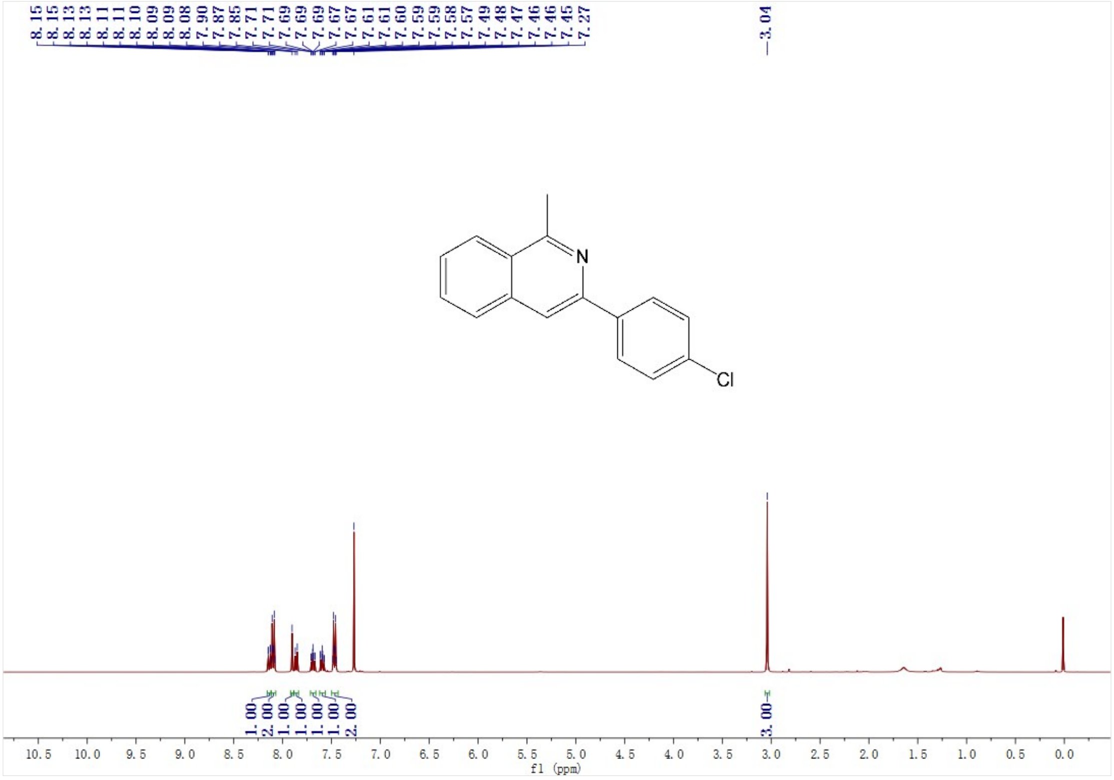

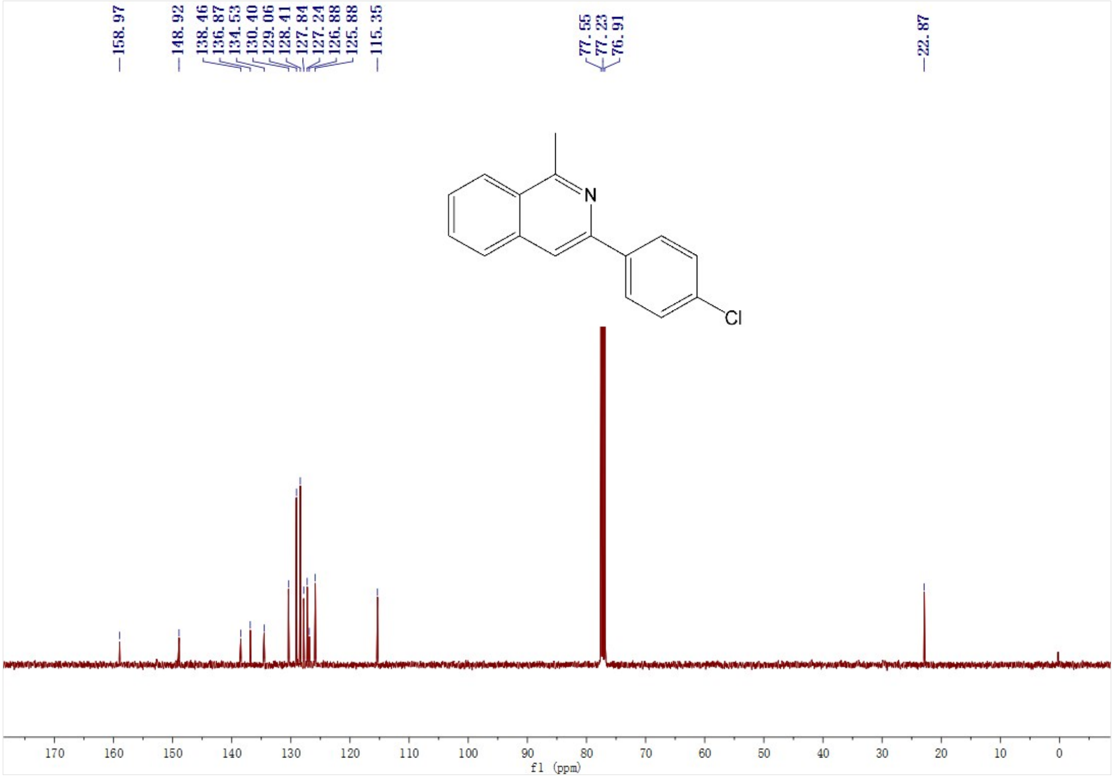

^{1}H NMR and ^{13}C NMR spectra of **3af**

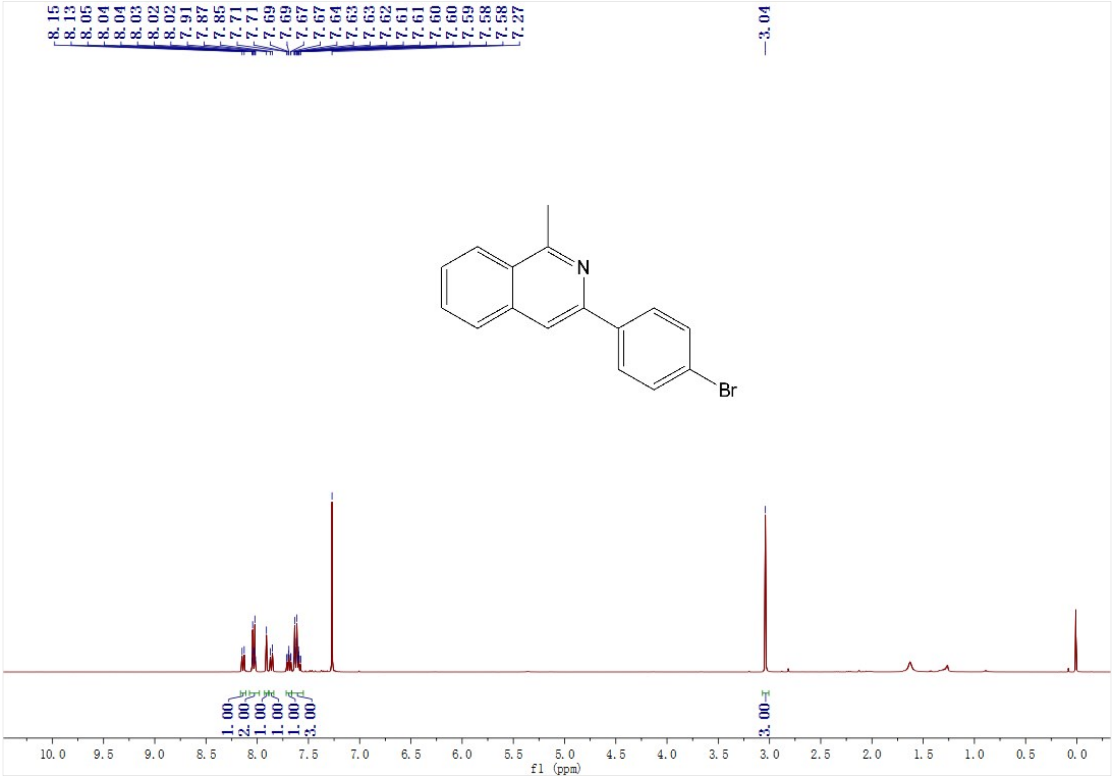

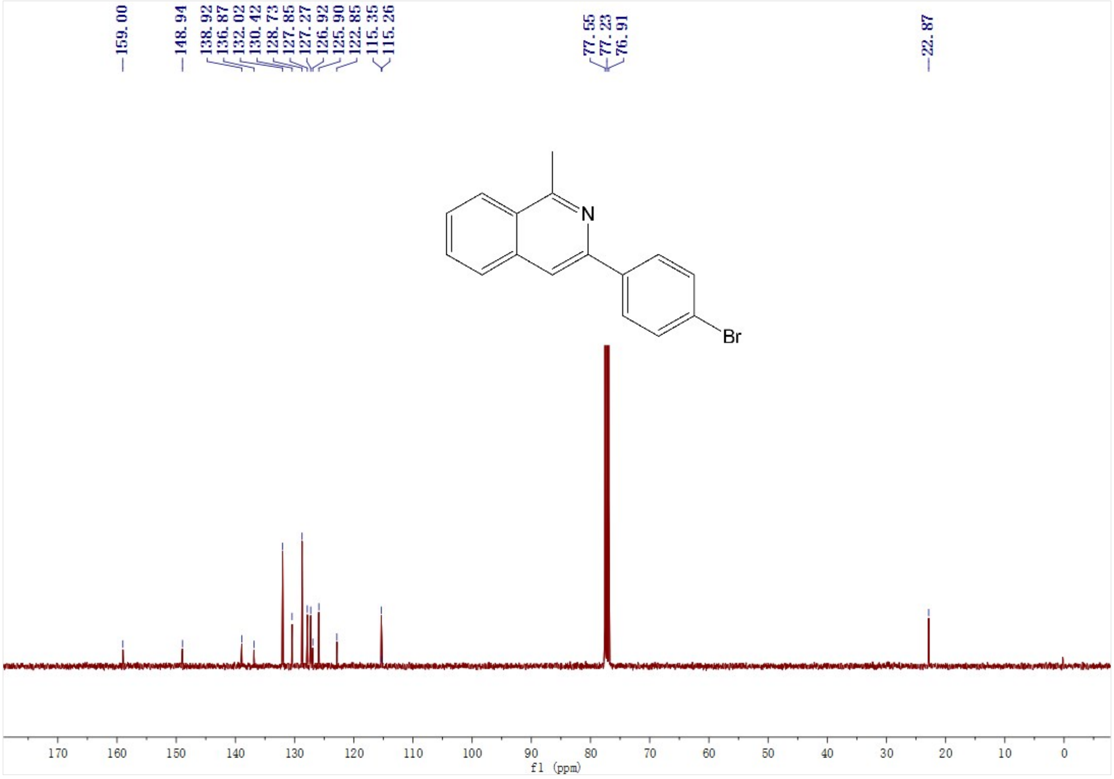

^{1}H NMR and ^{13}C NMR spectra of **3ag**

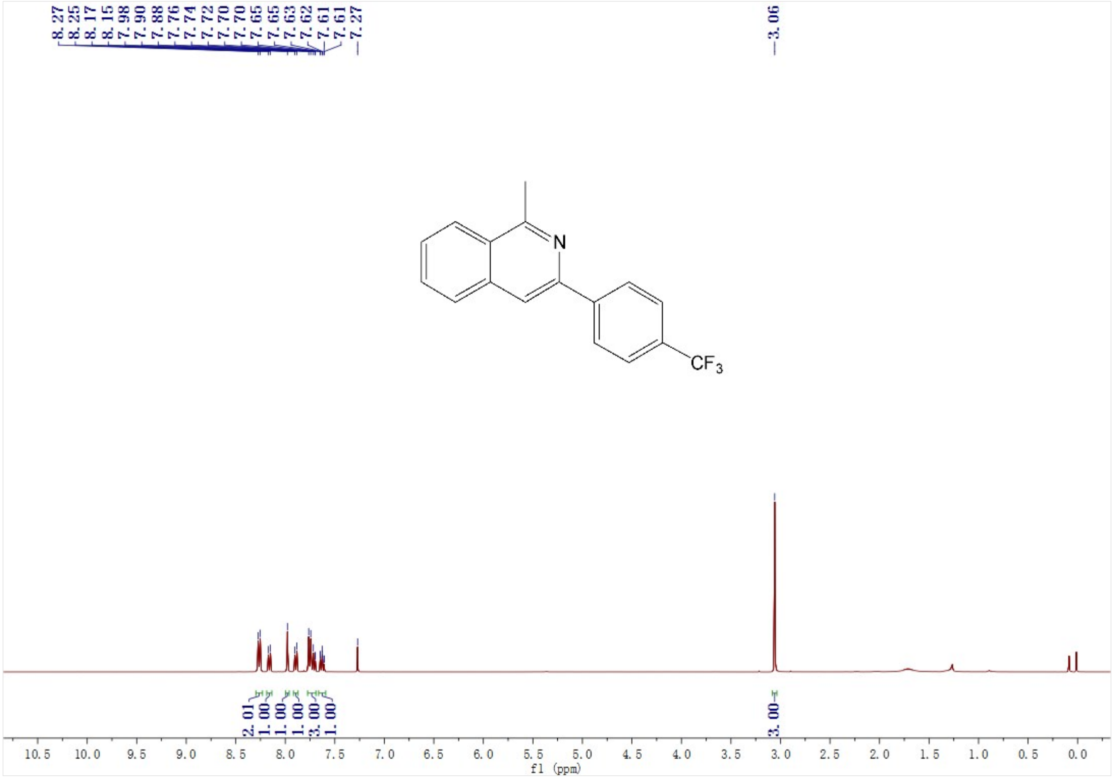

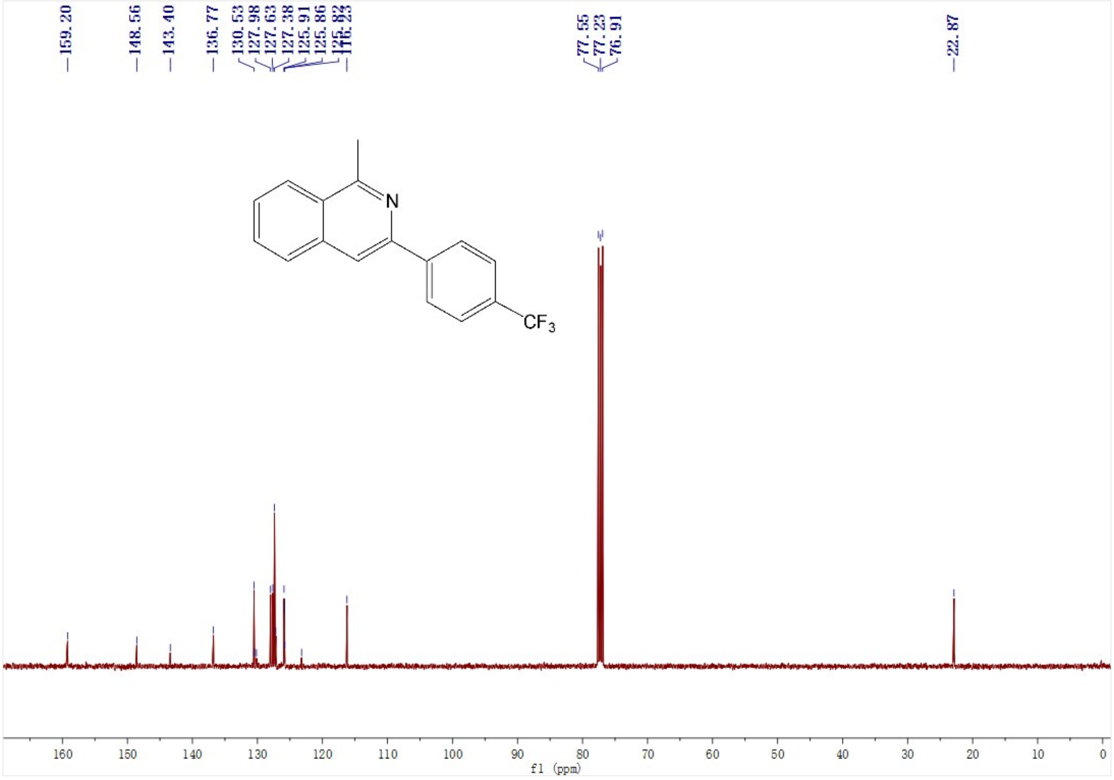

^{19}F NMR spectra of **3ag**

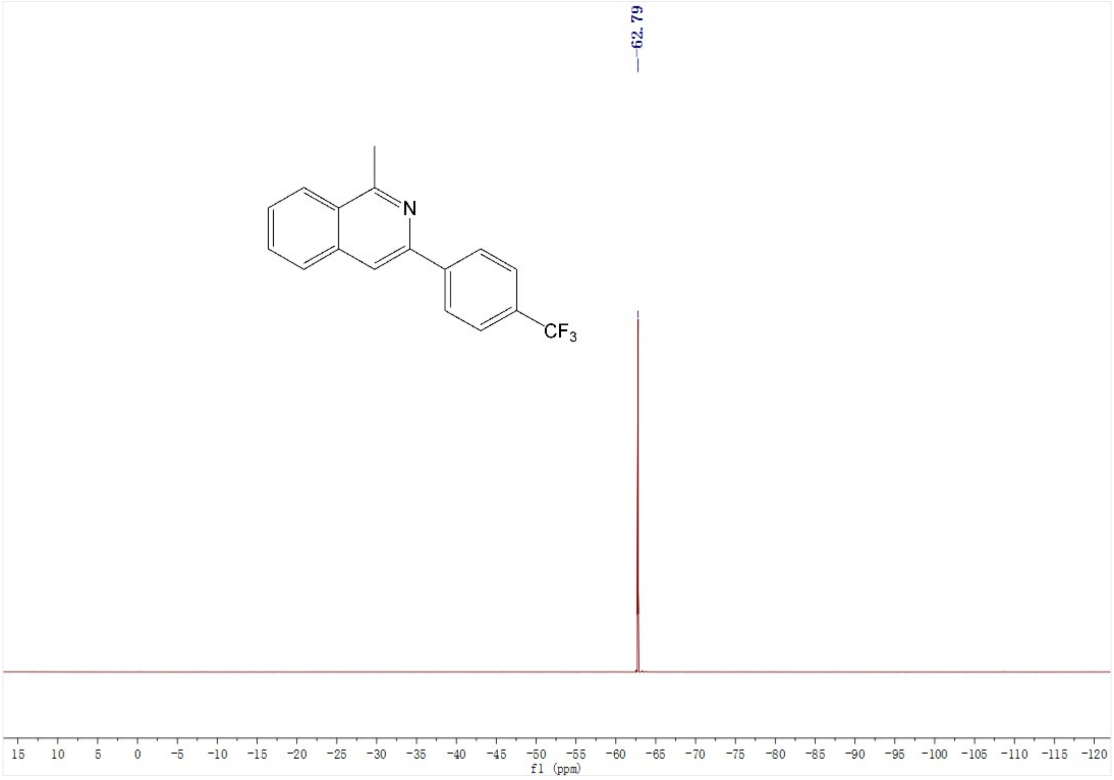

^1H NMR and ^{13}C NMR spectra of **3ah**

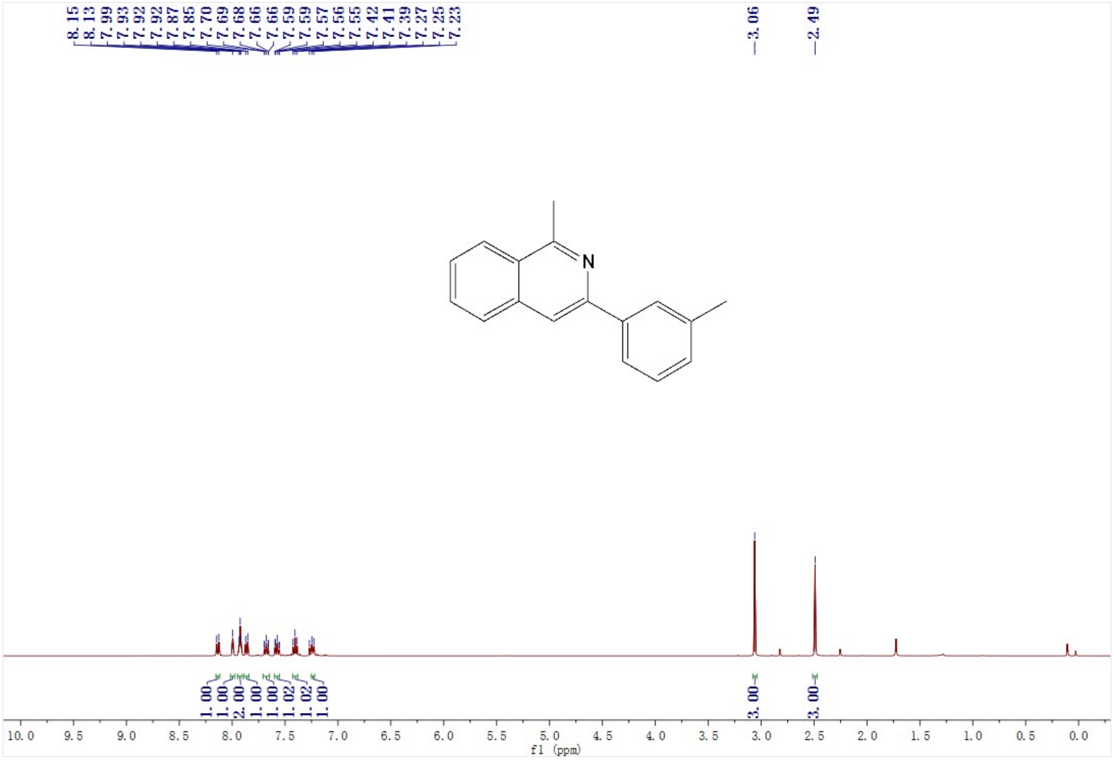

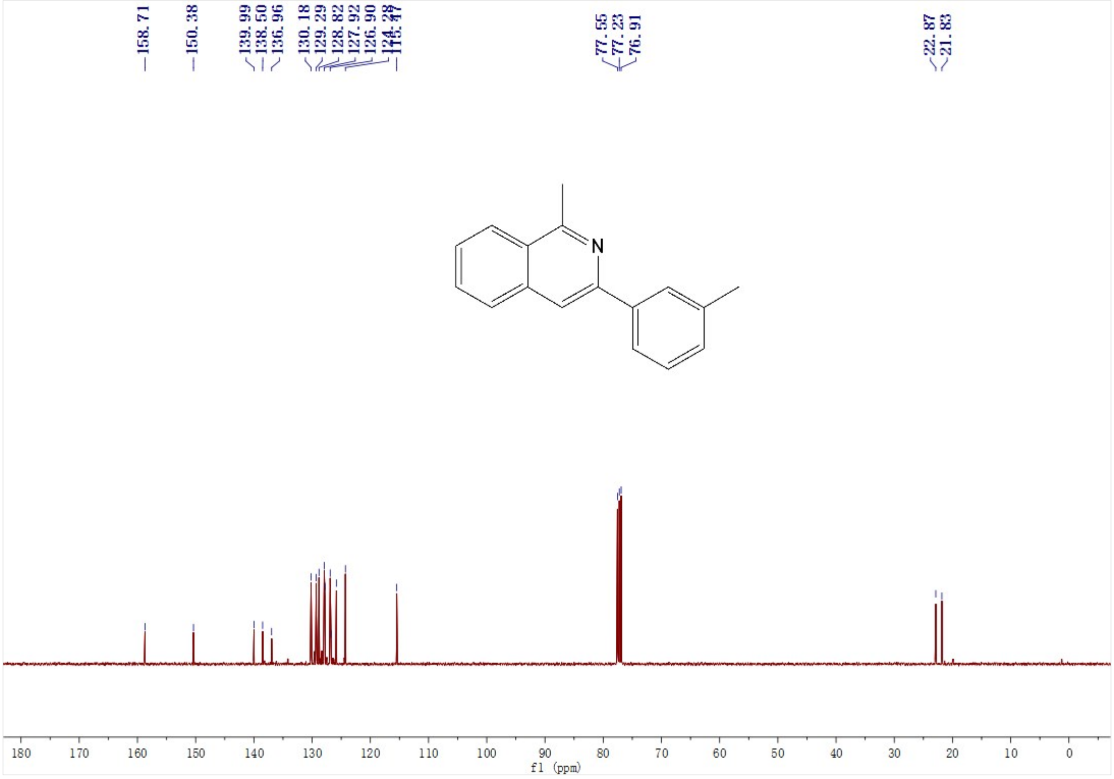

^{1}H NMR and ^{13}C NMR spectra of **3ai**

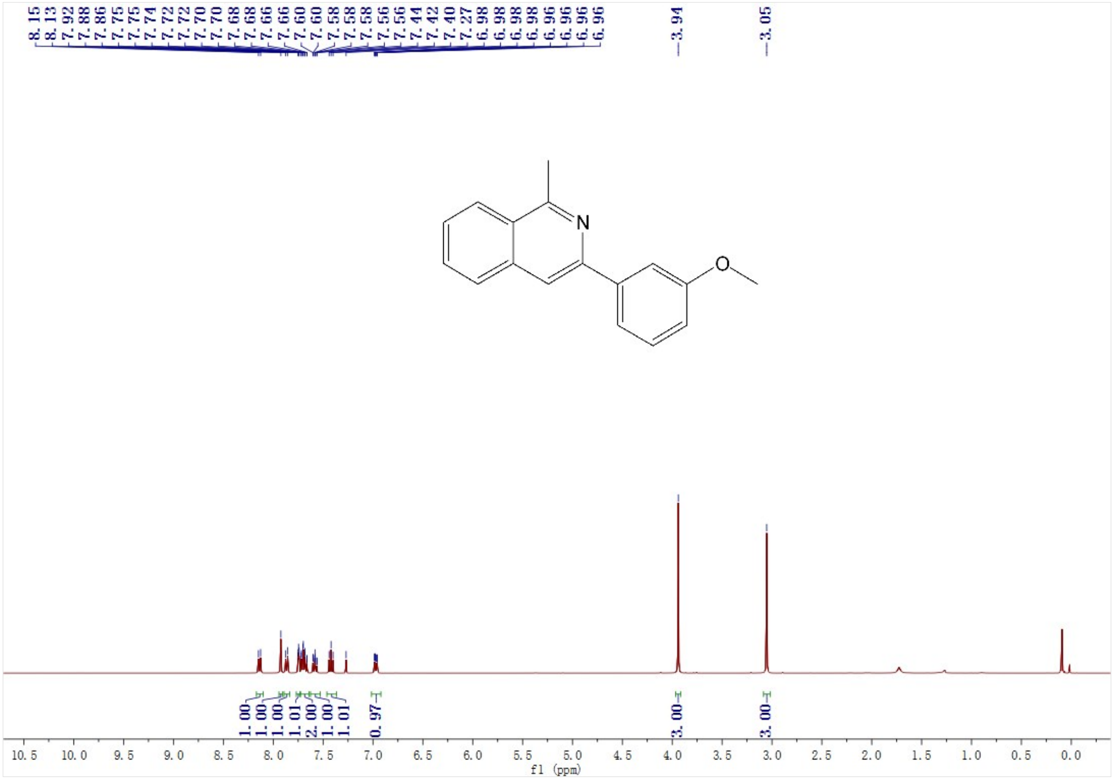

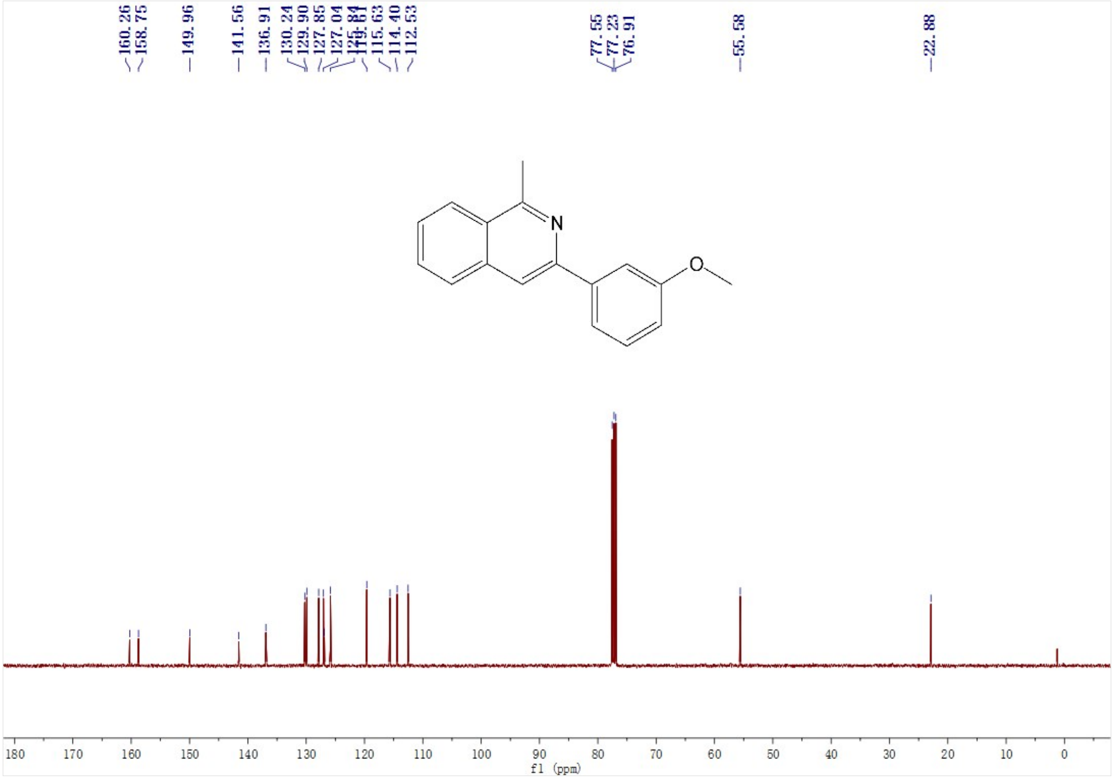

^1H NMR and ^{13}C NMR spectra of **3aj**

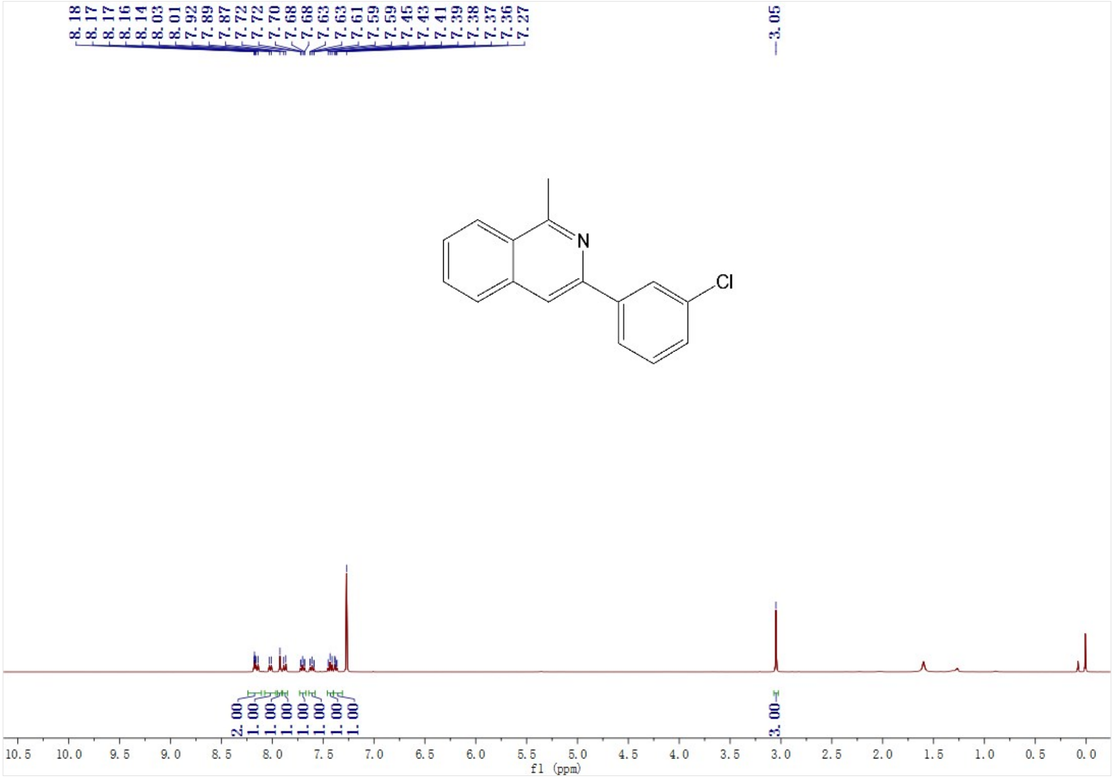

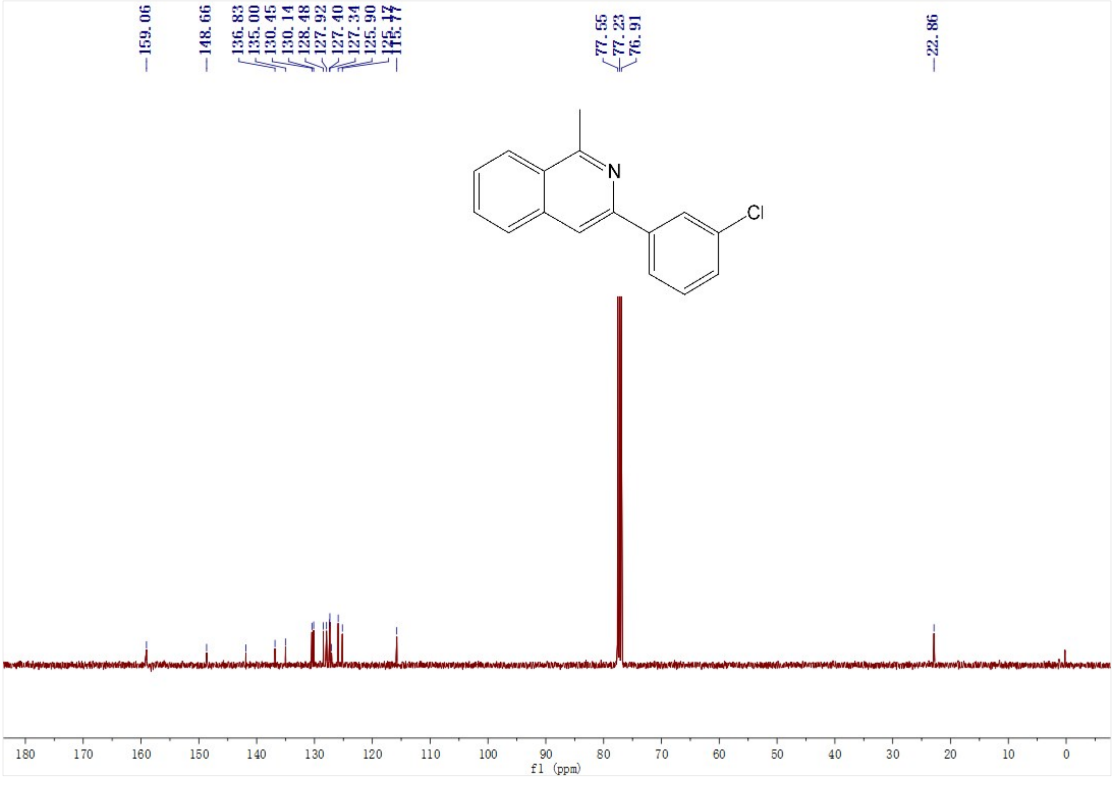

^{1}H NMR and ^{13}C NMR spectra of **3ak**

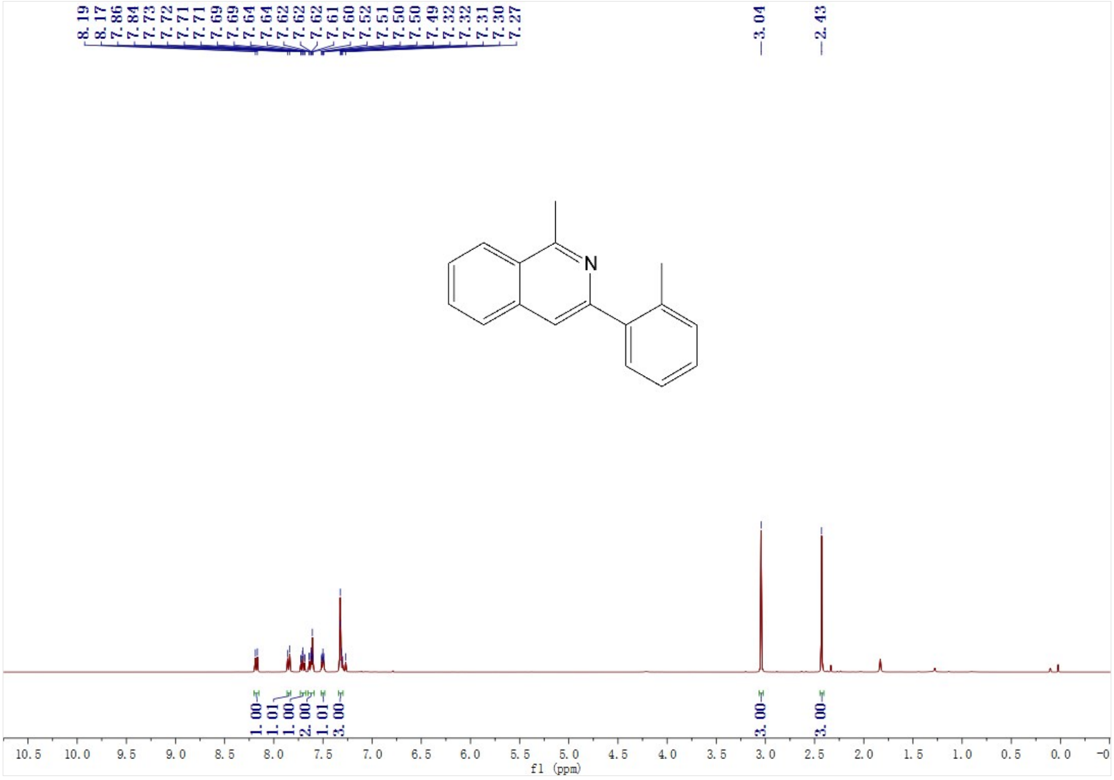

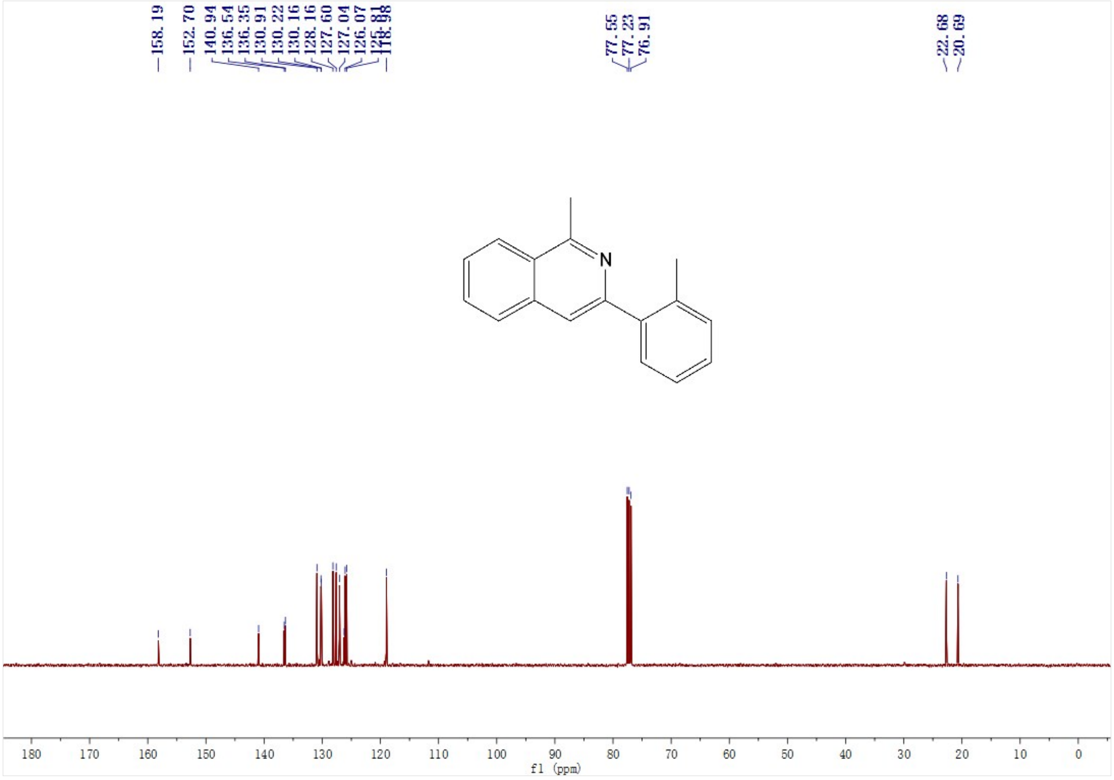

^{1}H NMR and ^{13}C NMR spectra of **3al**

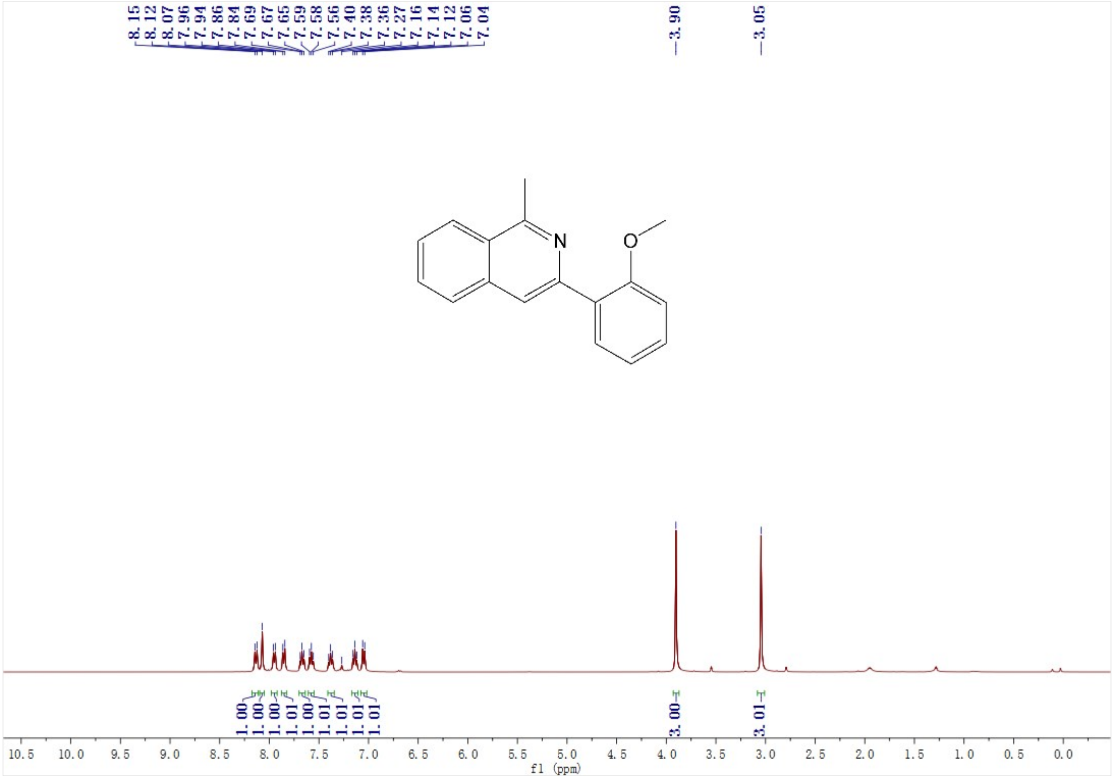

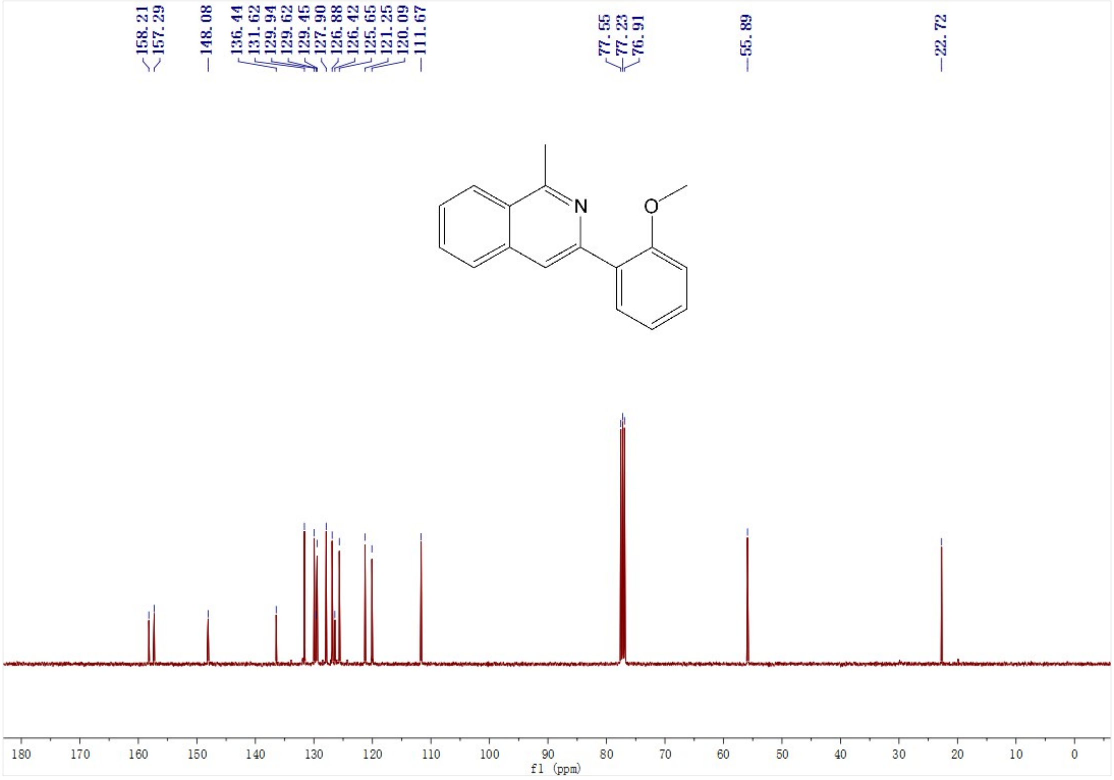

^{1}H NMR and ^{13}C NMR spectra of **3am**

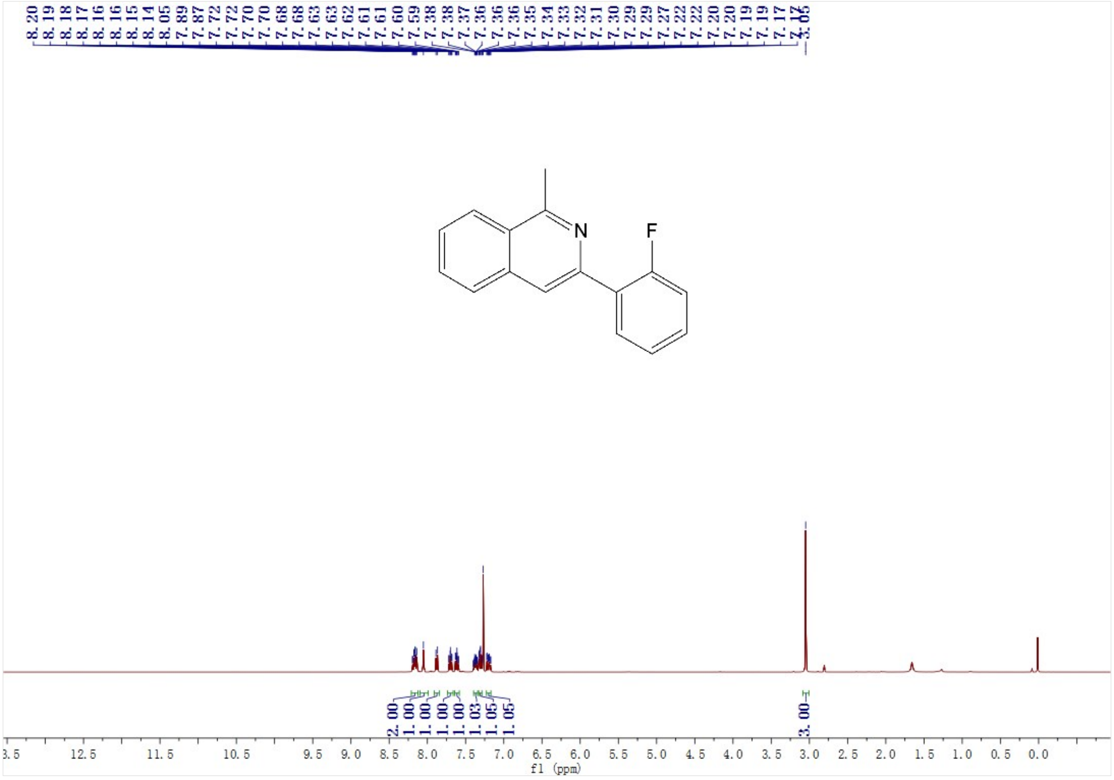

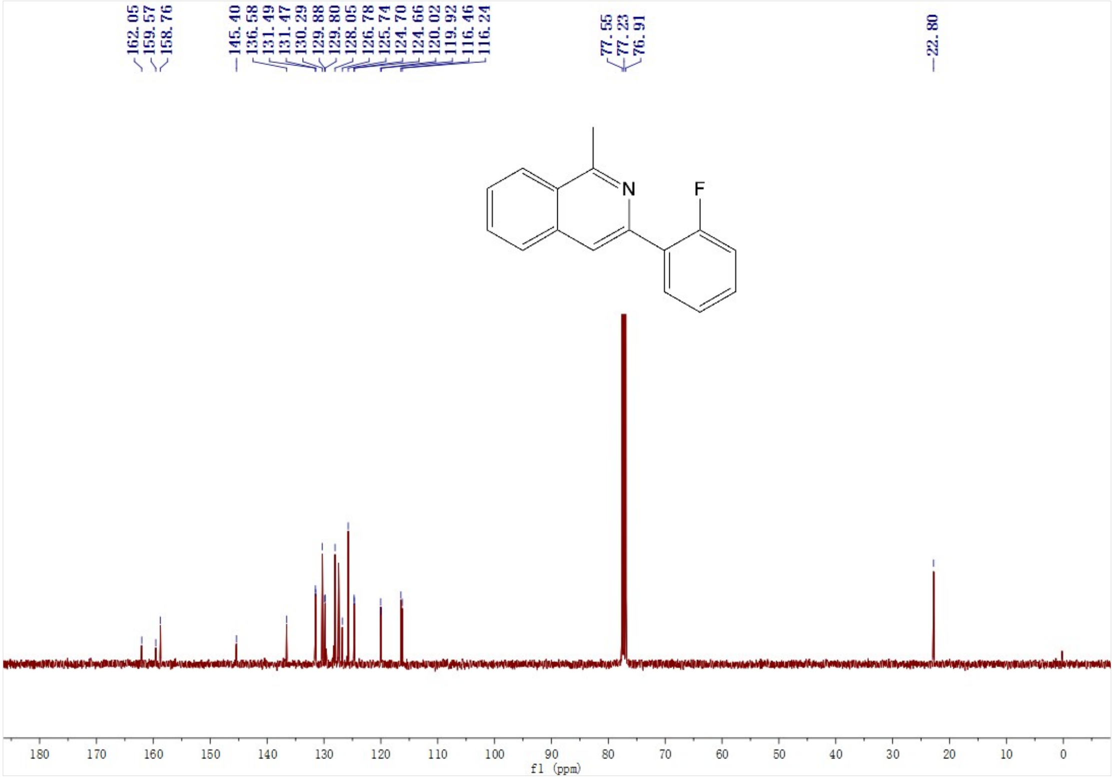

^{19}F NMR spectra of **3am**

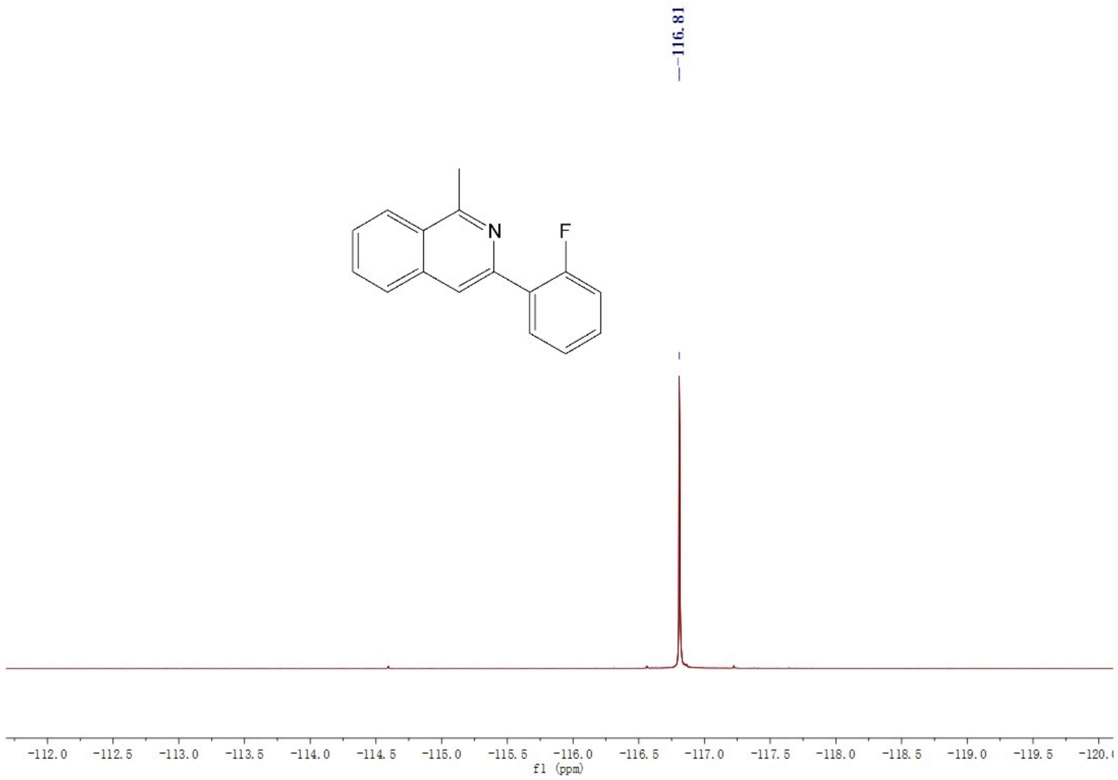

^{1}H NMR and ^{13}C NMR spectra of **3an**

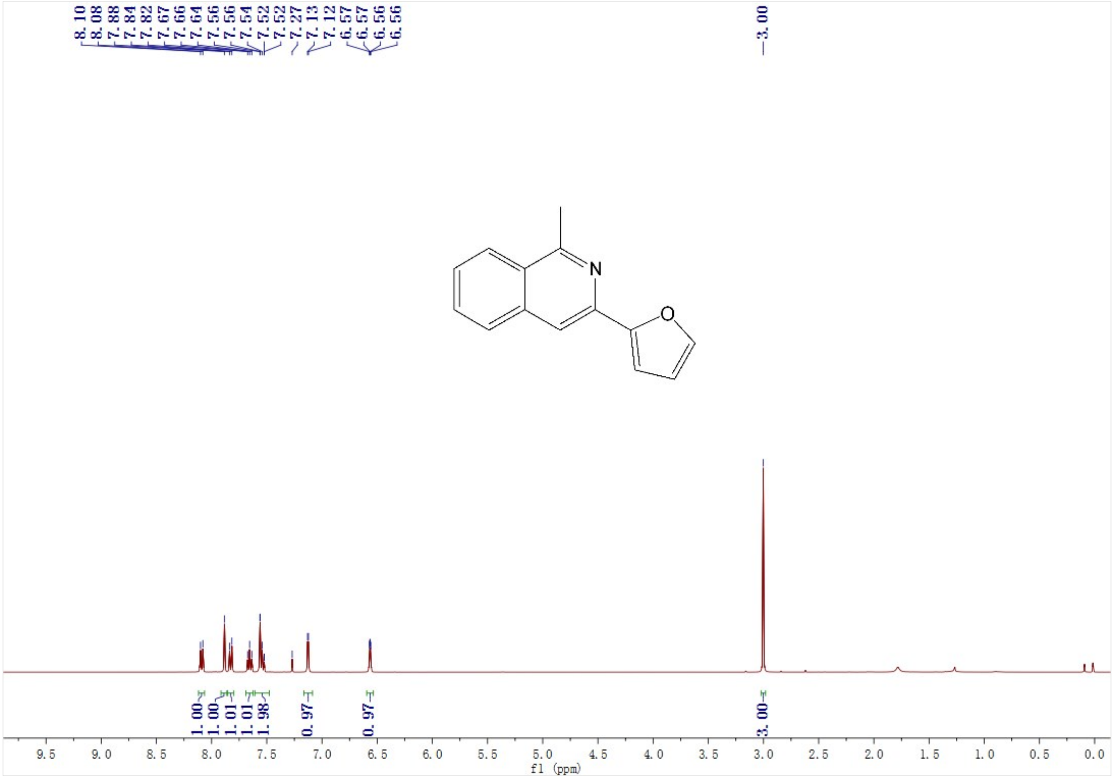

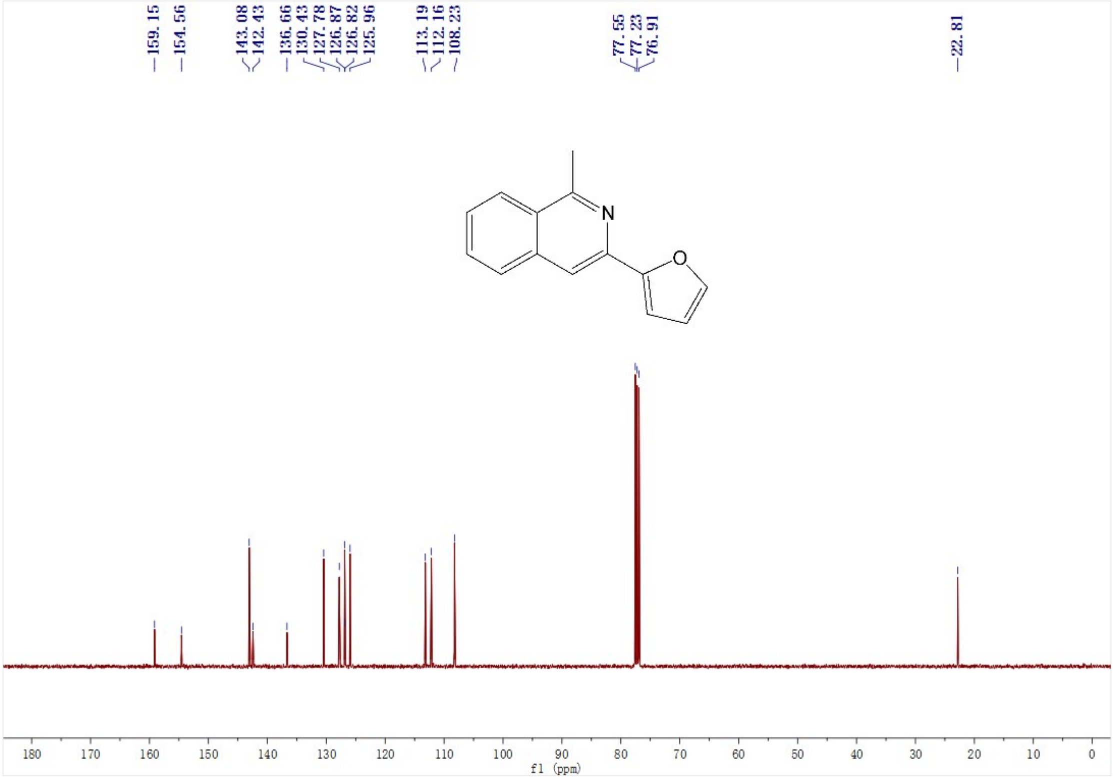

^{1}H NMR and ^{13}C NMR spectra of **3ao**

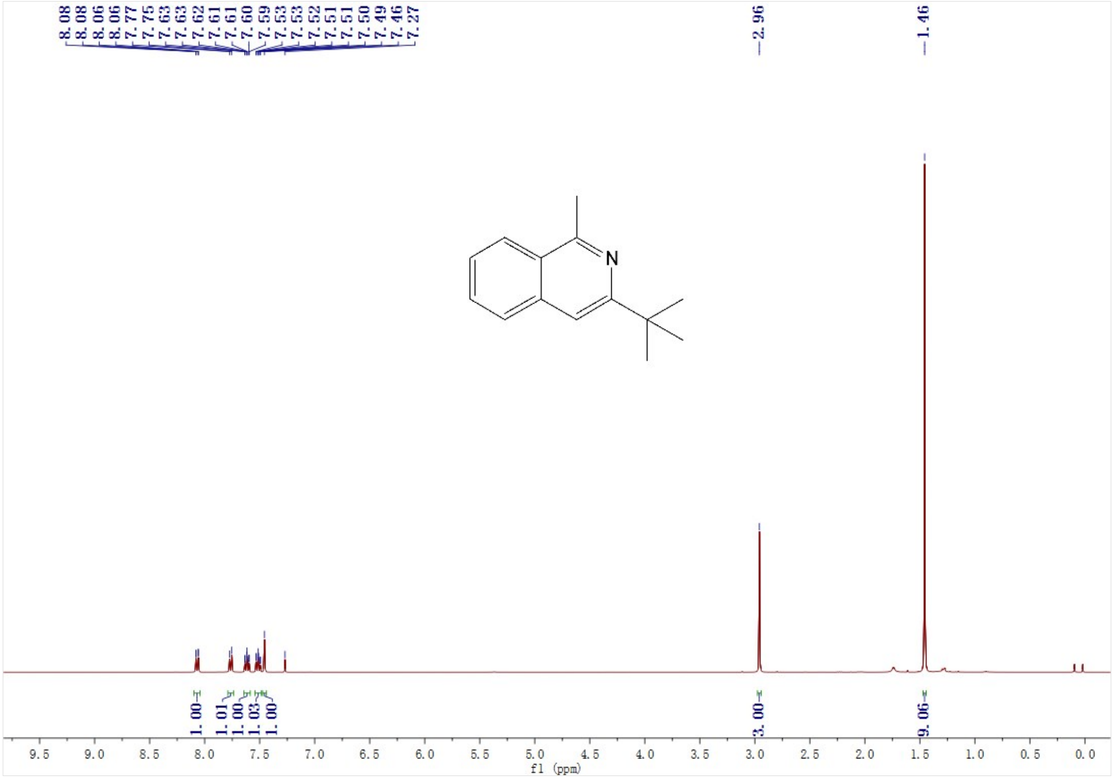

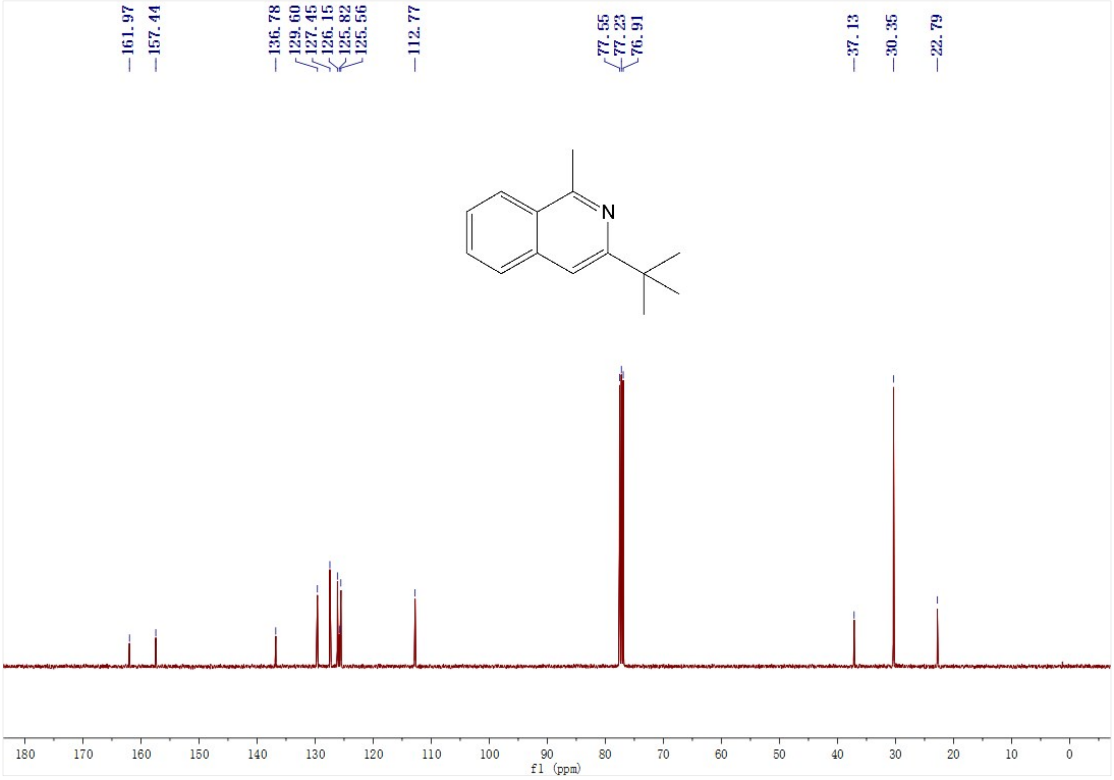

^{1}H NMR and ^{13}C NMR spectra of **3ap**

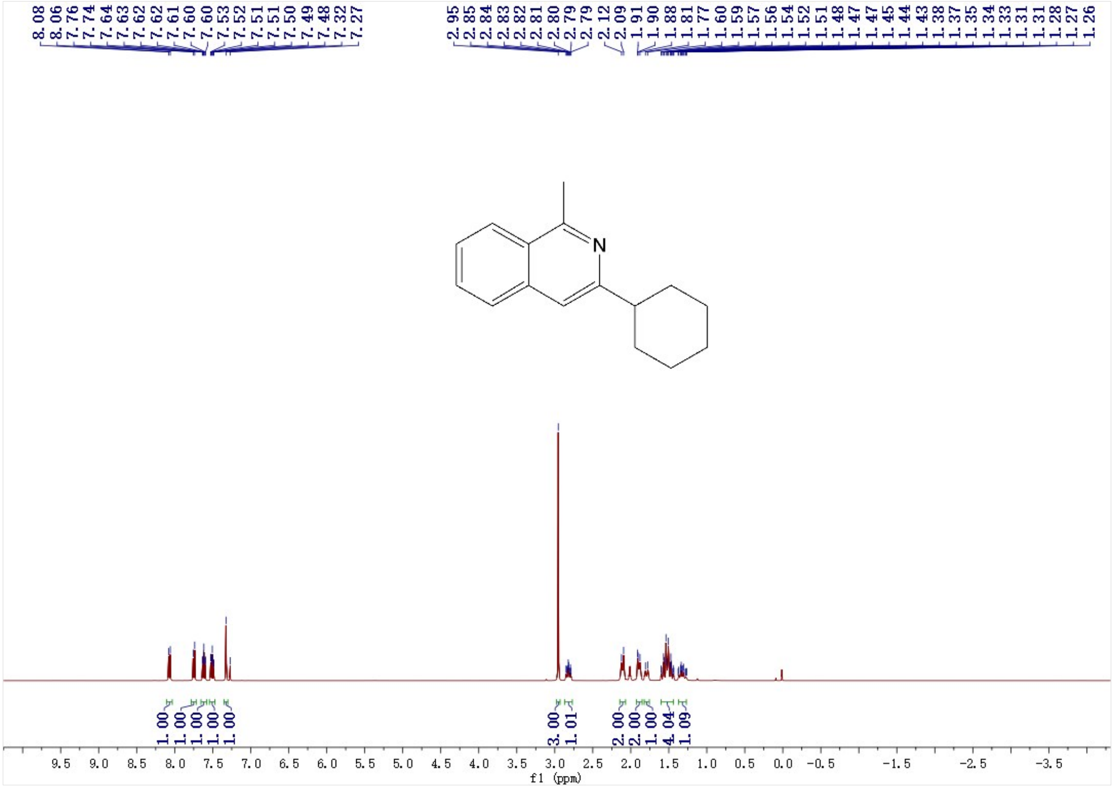

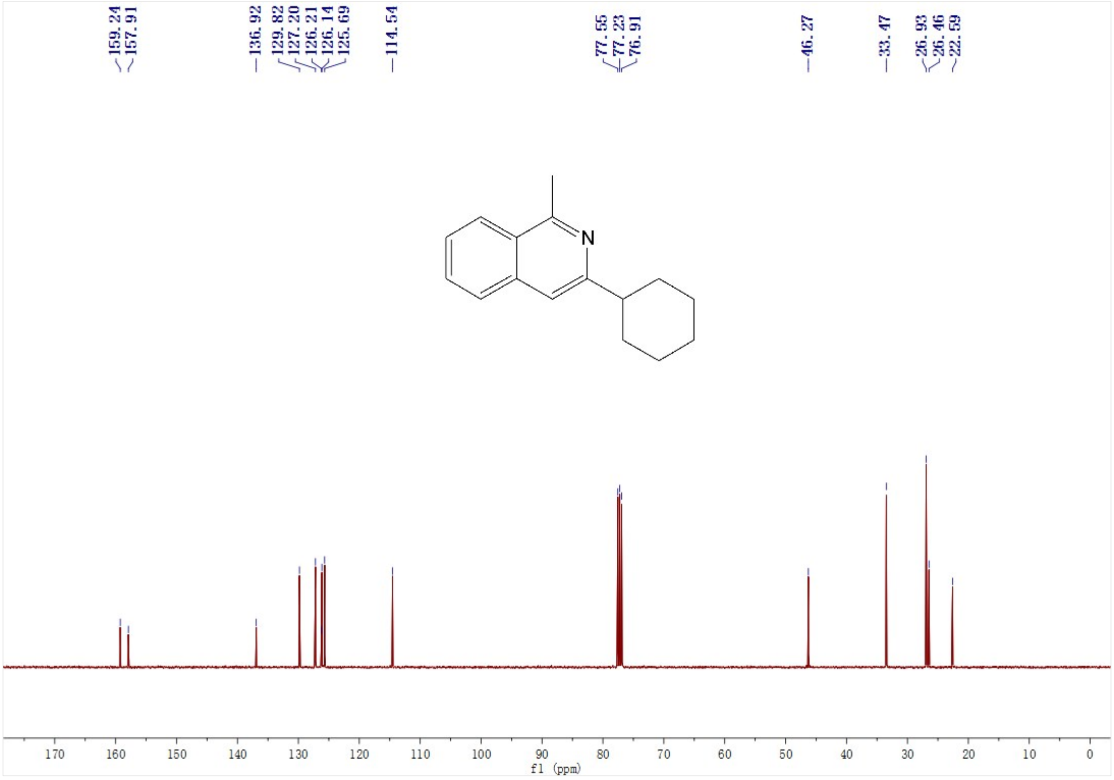

^{1}H NMR and ^{13}C NMR spectra of **3aq**

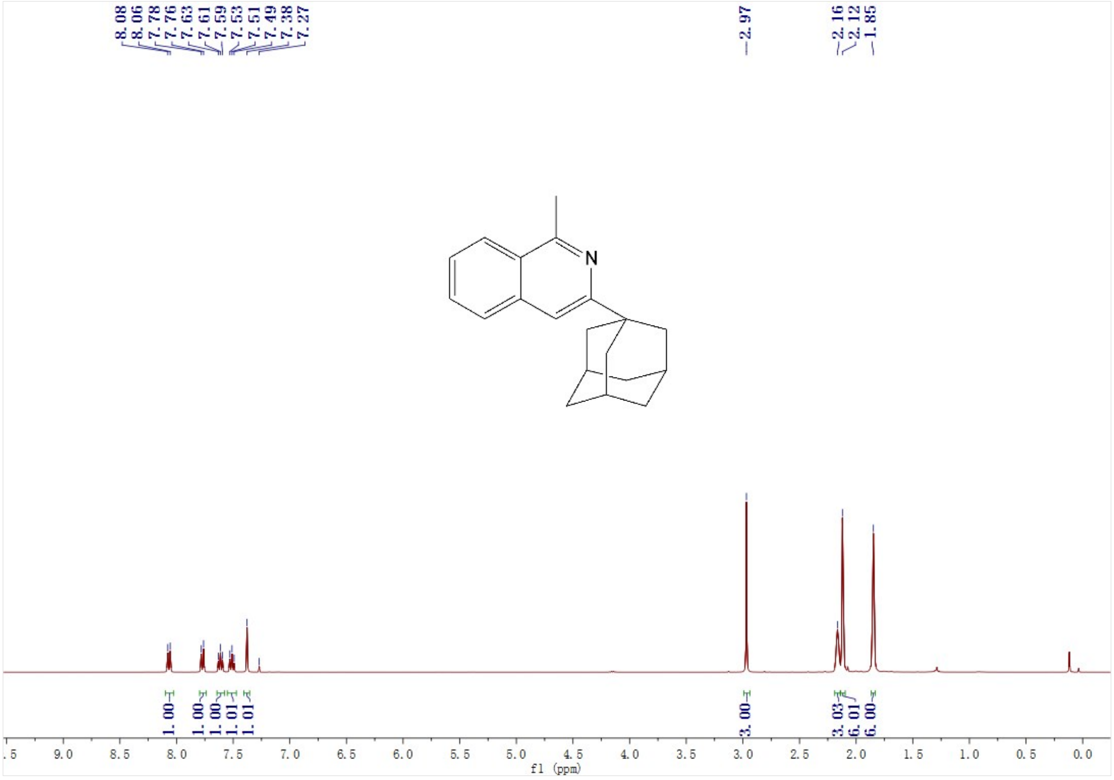
8.08 8.06 7.78 7.76 7.63 7.61 7.59 7.53 7.51 7.49 7.38 7.27
2.97
2.16 2.12 1.85
N
1.00 1.00 1.00 1.01 1.01
3.00
3.03 6.01 6.00
f1 (ppm)

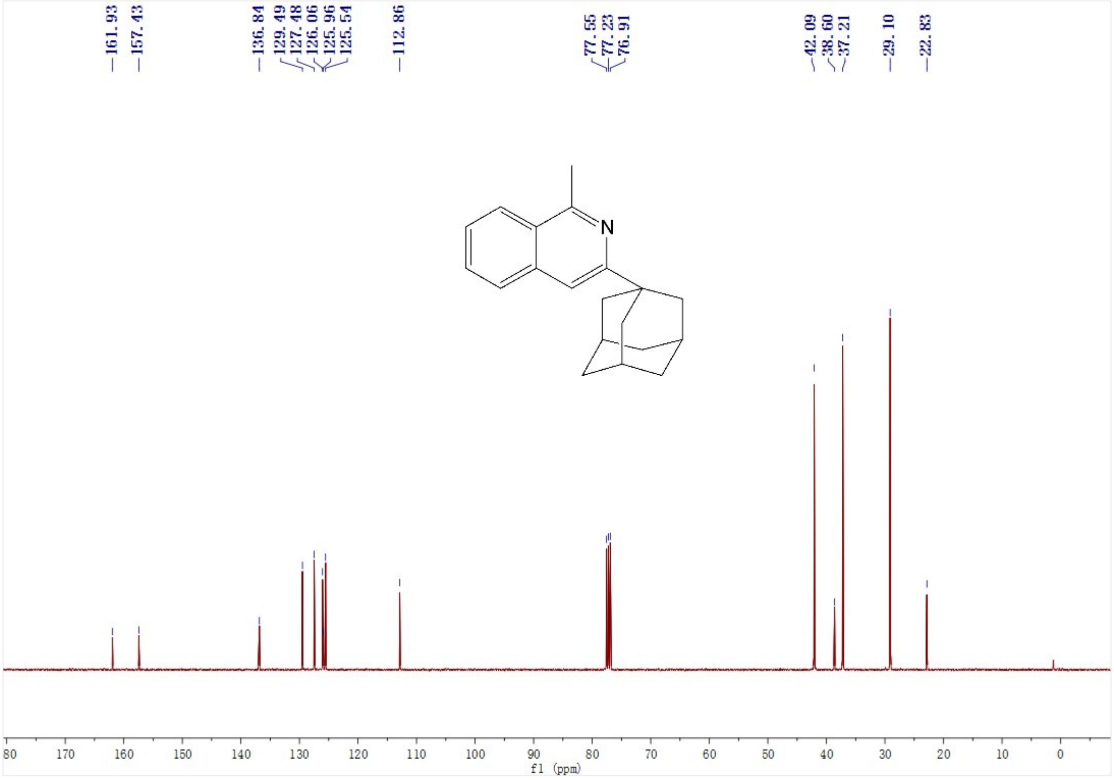
161.93 157.43
136.84 129.49 127.48 126.06 125.96 125.54
112.86
77.55 77.23 76.91
42.09 38.60 37.21
29.10 22.83
N
f1 (ppm)
